# Participant Index

**DOI:** 10.1093/geroni/igaf122.4415

**Published:** 2025-12-31

Arabic numbers refer to session numbers.

Duplicate numbers indicate the participant is listed as an author on multiple abstracts within a session.

A

Aaron, Siobhan: 8010

Aartsen, Marja: 8000

Aasaavari, Asmita: 1315, 7360

Abadir, Peter: 3390

Abaei, Elnaz: 1065, 4600, 8005

Abbott, Alyssa: 8000

Abbott, Katherine M: 1300, 2490, 7305, 8015

Abdel Magid, Hoda Samir: 2005, 2400

Abdelfattah, Lindsey: 4065

Abdelgawad, Norhan: 4007

Abdi, Lovina: 7240

Abdullah, Saeed: 8000

Abi Nassif, Alexandra: 1495

Ablah, Elizabeth: 7180

Aboagye, Shelly: 2175, 4190

Aboagye Kyei, Amma S: 8000

Abraham, Alison G: 9130

Abrahams, Loes: 7035

Abrahamyan, Lusine: 7090

Abrams, Grace: 2275

Abrams, Wendy: 9080

Abramson, Rachel H: 8000

Abramson, Tobi: 8010

Abreu, Taiane: 7380

Abrials, Josephine M: 8015

Abshire Saylor, Martha: 4450

Abu, Hawa O: 2500, 7410, 7485

Abul, Zeynep: 7255

Acharya, Bibhav: 8015

Acharya, Sharmila: 7100

Acierno, Ron: 4415

Ackerman, Michelle L: 7175

Ackley, Sarah: 4280

Adamek, Margaret E: 4070

Adams, Anne E: 9065

Adams, Swann: 7220, 8000

Adams, Sydni N: 8010

Adams-Campbell, Lucile: 7190

Adams-Price, Carolyn: 8015

Adandom, Isreal: 7300

Adaniya, Stephanie: 8000

Adar, Sara D: 1040

Addepalli, Venkatanand ram: 9070

Addison, Odessa: 7140, 7905

Adebusoye, Lawrence A: 4565

Adegoke, Elikanah Olusayo: 7315

Adeleke, Bisola C: 4420

Adelman, Ronald D: 1170

Adelnia, Fatemeh: 3260

Adeniji, Dolapo O: 4070

Adepoju, Omolola E: 3305, 4395

Aderonmu, Aderonke: 4190

Adfares, Hadil: 7295

Adjei, Mathias: 7065, 7240

Adjei, Naomi: 4150, 8010

Adjei Gyimah, Akwasi: 7065, 7240

Adkins-Jackson, Paris B: 2005, 3345, 4125, 7470, 7535, 7900, 8015

Adomako, Jennifer Asaa: 8000

Adutwum, Frank: 8000

Aelick, Katelynn: 7075

Afonso, Tara McBride: 1050

Agarwal, Yash: 9070

Agbawodikeizu, Patricia U: 1365

Agbomi, Lidadi: 8015

Aggarwal, Neelum: 4570

Agha, Leila: 8000

Aghjayan, Sarah: 4585

Agmon, Maayan: 1110, 2105, 2415, 7040, 7210

Agrawal, Shubham: 3340

Agrawal, Sunil K: 7025

Aguila, Emma: 2060, 8015, 9075

Aguilar, Vanessa: 4475

Aguillon, David: 8000, 8015

Aguilo, Maria Elisa: 1390

Aguirre, Alyssa: 4015, 8015

Aguzzoli Peres, Fernando: 7010

Agyekum, Boadi: 4470

Ahmad, Anam: 7315

Ahmad, Fyzeen A: 2330

Ahmad, Waqar: 4550

Ahmed, Ali: 2460, 8010, 9020

Ahmed, S M Foysol: 7450

Ahmed, Sohel: 1375

Ahn, Bumsoo: 4275

Ahn, Hyochol: 7010, 7500

Ahn, SangNam: 7205

Ahn, Sangwoo: 4580

Ahn, Seoyeon: 7275, 7470, 7925, 8005

Ahn, Soojung: 2190, 2255, 8015

Ahn, Sumyeong: 3090

Aichele, Stephen: 8015

Aideed, Ayanle: 8000

Aikawa, Sora: 2440, 8010

Aiken, April: 8015

Ailshire, Jennifer: 1415, 2250, 2270, 2275, 2345, 2400, 8015

Ainsworth, Laura A: 7350

Ajay, Sreya: 8005

Ajayi, Oluwaseun K: 4565

Ajrouch, Kristine: 3230, 4285

Akar, Fatma: 8005

Akash, Moniruzzaman: 4005

Akenine, Ulrika: 4370

Akiko, Furuta: 7440

Akincigil, Ayse: 2185, 4210

Akmal, Allie: 2400

Akman, Jasmine: 4210

Akorlatse, Raymond: 8000

Akter, Nahida: 1005, 7305

Akter, Rejina: 8015

Akter, Salma: 1005

Akudugu, John: 9030

Akushevich, Igor: 1235, 4010, 4460, 7185

Akwobugi, Moses: 1145, 8000, 8015

Akyildiz, Erdogan Oguzhan: 7315

AL Harrasi, Azza M: 8015

Al Juboori, Ruaa: 4430, 7220

Al Snih, Soham: 7225, 7230, 7255, 8005

Alabi, Aderonke A: 4565

Alagappan, Cecilia M: 7135

Alam, Ishrat: 7485

Alam, Marzan: 4005

Alamian, Arsham: 7425

Albala, Cecilia: 9035, 9125

Albashayreh, Alaa: 8000, 8015

Albaum, Jason A: 8010

Albert, Marilyn S: 9045

Albert, Steven M: 4305

Alberth, Andrew: 7275, 7285

Albertini, Marco: 4575, 7210, 9020

Albrecht, Jennifer S: 2035, 8010, 8015, 9005

Aldridge, Georgina: 8005

Aldridge, Melissa D: 2185

Aldwin, Carolyn M: 3330

Alea, Nicole: 7005, 7935

Alejo, Cherrylyn: 1050

Alejo, Prince M: 4350

Aleksic, Sandra: 8000

Alemi, Farrokh: 2125

Alex, Danny: 9135

Alexander, G Caleb: 7255

Alexander, Gregory L: 7125

Alexander, Karah L: 8010

Alexander, Neil B: 4060, 7225, 8010

Alexander, Rachael: 1025

Al-Hadhrami, Samiya: 8015

Alhay, Zahra: 7415

Ali, Farwa: 7025

Ali, Talha: 8000

Ali, Waad: 4470, 7040

Alio, Paula A: 3165

Aljohani, Rania: 8010

Allan, Alexa C: 3060, 7325

Allard, Eric: 9055

Allebach, Phoebe: 4130

Allen, Elizabeth M.: 4590

Allen, Erica: 7455

Allen, Julie Ober: 1095

Allen, Larry A: 7085, 7910

Allen, Laura D: 8000

Allen, Priscilla D: 7355

Allen, Quinn: 7375

Allen, Rebecca S: 7310

Aller, Ty: 7215, 7305

Allison, David B: 2560

Allison, Theresa A: 2515

Alloy, Linda: 1130

Allsopp, Richard: 7240

Alm, Kylie H: 1155

Almeida, David M: 1175, 1375, 1480, 2095, 2280, 3330, 4000, 4120, 4520, 7365, 7380, 8000, 8005, 9015

Almeida, Quincy: 7225, 7905

Almidani, Louay: 7530

Almroth, Melody: 1425

Al-Naggar, Iman: 1387, 8005, 9090

Alnajar, Malek: 1025, 2190

Alosco, Mike: 1510

Alpizar, Olivia: 8010

Alquist, Jessica: 4590

Alqurashi, Mohammed: 8005

Alsamawi, Amal: 1315, 7360

Alshahwan, Sandi: 8010

Alston, Daniel: 2330

Altizer, Jasmine Travers: 1435, 2440

Altona, Janissa: 3035

Alvarez, Jessica: 8015

Alvarez, Loreli: 2030, 4140, 8010

Alvarez-Alvarado, Stacey: 1285

Alvarez-McNelis, Desiree: 7465

Alving, Loren: 7135

Alzahrani, Mallak: 1220, 2510

Amadae, Sonja M: 7275

Amagasa, Shiho: 7345

Amano, Takashi: 2025, 8015

Amar, Shimon: 7135

Amaravadi, Harsha: 8000, 8010

Ambadar, Zara: 2375

Ambellu, Gelila: 8015

Ambrosio, Fabrisia: 4473

Amer, Hana A: 7125

Ames, Jesse: 1455

Amin, Iftekhar: 7145

Amin, Suhani: 8010

Amini, Reza: 7050

Amir-Koren, Uri: 4130, 4210

Amjad, Halima: 2035, 7010, 7910, 8000, 8015

Amland, Rachel: 7415

An, Hyunjoo: 7355, 7470

An, Ning: 8000

An, Ruopeng: 7015

An, Yang: 4495

An, Yinong: 7340

Anaab, Esme: 7540

Anand, Akshay: 9070

Ances, Beau M.: 8015

Andel, Ross: 3370, 7295, 7365, 7375, 8000

Andersen, Dan: 8010

Andersen, Stacy: 1520, 4440, 7310, 8005, 8015, 9155

Andersen, Troy C: 8000

Anderson, Brittany: 8005

Anderson, Emma: 1350

Anderson, Joel G: 1000, 1420, 2010

Anderson, Keith A: 2455, 4145

Anderson, Linda K: 8010, 9140

Anderson, Loretta: 4070

Anderson, Lyndsey M: 7335

Anderson, Rose: 1240

Anderson, Rozalyn: 3267

Anderson, Ruth A: 2150, 2255, 2475, 4095

Anderson, Sarah: 8010

Anderson, Shannon: 2255

Anderson, Timothy S: 1485

Anderson-Hanley, Cay: 7545, 8005

Ando, Fujiko: 7330

Andonian, Brian J: 2380, 4525

Andrade, Flavia D: 2060, 3265, 7405, 8015

Andrango, Lizette: 7455

Andreoletti, Carrie L: 4007

Andrews, Heather: 7435

Andrews, Ryan: 2000, 4540

Andrews, Sophie C: 4580

Aneja, Ritu: 3095

Anetzberger, Georgia J: 3350

Anfang, Sophie: 7525

Ang, Ian Yi Han: 8005

Ang, Shannon: 1205, 3325

Angel, Jacqueline L: 2060, 3265, 3395, 7475

Angelini, Whitney: 9080

Angell, Sarah C: 3315

Angrisani, Marco: 3010, 4490

Ani, Judith I: 8015

Aniemeke, Chinenye C: 1430

Anil Rao, Anchala: 8000

Ankuda, Claire: 2185, 4430, 8010

Anquillare, Elizabeth: 7200, 7395

Ansah, John Pastor: 2435

Ansari, Shadi: 4335, 7390

Ansong, Rockson: 8015

Anstey, Kaarin J.: 4470

Antic, Mina: 3330

Antonios, Vera: 2395

Antonio-Villa, Neftali Eduardo: 1155

Antonucci, Toni: 3230, 4285, 4490, 8000

Antunes, Kristen: 7230

Anurum-Anyanwu, Christina: 7535

Aoun, Christy: 8010

Aoun, Layana: 8010

Aoun-Barakat, Lydia: 7245

Aparicio, Hugo: 9105

Aparicio, Triccia: 8010, 8015

Apostolou, Hannah L: 7310

Apostolova, Liana: 7010, 7910

Appelhans, Bradley M: 1110

Applebaum, Allison J: 2175

Applebaum, Robert: 4260, 7250, 7540

Applegate, Julia M: 1000

Appleton, Allison: 8015

April-Sanders, Ayana: 9045

Arai, Yasumichi: 7365

Aranda, María P: 1055, 2060, 3265, 3370, 3395

Araujo, Lia: 2120, 7380

Araujo Menendez, Carlos: 8010

Araujo-Menendez, Carlos: 8005, 8015

Aravena, Jose M: 9035, 9125

Aravena, Maria: 9035

Arbeev, Konstantin: 7415

Arciero, Paul: 8005

Ardekani, Azam: 7050

Ardisson Korat, Andres: 4440, 9155

Arena, Aprille: 1515, 8005

Arfanakis, Konstantinos: 4570

Arguelles-Borge, Soledad: 7005, 7110

Argueta, Daniel: 9015

Argueta, Daniel L.: 8000

Arias, Dalmina: 3265

Arieli Zaks, Efrat: 9095

Aris, Izzuddin: 1225

Arling, Greg: 7370

Arlyck, Ralph: 4115

Armant, Ryan: 8015

Armbruster, Bill: 4130

Arms, Tamatha E: 8015

Armstrong, Alexandra: 8010

Armstrong, Brittany D: 7435, 8005, 8015, 9025, 9130

Armstrong, Grace Q: 1485, 7410

Armstrong, Melissa: 7350

Arnett, Marieh: 8000

Arnette, Shannon: 7005

Arnold, Michelle L: 4065

Arnold, Sophie: 7030

Arnold, Steven E: 7325, 7415

Arntsen, Bjørnulf: 7205

Arogundade, Odunayo T: 7150

Aron, Sophia: 1280

Aronson, Louise: 8000

Arora, Arushi: 1270

Arora, Kanika: 1500, 7300, 7515

Arora, Vineet: 7430

Arpawong, Thalida: 3127

Arroyo, Edmond M: 4350

Arruda Franco, Gustavo: 8010

Arteaga, Ignacia: 7135

Arthanat, Sajay: 2410, 4005

Artoni, Filippo: 7315

Arulraj, Theinmozhi: 7315

Aruscavage, Nancy: 8015

Arya, Richa: 7170

Asakitogum, David: 7425

Asante, Samuel: 7065, 7240

Aschenbrenner, Andrew: 7505

Aschwanden, Damaris: 1480

Ashcraft, Alyce S: 7125

Ashcraft, Laura Ellen: 8015

Ashe, Maureen C: 4490

Ashebir, Sophia: 3250, 4410, 7005

Asher, Merav: 2415

Ashida, Sato: 2135, 4350, 8000

Ashiqueali, Sarah: 4347

Ashirifi, Gifty D: 4070

Ashish, Dev: 7010

Ashton, Abbie: 4450

Ask, Levi: 1255

Aslam, Hamza Saghir: 2315

Asma, Khunza: 7425

Asoni, Gesuina: 7265

Asraf, Kfir: 1110

Assakul, Rarinsri P: 1275

Asthana, Sanjay: 4585

Astone, Kristina: 4535

Asundi, Archana: 7245, 7915

Aswani, Truphosa (Posa): 2010, 3155

Atherton, Olivia E: 4110, 8015

Atkins, Peter: 7300

Atkins, Shelbie: 7500

Atshan, Samer: 1040, 1330

Attili, Noura: 8005, 8015

Au, Rhoda: 1510

Austad, Steven N: 2560, 4347

Austin, Nadine C: 1315

Au-Yeung, Wan-Tai M: 7335

Avalos, Maria: 2285

Avelino-Silva, Thiago Junqueira: 4425, 8005

Avent, Elizabeth S: 2295, 3300

Averbukh, Maxim: 4275

Aviña, Sarah: 4260

Avila, Ivan: 8010

Avila, Jaqueline: 7200

Ávila-Funes, José A: 7145

Ávila-Pacheco, Julián: 9155

Avila-Rieger, Justina F: 3380, 7040

Avorn, Jerry: 7510

Awad, Rabab: 7060

Awata, Shuji: 7300

Ayalon, Liat: 2250, 2370, 3080, 3305, 4395

Ayaz, Hasan: 4525

Aycock, Dawn: 3040

Ayele, Roman A: 8005

Azar, Ariel: 3195

Azhar, Esha: 1330, 7035

Azhar, Gohar: 7005, 7010, 7190, 8015

Azuero, Andres: 2030, 4140, 9125

Azuz, Anna: 8010

B

Babaieasl, Faezeh: 8005

Babar, Huma: 2175

Babenko, Natalia: 7175, 7425

Babulal, Ganesh M: 1355, 7505, 8015, 9015

Baby, Bincy: 7230

Baby, Eliza: 9085

Baca, Hailey: 8010

Bachmeier, James: 4290

Bachmeier, Natalie: 4075

Bachner, Yaacov G.: 2370, 7110

Back, Hayun: 8010

Backes, Ingrid: 1215, 9105

Bäckman, Malin: 3145

Backus, Deborah: 1230, 7225

Bacsu, Juanita-Dawne: 2070, 7205, 7220, 8015

Badell, Camila S: 8010

Bae, Elizabeth: 1520, 7480

Bae, Harold: 8005

Bae, Jungyeon: 7295

Bae, Myeongjin: 7265

Bae, Suyeong: 7335

Baehr, Laura: 2380, 4525

Baek, Jihye: 7290

Baek, Jimin: 3235

Baek, Jiwon: 8005

Baek, Jiyeon: 7190, 8010

Baek, Jonggyu: 7255

Baek, Sehyun: 8000, 8005, 8010, 8015

Baek, Seoyoung: 7245

Baena, Ana: 8000

Bagaajav, Ariunsanaa: 8015

Bagley, Olivia: 7415

Bagott, Darcie: 4565

Bahrami, Raheleh: 7305

Bai, Chen: 7145

Bai, Jing: 4575

Bai, Xue: 1515, 2315, 7405, 8010, 9050

Baier, Rosa R: 2055, 4475

Baig, Farah: 3000

Baik, Dawon: 7085, 7910

Baik, Sol: 7260, 7295, 8010

Bailey, Christine: 1315, 7360

Bailey, Regan L: 7480

Bailey, Ryan: 7325

Baillargeon, Emma: 8010

Baim-Lance, Abigail: 4425, 9145

Baker, Elizabeth: 3095

Baker, Jillian S: 1110, 4460

Baker, Jordan: 7265

Baker, Kristin: 1385, 2185

Baker, Tamara A.: 3395, 4095

Baker, Zachary G: 1440, 3375, 8005

Bakerjian, Debra: 2410

Bakitas, Marie: 4445

Bakker, Arnold: 1155

Balachandran, Anoop T: 8010

Balachandran, Arun: 2105, 8000, 8010, 9135

Balasubramanian, Raji: 8005, 9155

Balbale, Salva N: 7185

Bald, Sarah: 8015, 9155

Baldus, Clara M: 3355

Baldyga, Kathryn: 7130

Balk, Deborah: 3385

Balkan, Em: 1465, 7485, 7930

Ball, Karlene: 4030

Ball, Sarah: 1485

Ballew, Shoshana H: 3105, 4450

Ballhausen, Nicola: 1240

Balmuth, Alexa: 3250, 4410

Balog, Emily: 8010

Balser, Sarah: 3245

Balthazar, Monique: 7535

Balz, Magdalen A: 7040

Bámaca, Mayra: 7470

Bambury, Elizabeth: 7180

Bamgbade, Benita A: 8015

Bamonti, Patricia M: 2240, 7265

Banarjee, Chitra: 3030, 7225, 7495, 8005, 8015

Banarjee, Reema: 7315, 7480

Bandeen-Roche, Karen: 2100, 2510, 3345, 4025

Band-Winterstein, Tova: 7450

Bandyopadhyay, Attrayee: 1025

Bane, Scott: 9135

Banerjee, Samprit: 4225

Banerjee, Seema: 7530

Bangerter, Lauren R: 1035, 3340, 4015

Bangs, Alex: 7415

Baniassadi, Amir: 3235, 7295, 7465

Bankole, Azziza O.: 8000

Bankovska, Maryna: 4470, 7210

Banno, Ryoichi: 7480

Bansal, Ophira: 9125

Bao, Jiaxing: 9155

Bao, Qintong: 7310

Bao, Shaorui: 7285

Bapna, Priya: 1470

Barajas-Gonzalez, R. Gabriela: 3155

Baral, Suvab R: 4200

Baranes, Guy: 2245

Baranzini, Mirko: 8000

Barbabella, Francesco: 7070

Barbakoff, Daniel: 7300

Barbara, Bassola: 8015

Barber, Malisa: 9090

Barber, Sian: 1365

Barbera, Mariagnese: 1290

Barbieri, Marielena: 7380

Barbosa, Paulo: 8005

Barbosa Suffredini, Ivana CC: 7300

Barczi, Steve: 3005, 3115, 4350

Bardales, Rhonda: 7350

Barghout, Rana: 8005

Barha, Cindy: 4230

Baris, Veysel: 8015

Barker, Jennifer M: 8010

Barker, Megan: 7290, 8000

Barnes, Deborah E: 2245, 9090

Barnes, Lisa L: 1415, 2025, 2200, 3130, 3395, 4570, 8005

Barnett, Michael: 3165, 4240

Barney, Ann C: 7500

Barooah, Adrita: 8000

Barouch, Stav: 4355

Barp, Milara: 8000

Barr, Paul J: 2045

Barr, Paul: 8015

Barr, Paul J: 9045

Barr, Robin: 2215

Barragan, Noel: 7225, 7905

Barreto Garcez, Flavia: 8005

Barrett, Anne E: 4245

Barrett, Austin: 4020

Barrett, Donna: 4600

Barrett, Kat: 1285, 8000

Barrett, Tyler: 9090

Barrie, Fatmata M: 7500

Barrios, Veronica: 2325

Barron, Yolanda: 8005

Barrow, Jennifer: 7385

Barry, Lisa C: 1110, 1315, 2185, 7205, 7345, 8010

Bartels, Stephen: 1160

Bartlett, Paige: 7100

Bartley, Jenna: 8000, 8010

Bartman, Sydney: 1520

Barton, Kelli: 7205

Barzilai, Nir: 4495, 8015

Barzilay, Shira: 4185

Basch, Arielle: 7410

Bashian, Hannah: 1050, 2240

Basic, Ajla: 7440

Basisty, Nathan: 7315, 7480

Basnet, Lila: 1500

Basrai, Zavera: 7075

Bass, David: 2285, 7010, 7915

Basu, Rashmita: 3310, 7010

Basu, Shayna: 7265

Batchek, Dakota: 1220

Bates, Timothy: 4260

Batista-Malat, Eleanor: 1215, 7230

Batsch, Nicole: 4160

Batsis, John A: 1220, 1235

Battle, Kimberly: 1260, 7095

Batura, Josey: 4085, 7330

Bauer, Julia A: 2040

Baughman, Kristin: 7225

Bauldry, Shawn: 1195, 4315

Baumgart, Matthew: 4160

Baxter, Samuel: 8005

Bayless, Teah: 7520

Bayliss, Elizabeth: 4090, 7385

Bayne, Alycia: 4435

Bayne, Kozbi L: 7440, 9110

Bea, Megan: 3285

Beaird, Genevieve: 1260, 7095

Beamer, Brock: 2125, 8010

Bean, Jonathan F: 7040, 7300, 8010

Bean, Jonathan F: 8010

Bean, Jonathan F: 9095

Beard, Renee L: 7470

Bearden, Valerie: 7015, 7935

Beason, Asia L: 7210

Beattie, Zachary: 7335, 7545

Beatty Moody, Danielle L: 2400

Beauchamp, Jennifer ES: 4415

Beauchamp, Marla: 7225, 7905

Beaufils, Constance: 3225, 9040

Beaulieu, Chris: 8015

Beavers, Daniel P: 7165, 8005

Beavers, Kristen: 7165, 8005, 8015

Bechara, John: 7035

Bechek, Bonnie: 8000

Becher, Robert D: 1375

Beck, Brianna: 9015

Beck, Emorie D: 1265, 4120

Beck, Maureen: 8000

Beck, Mike: 2220

Beck, Samuel: 8010

Becker, Heather: 7420

Becker, Marigrace: 2420, 8000

Becker, Todd D: 2320, 4430, 7260, 7500

Beckert, Angela: 7455

Beckett, Cameron G: 1315

Bedi, Anaika: 8000

Bedjeti, Katy: 4120

Beebe, Timothy: 2160

Beeber, Anna Song: 1270, 2150, 4070, 4095, 9065

Beech, Bettina M: 3180

Beem, Daniel K: 4405

Beenhouwer, David: 8000

Beer, Jenay M: 8015, 9065

Beesley, Hannah: 8010

Begum, Momotaz: 4005

Behnken, Emma: 8005

Behr, Grace: 9075

Behrens, Liza L: 1005, 2170, 3155, 4480, 8015

Bei, Eva: 4575

Beidelman, Erika T: 1495, 9040

Beiser, Alexa: 8010

Beisheim-Ryan, Emma H: 7140

Bekelman, David: 4430

Bekena, Semere: 8015, 9015

Belanger, Brigitte: 7485

Belanger, Emmanuelle: 2160, 4330, 7185, 7220

Belanger, Vi: 8015

Beldeen, Jacqueline: 8000

Belin de Chantemele, Eric: 7245

Belinson, Natella: 1515

Bell, Bethany A: 4125

Bell, Janice F: 2450, 4145

Bell, Julia: 7370

Bell, Mallory: 1080

Bell, Mary Rose E: 8000

Bell, Sarah Beth: 1510

Bell, Tyler R: 3370, 4030, 4560, 8000, 8015

Belleguic, Thierry: 8005

Belli, Hayley M: 2380

Belli, Robert: 2400

Bello, Ghalib: 8010

Bello-Chavolla, Omar Yaxmehen: 1155

Bellolio, Fernanda: 9045

Bellury, Lanell: 2255

Belnavis, Alexander: 7315

Belsky, Daniel: 8000, 8010, 9135

Beltran, Ana: 7470

Beltran, Susanny J: 2265

Belyea, Loni: 7030

Belza, Basia: 3205, 4535, 7100, 7415, 8015, 9005

Ben Harush, Odelia: 7145

Benavides, Megan: 1090

Ben-David, Boaz M: 4235, 9095

Bender, Alexis A: 2160, 4135

Bender, Alexis A: 9080

Benedict, Claire: 4035

Benge, Jared: 4015

Benheim, Talia S: 7050, 8000

Benjamin, Alyssa: 8005

Benjamin, Andrea Z: 7000

Benjamin, Emelia J: 7120, 8000

Benjasirisan, Chitchanok: 7380, 7430, 7495

Benloucif, Slim: 8005

Benmar, Claudine: 8010

Bennett, David A: 2460

Bennett, Henrietta A: 4085, 8000

Bennett, Jill: 4470

Bennett, Katherine: 2205, 8010, 8015

Bennett, Stephanie: 7015, 9025

Bensley, Kara: 9015

Benson, Bill: 8005

Benson, Clark: 7000

Benson, Jacquelyn: 7260, 8000

Benson, Lizbeth: 8000

Benton, Donna: 2390, 3300, 7085

Benyamine, Mohamed: 4430

Benzel, Heather: 1305

Benzo, Roberto: 2220

Berardi, Angela: 9020

Berdugo-Hernandez, Stephanie: 7340, 7920

Beresford, Lea: 7005

Berg, Anne Ingeborg: 4010, 7255

Berg, Hayley: 8015

Berg, Karina M: 1110, 7305, 9090

Berg, Kristen: 8005

Bergee, Rebecca E: 9025

Bergen-Jackson, Kimberly M: 7250, 7330

Berger, Ford: 8010

Bergman, Yoav S: 1100, 1330, 2105, 2390

Bergstrom, Jaclyn: 7050, 7190, 7375

Berg-Wedger, Marla: 3255, 2110, 2335, 8000

Berish, Diane: 3035, 3220, 4380, 7465, 7500, 8000, 8015

Berkley, Amy S: 1150, 1515

Berkowsky, Ronald W: 1135

Berlinger, Nancy S: 2420

Bernacki, Gwen: 4445

Bernard, Kimberly P: 1465

Berner, Kennedy J: 7250

Berner, Laura A: 7375

Bernhardt, Paul: 4560

Berning, Peyton: 7295, 7465

Berridge, Clara: 4200

Berroa, Frandys: 9080

Berry, Carolyn: 1505, 7355

Berry, Meagan: 7495

Berry, Sarah D: 1435, 7345, 7385, 7510, 9080

Berry-Stoelzle, Maresi: 1305

Berry-Stoelzle, Maximilian V: 8005

Berta, Whitney: 3040

Bessen, Sarah: 1075

Besser, Lilah: 2400, 4045, 7040, 8000

Bessette, Lily G: 1485

Best, Krista: 8005

Best, Ryan: 1330, 4420, 7355, 7365, 7435

Best, Thomas: 4560

Bethell, Jennifer: 8015

Bettger, Janet: 4525

Bey, Ganga S: 3185, 7145, 8005

Beydoun, May: 7130

Bhagianadh, Divya: 2185, 4390

Bhananker, Annika: 1090

Bharadwaj, Mythili: 8005

Bharath, Leena: 8015

Bhargava, Mahak: 3095

Bhat, Aarti C: 1375

Bhatia, Ashmeet: 7410

Bhatt, Tanvi: 7225

Bhatta, Tirth: 2500

Bhattacharya, Shelley R: 7225

Bhattacharyya, Kallol Kumar: 4030, 7100

Bhatti, Anam: 8010

Bhattiprolu, Atul K: 7525

Bhauryal, Kajal Dhansingh: 9045

Bherer, Louis: 7225, 7905

Bhin, Sun Suk: 7425

Bholat, Michelle: 2555

Bhowmick, Pallabi: 4080, 4150, 8015, 9065

Bhurtel, Shikha: 2450, 4145

Bian, Jinwei: 4585

Bian, Wenxin: 8010

Bianchi, Carolanne: 7495

Bibbins-Domingo, Kirsten: 7260

Bibbo, Jessica: 1215, 1395, 2485

Bibriescas, Natashia: 2030, 4140, 8010

Bicket, Amanda K: 8010

Biderman, Aya: 7135

Bielak, Allison A M: 4050

Bietsch, Breana A: 4165

Bigger, Sharon: 8015

Biggs, Mary L: 8000

Biglieri, Samantha: 2515

Bigman, Galya: 7130, 7265

Bilau, Ibrahim: 7160

Bilbrey, Ann: 7230, 7305

Billeci, Natasa: 1475, 4230, 7390

Bimman, Rennie: 4575

Bin Hossain, Md Sanzid: 7495

Binaco, Jenna N: 7110

Bindra, Sohum: 7405

Birati, Yosefa: 1485, 7410

Bird, Tess: 2090

Birditt, Kira S: 4195, 4490, 7355

Birkeland, Robyn: 7300

Birthelmer, Sierra: 7390

Bisesti, Erin M: 3020

Bishop, Alex J: 7400

Bishop, Christine E: 3310

Bishop, Jill: 9110

Bishop, Kate: 8000

Bishop, Nicholas J: 1055, 4470

Bishop, Rachel A L: 7145

Bishop, Teddy K: 1060, 4240

Bisoi, Rakshit Raj: 8000

Biswas, Rajib Kumar: 7475

Bivona-Maldonado, Emily: 4225

Bixby, Laurin: 8005

Bixter, Michael T: 8005

Black, Kathy: 4130, 7175

Black, Olivia: 2150

Blacker, Deborah: 7205, 9040

Blackshear, Chad: 7025, 7495

Blackstock, Sheila: 8015

Blackwell, Terri L: 8005

Blackwood, Jennifer: 7160, 8010

Blaes, Anne H: 7425

Blair, Elizabeth: 7170

Blancato, Bob: 3350

Blanchflower, Morgan: 2445

Blanco, Julie: 2110

Blanco, Luisa: 3290

Blank, Sabrina: 2390

Blasco, Drew: 7145

Blaskewicz Boron, Julie: 7095, 7150, 7160, 7390, 7430, 7475, 7515

Blind, Jenna: 2440

Blind, Melissa: 1160, 2170

Blinka, Marcela D: 2035

Bloch, Randall: 8005

Block, Laura: 1025, 2135, 7000, 7370, 8005

Blocker, Erin: 1020

Blodgett, Nicole: 4075

Bloem, Max: 7260

Bloemen, Elizabeth M: 1085, 3300

Blom, Jeanet W.: 7010

Bloom, Rachel F: 3200, 9040

Bloomberg, Mikaela: 4095

Bloss, Ellen: 1115, 1445, 9005

Blue, Colleen M: 7350, 7930

Blumen, Helena M: 8015

Boakye, Alfred: 4550, 8000, 9005

Boamah, Sheila: 2515

Boateng, Isaac Asirifi: 7340, 7395, 7455, 7920, 7935

Boateng, Josephine A: 1075, 7375, 8000

Boateng, Maame-Owusua: 7210

Bober, Timothy: 4305

Bobitt, Julie: 1315, 1500, 3045, 4390, 7185, 8015

Bock, Payton: 1520

Boddie, Stephanie C: 9105

Bodenheimer, Lisa: 7340, 7920

Bodner, Ehud: 3360, 8015

Boerner, Kathrin: 3080, 7200, 7475

Boeve, Angelica R: 1050, 2240

Bogaerts, Stefan: 7035

Bogdan, Ryan: 4040

Boger, Jennifer: 7205, 7915

Bogumil, Elizabeth: 3000

Bohall, Jeriel: 7205

Boisvert, Rachel: 7425

Bokun, Anna: 2060, 3265, 7475

Bolduc, Jessica J: 7225

Boliboun, Jimmie: 7485

Bolkan, Cory: 3295, 7175, 7920

Bollan, Josh: 1085

Bollens-Lund, Evan: 4430

Boller, Benjamin: 7200, 7325

Bolles, Leah: 8000

Bollin, Salli: 2485

Bolling, S. Alison: 7475

Boltz, Marie: 1310, 2180, 3035, 3220, 4380, 4480, 7465, 7500, 8010, 8015, 9075

Bolz, Fabio: 7040

Bonar, Erin E: 4390

Bond, Lynden: 7360

Bonds Johnson, Kalisha: 3375, 4160, 4485, 7330, 7350, 8015

Bonner, Alice: 1435, 4405

Bonnet, Kemberlee: 2255

Boockvar, Kenneth: 4445

Boon, Jeffrey T: 2045

Boot, Walter R: 1285, 3025, 3215, 3340, 4080, 4200, 4320, 4420, 7295, 8010, 8015, 9085

Booth, Richard: 1430

Booth, Sarah: 4295

Borden, Enid: 8010

Bordes, Piper J: 4395, 7450

Borges-Machado, Flavia: 7370

Borgogna, Joanna: 8010

Borgogna, Joanna Lynn: 3260

Borodovsky, Jacob: 4390

Borrero, Lisa: 2330, 2455

Borson, Soo: 7185, 9030

Boscardin, John: 7010, 7140, 7255

Bouchard, L.B.: 4035

Bouchard-Liporto, Madison: 1100

Boucher, Nathan A: 2090, 4210, 4565, 7075

Boudreau, Jacqueline: 2540, 7280, 7350

Boudreaux, Michael: 8005

Bouldin, Erin D: 4265, 8010

Bouranis, Nicole G: 7000

Bourassa, Kyle J: 2025

Boustani, Malaz: 2285, 2555, 3010, 7010

Boustani, Zayn: 7010, 7415

Bowblis, John R: 1150, 3280, 4260, 4365, 4440, 7250, 8010

Bowen, Holly J: 8010

Bowen, Lauren M: 1130, 2145

Bowen, Pamela G: 7245

Bower, Peter: 9085

Bowers, Katherine E: 8010

Bowland, Sharon E: 7110, 8005

Bowles, Kathryn H: 8000, 8005, 9135

Bowman, Brynn: 9145

Bowman, Grace: 7465

Box, Nicholas: 9070

Boxer, Rebecca S: 7085, 7910

Boyd, Cynthia: 7385

Boyina, Satvika: 8015

Boyle, Julia T: 2240

Bozaykut Eker, Perinur: 7315, 8005

Brach, Jennifer: 4525, 8005, 8010, 9095

Brackbill, Robert M: 7345

Brackpool, Kristina: 9105

Bradford, Ethan: 8015

Bradley, Dana B: 8000

Bradt, Joke: 8005

Brady, Debbie: 8015

Brady, McKayla: 1005

Brady, Samantha: 1405, 2050, 3250

Brandas, Benedetta: 7265

Brandis, Leah M: 4400

Brandon, Amanda: 4347

Brandt, Mark J: 3270

Brandt, Martina: 2345

Brandt, Nicole: 7290, 7495, 8005

Brangman, Sharon A: 4455

Branson, Brianna: 7185

Braswell, Angela M: 7340

Bratches, Reed: 8015

Brathwaite, Justin S: 1220

Braun, Robert T: 9090

Brauner, Rachel A: 8000

Braunstein, Joel B: 7010

Bray, Emily: 9005

Bray, Stephanie: 9020

Brazg, Noa: 2205

Brazier, Joan F: 7050, 8000

Brecht, Mary-Lynn: 1295

Breda, Kathleen M: 7485, 8000

Breder, Kelseanne: 3240, 4155

Breen, Elizabeth: 8015

Brehmer, Yvonne: 1240, 1480

Brekke, Bailee: 3125, 7540, 8005

Brennan, Caitlin: 7220

Brennan, Taylor: 3250, 7005

Brennan Ing, Mark: 1490, 2145, 2395, 7265

Brenowitz, Willa: 3050, 8000, 8005

Breton, Jordana: 2345, 7145, 7435

Breuner, Don: 7415

Brewer, Judson: 1160

Brewer, Robin N: 9070

Brewster, Glenna S: 4215, 4335, 7350

Brice, Kelly N.: 8000, 9015

Briceño, Emily: 1500, 2000, 3380, 4540, 8010

Bridgforth, James: 2515

Briggs, Anthony: 8005

Briggs, Emily: 3230, 8010

Brill, Seuli: 8005

Brines, Elizabeth: 8010

Bristol, Alycia: 1035, 1300

Britt, Katherine Carroll: 3025, 3260, 4225, 9010

Brittain, Katie: 9100

Britton, Karysa: 1045

Brock, Kaye: 7430

Brocksmith, Hannah: 7185

Brodaty, Henry: 4470

Brody, Abraham: 1270, 2380, 2440, 3205, 3375, 4015, 7185, 8010

Brody, Lilla: 7375

Broen, Tiana: 7020, 7195, 7900

Brohard, Cheryl: 4265

Bronas, Ulf G: 7020

Bronstein, Laura: 4185

Brooks, Ezra S: 8005

Brooks, Jada L: 1020

Brooks, Ron: 3170

Brooks, Sonya: 4250

Brooks Carthon, Margo A: 8005

Brothers, Ally: 2170, 7380

Brotman, Rebecca M: 8010, 9150

Brotman, Shari: 7065

Brousard, Jordan: 4415

Brown, Amy: 4230

Brown, Cameron: 4440

Brown, Candace: 4007, 4230

Brown, Casey K: 1275, 2025, 4515, 7290

Brown, Diane K: 4600

Brown, Karl J: 4445, 7410

Brown, Lana M: 7220, 7410

Brown, Lauren L: 4440, 8015

Brown, Mallory M: 7030

Brown, Maria T: 2405, 4455

Brown, Maya P: 7350

Brown, Monique J: 9025

Brown, Rebecca: 1220, 2245, 3025, 8015

Brown, Roger L: 8005

Brown, Sarah E: 8010, 9150

Brown, Susan L: 2295

Brown, Susan H: 7120, 7310

Brown, Sylvia: 4200

Brown, Taylor J: 7365, 7505

Browning, Wesley R: 4465

Bruce, Marino A: 3180

Bruni, Pietra T: 1050

Brunner, Olivia: 8005

Brunson, Amie: 8010

Brunswick, Chad A: 7190

Brunt, Christopher S: 4260

Brus, Anastasiia: 8015

Bryan, Emma G: 7350

Bryan, Joanna: 7440

Bryan, William E: 3065

Bryant, Ashley Leak: 4095

Bryant, Natasha: 7075, 7300

Bryer, Chase M: 1225

Buchanich, Jeanine M: 2200

Buchinger, Laura: 4490

Buchman, Aron: 7200

Buck, Harleah: 2070, 3355, 7225, 7335

Buckey, Jay C: 8015

Buckley, Nina: 2265

Buckley, Rachel F.: 9040

Buckwalter, Kathleen: 7005, 7305

Bucy, Taylor: 1010, 1485

Budhathoki, Chakra: 4385

Budinich, Marilu: 9035

Budney, Alan: 4390

Buedo, Paola: 7145

Buffalari, Deanne: 3150

Buffenstein, Shelley: 3267

Buitron, Daphne: 1055, 7255

Bulanda, Jennifer R: 7335

Bullich, Santiago: 7415

Bullock, Karen: 7290, 7500, 8010

Bülow, Per: 8005

Bülow, Pia: 8005

Bulut, Eyuphan: 4200

Bunker, Jennifer: 3280, 7185, 7485, 7930, 8005

Bunn, Melanie Gayle: 2475

Burack, Orah R: 7250, 7330

Burciu, Roxana: 7315

Burd, Christin E: 8010

Burd, Nicholas A: 8000

Burdick, Jennifer: 2355

Burdick, Katherine E: 7460

Burek, Melissa: 7370

Burgdorf, Julia G: 1270

Burgess, Elisabeth O: 4550

Burgess, Tanya L: 7470

Burghardt, Andrew: 1460

Burgos Zuniga, Claudia C: 7395

Burke, Robert E: 8015

Burk-Leaver, Erin Y: 1000, 9130

Burkley, Julie: 4545

Burman, Bonnie K: 2485

Burnes, David: 1085, 1215, 7180

Burnet, Evie N: 7200

Burnett, Jason: 1085, 4415

Burnett-Bowie, Sherri-Ann: 4460

Burnette, Denise: 7260

Burns, Adam: 1460

Burns, Edith: 2175, 7215, 7410, 8000, 9055

Burns, Hannah: 2085

Burns, Jeff: 7265

Burns, Katharine: 7460

Burns, Paige: 9095

Burns, Reilly M: 8015

Burns, Shane D: 4290

Burr, Jeffrey A: 3335, 7080, 7170, 7355, 7375

Burton, Lindsay: 7485

Burton, Lydia M: 8015, 9065

Bush, Adam: 8005

Bustillo, Antonio J: 8005

Buta, Brian: 2100, 3105

Butera, Katie A: 7140

Butt, Maayra I: 7375, 8005

Buxbaum, Channa: 7185

Buxton, Orfeu M: 2230, 3060, 7390, 7535, 8005

Buys, David: 8015

Bybee, Sara G: 4240, 8010, 8015

Byeon, Sukyung: 8010

Byers, Amy L: 7205, 7255

Bygrave, Desiree: 7465

Bylund, Carma: 7350

Bynum, Julie: 7120, 7250, 8005, 9035

Byon, Ha Do: 8010

Byrd, DeAnnah R: 1450

Byrd, Elizabeth: 7245, 7280

C

Cabrera, Nora Carolina: 8005

Cabrera, Ryan: 1305

Cabrera, Samir: 8010, 8015

Cabrero Castro, Jose E: 8015

Cacchione, Pamela Z: 3390, 8010

Cacciatore, Stefano: 8010

Cacciatore, Stefano: 8015

Caceres, Maria: 1160, 3355, 7015

Cachioni, Meire: 7370

Cacia, Ava Marie: 7150

Cadar, Andreia: 8000

Cadden, Margaret: 7415

Cadet, Tamara: 8010, 8015

Cadmus, Eniola O: 4565

Cagle, John: 1145, 7220, 7500, 9005

Cahill, Kevin E: 3310

Cai, Piaopiao: 4175, 7380, 7925

Cai, Shuang: 8010

Cai, Wenjie: 4060, 4540, 9040

Cai, Xinxin: 8010

Cai, Xiuting: 2305

Cai, Yihui: 9020

Cai, Yurun: 1255, 7500, 8010, 8015

Cal, Halvorsen: 1195

Calamia, Matthew R: 4395, 7450

Caldera, Selena: 2050, 3070

Calderon, Russell J: 4010, 7390, 9075

Caldwell, Clover: 7305

Caldwell, Joe: 3190, 8005

Calfee, Kris: 1475

Calhoun, Katie: 9110

Caljouw, Monique AA: 1010

Calkins, Maggie: 2080

Calkins, Margaret: 2080, 3035, 7465, 8010

Callahan, Kathryn E: 1110, 1255, 3160

Callaway, Teagan: 7170, 7220

Callow, Daniel D: 1510, 4375

Calloway, Haley: 4065

Calvani, Riccardo: 8010, 8015

Calver, Jen: 2465

Calvo, Esteban: 1425, 4325

Camacho, Cinthia: 4470, 7210

Camacho, David: 1055, 3265, 4150

Camacho, Leticia: 7495

Camacho, Paul B: 1255

Camacho-Rivera, Marlene: 8015

Cameron, Vanessa K: 7245

Cammack, Ralph: 7440

Cammer, Allison: 7205

Campbell, Claudia: 8010

Campbell, Don: 7545

Campbell, Emily: 8015

Campbell, Kenna: 8005, 8010

Campbell, Laura M: 8010

Campbell, Susanne: 7410

Campellone, Kenneth: 8005

Campitelli, Anthony: 7120

Camplain, Ricky: 8015

Campos, Blanca: 2555

Campos, Matthew: 7485

Can, Reyyan: 3360, 4270

Canell, Anastasia E: 7300

Canetto, Silvia Sara: 7255

Canham, Sarah L: 2515

Cannell, Michael B: 1085

Cannon, Melissa L: 7005

Cannon, Rachel: 2285, 7010, 7915

Cantrell, Randall: 4210

Cantu, Phillip A: 2060, 2345, 3265, 4540, 7465, 7475

Canzone, Mia R: 7405, 8015

Cao, Jiawei: 7010

Cao, Jing: 7040

Cao, Katy: 4245

Cao, Rui: 1405

Cao, Ying (Jessica): 4320

Cao, Yunhua: 4255

Capilupi, Michael: 7000

Cappon, Davide Balos: 7025, 7435

Caprio, Thomas V: 2205

Capuano, Ana: 8005

Carasso, Ella: 4185

Carbajal, Berta: 7305

Cardamone, Coleen: 7410

Cardenas, Deami: 8000

Cardenas, Janelle: 8015

Cardenas, Luisa: 3155

Carder, Paula: 3280, 4400, 7420, 8005, 8015

Cardona Puerta, Adela: 8015

Cardoso, Josue: 8000

Cardwell, Christina: 7300

Carey, Christopher: 3085, 7415

Carey, Cyriah: 7320

Carlile, Robert M: 7010

Carlin, Kevin: 2055

Carlozzi, Noelle: 4015

Carlson, Andrea C: 4460

Carlson, Barbara: 7370

Carlson, Bryant: 1490

Carlson, Michelle C: 2400, 8000, 8005

Carlson, Rose L: 4135

Carmichael, Pierre-Hugues: 8000

Carmiol-Rodriguez, Priscilla: 7100

Carnahan, Jennifer L: 1435, 4240, 8010

Carnahan, Ryan: 8010

Carneiro Marques, Fátima Cristina CC: 7300

Carney, Gemma M: 1365, 3140

Carney, Maria: 7410, 8000

Carniol, Renie R: 3000

Carpenter, Brian D: 1390, 7470, 8000, 8005, 8010

Carpenter, Christopher R: 8015

Carpenter, Joan G: 1145, 1300, 1435, 8010, 8015

Carpenter, Molly: 4405

Carpenter, Rebekah: 8015

Carpenter-Song, Elizabeth A: 2045

Carr, Alex: 7090

Carr, Brendan M: 7485

Carr, David B: 1355, 7505, 8015, 9015

Carr, Dawn Celeste: 1285, 2025, 2105, 3175, 3285, 4245, 4560, 8000, 8015

Carr, Deborah: 1140, 2020, 3195, 4000, 7055, 7290

Carrasco, Johanna: 9050

Carrillo, Anthony: 7245

Carrington, Crystal: 4075

Carroll, Judith: 1475, 8000

Carroll, Megan: 8000

Carroll-Sauer, Cathleen: 7225

Carstensen, Laura L: 1195, 1330, 3275, 4040, 8000, 8010

Carswell, Andrew: 4210

Carter, Aubrey: 8015

Carter, Chelsey R: 7210

Caruso, Rosario: 8015

Carvalheiro, Joana: 8000

Carvalho do Nascimento, Paulo Roberto: 7225, 7905

Carwile, Hope: 4475

Cary, Ann H: 8015

Cary Jr., Michael P: 3135, 3355

Casale, Sophia: 4130, 7200, 7225, 7260, 7905

Casanova, Giuliana: 7445

Case, Donna: 7295

Casey, James: 4515

Casey, Kamryn M: 7290

Caskie, Grace I L: 7005, 7455

Caspi, Avshalom: 7025, 7115

caspi, Eilon: 8010

Cassales Tosi, Fabiana: 7140

Cassario, Abigail: 3270

Cassidy, Jessica L: 1020

Cassidy, Weitman: 7050

Castañeda-Avila, Maira: 2555, 7255

Castellano, Michael: 8005

Castellanos, Stacy: 3290

Castells, Marianna: 8000

Castillo-Collazo, Delmarielinnette: 7200

Castle, John E: 1175

Castro, Hugo: 9035

Castro, Maria-Jose: 7285, 7925

Castro Sanchez, Barbara A: 8005

Catalano, Hannah: 7150

Cateau, Deke G: 1005, 4550

Cater, Lauren: 9015

Cauble, Christina: 3255

Caulfield, Maria C: 2335

Cavailles, Clemence: 4335

Cavalier, Alyssa: 8010

Cavanaugh, Connor: 7545

Cavanaugh, Sheamus: 2170

Cawthon, Peggy M: 1460, 7345, 8005

Ceïde, Mirnova E: 7525

Cellarius, Karen: 4400

Cenko, Erta: 1390

Cenzer, Irena K: 2245, 3050, 4070

Cepeda, Elizabeth: 8015

Ceria, Rachel: 8005

Cerino, Eric S: 1175, 2095, 2170, 2280, 4120, 7115, 7365, 8000

Cerino, Lauren: 3250, 4410

Cerniglia, Laura: 8005, 8010

Cernohous, Inessah: 8010

Cerqueira, Margarida: 7445

Cha, Kyeongin: 7325, 7425

Cha, Seung-Eun: 7335

Chaaya, Monique: 1495

Chachlani, Preeti: 7305

Chacon, Elyana: 7185

Chadwick, Darren: 8015

Chai, Hye Won: 1285, 2280, 7325

Chai, Shujun: 1235

Chak, Carly: 7005, 7935

Chakraborty, Shayok: 1285

Cham, Rakie: 8010

Chamberlin, Elizabeth S: 1305, 2365, 7230, 7280

Chamberlin, Keran W: 4375

Chan, Alex Chi-keung: 4180

Chan, Alex Kin-shing: 4180

Chan, Alex Siu Wing: 8005

Chan, Angelique Wei-Ming: 1205, 2435

Chan, Athena: 7185, 8000

Chan, Celine: 3155

Chan, Crystal Ying: 9085

Chan, Keith T: 8010, 8015

Chan, Kin Tung: 8000

Chan, Lily Man Lee: 7085

Chan, Wai Chi: 1235, 4455, 7255

Chan, Wai Sze: 1030

Chan, Wayne LS: 9020

Chan, Ya-Ning: 2255, 2475

Chandler, Blythe E: 1470

Chandler, Kelly D: 7335

Chandler, Rasheeta: 7015

Chandler, Zachary W: 8000

Chandler-Holtz, Lauren: 8005

Chandola, Tarani: 1030, 7360

Chang, Emiley: 7225, 7905

Chang, E-Shien: 2325, 3025

Chang, Jun Ha: 7465

Chang, Ling-Hui: 8010

Chang, Pei-Shiun: 7010, 7910

Chang, Rita: 8000

Chang, Timothy: 2555

Chang, Ya-Han: 1290, 4175, 7145

Chang, Yu-Ling: 4030

Chang, Yu-Ping: 7245, 7255, 7380

Chang-Lee, Shu-Nu: 7205

Chanti-Ketterl, Marianne: 1020

Chao, Pei-Shu: 4335, 7390

Chao, Shiau-Fang: 7165, 7370, 7465

Chao, Ying-Yu: 7255

Chapleau, Marianne: 7415

Chaplow, Zachary: 2220

Chapman, Brian G: 4220

Chapman, Elizabeth N: 2195

Chapman, Silvia: 1100, 7415

Chapman, Susan A: 1370, 3315

Chappell, Richard J: 1200

Charaf, Amani: 1135

Charamelo, Ana: 8015

Charankevich, Hanna: 2480

Charara, Rima: 8010

Chargo, Alexis N: 8005

Charles, Doreek: 4135

Charles, Susan T: 2095, 2280, 4120, 7460

Charlwood, Catherine: 2335

Charness, Neil: 4080, 4420, 8000, 8015, 9085

Chary, Anita: 1090

Chase, Cara: 2220

Chase, Gabrielle: 8015

Chasteen, Alison L. L: 1210, 7205

Chataut, Akankshya: 7390, 7475

Chatterjee, Sudeshna A: 4525

Chatterton, Hailey A: 7415

Chattopadhyay, Anurima: 1190

Chattopadhyay, Debaleena: 2040, 4590, 8010

Chau, Diane: 8005, 8010

Chau, Pui Hing: 4585

Chaudhary, Indra: 1500

Chaudhury, Habib: 2325, 3035

Chauhan, Shekhar: 2105, 3020, 4470, 4560

Chavarria, Stephanie: 8015

Chaverri Carvajal, Alexander: 7250

Chavez, Lorely A: 3355

Chavez Granados, Heily: 7205, 9120

Chee, Wonshik: 4065, 7305, 8005

Cheema, Zainab K: 8005

Chehab, Lora: 7405, 8010

Cheishvili, David: 8005

Chek, Carmen Jia-Wen: 8005

Chekuri, Srujana: 7160, 7390, 7475

Chel, Victor G: 4105

Chemalapatti, Kavya: 7195

Chemhaka, Garikayi: 7430

Chen, Amanda: 2465

Chen, Bangwei: 7410

Chen, Bangwei: 8015

Chen, Bin: 3090

Chen, Blythe: 7540

Chen, Chang-Chun: 8005

Chen, Chen: 2305

Chen, Chen: 8005, 8015

Chen, Chen: 8015

Chen, Chen X.: 7500

Chen, Chen-Fen: 7250

Chen, Ching: 8010

Chen, Chixiang: 3260, 4330, 7345, 7495, 9075

Chen, Chun-Liang: 8015

Chen, Cynthia: 4370, 7070

Chen, Cynthia: 8005

Chen, Diefei: 3380, 9045

Chen, Duan-Rung: 7220

Chen, Enna Y: 1195, 3275, 4085, 8000, 8010

Chen, Eric: 8015

Chen, Feng: 8015

Chen, Gong: 4255, 7300, 7430

Chen, Haiyi: 1235

Chen, Haiying: 1075, 1455, 2470

Chen, Hao-Min: 2300

Chen, Honglei: 1500, 7345

Chen, Honglin: 8000

Chen, Hongwei: 9150

Chen, Irene: 7440

Chen, Jen-Hao: 1490, 7245

Chen, Jenny: 9115

Chen, Jiachen: 8005

Chen, Jiawei: 7025

Chen, Jiayu: 7015

Chen, Jie: 7010

Chen, Jie: 7415, 7485

Chen, Jing: 1220, 1245, 4055, 7095, 7130, 7915

Chen, Jingsha: 1155

Chen, Kay: 1400

Chen, Keer: 4170

Chen, Kevin: 7255

Chen, Kuan-Hua: 4085, 4515, 7290

Chen, Lei: 2260, 4260, 8010

Chen, Li: 4335

Chen, Li-Mei: 7300

Chen, Lin: 7090

Chen, Lin Yee: 4375

Chen, Lingjun: 4060, 7225

Chen, Lingming: 8000, 8015

Chen, Liu: 2360, 3090, 7325, 7415, 8000

Chen, Liying: 7150

Chen, Lucia: 8010

Chen, Natalie: 7185

Chen, Qi: 2540, 8005

Chen, Qiaoxi: 1340

Chen, Ruijia: 3050, 4070, 4280, 7010

Chen, Shiyu: 4105

Chen, Shuo: 3260, 9075

Chen, Shyh-Huei: 2470

Chen, Si: 7420

Chen, Sinan: 7140

Chen, Sinuo: 4255

Chen, Stephanie Yu-Ching: 7410, 7935

Chen, Stephen: 1090

Chen, Tuo Yu: 2230, 7390

Chen, Wei-Ti: 1490, 7245

Chen, Wenjie: 8005

Chen, Xi: 1135

Chen, Xi: 1160, 1180, 2105

Chen, Xi: 3125

Chen, Xi: 3200, 7240, 8015, 9010, 9140

Chen, Xiaodong: 7225, 7905

Chen, Xiaowen: 2100

Chen, Xiayu S: 4565, 7210, 7295

Chen, Xiecheng: 9000, 9085, 9135, 9145

Chen, Xindi: 7530

Chen, Xingdong: 7320

Chen, Xinyi: 1240

Chen, Xuewei: 2290

Chen, Xuhui: 7190

Chen, Ya-Mei: 7140, 7370, 8010

Chen, Yanjun: 1200

Chen, Yaofeng: 8005

Chen, Yasheng: 1355

Chen, Ye: 8015, 9155

CHEN, Yilin: 4455

Chen, Yi-Ling: 7020

Chen, Yineng: 7445

Chen, Yingtong: 8000

Chen, Yu-Chih: 7070, 7365, 7405

Chen, Yu-Jen: 8005

Chen, Yuming: 2545, 7320, 7520

Chen, Yuxuan: 1275

Chen, Zhaojing: 7305

Chen, Zhe: 7050

Chen, Zhirui: 3305, 4530

Chen, Zhuolin: 2455

Chen, Zi: 4585

Chen, Zihan: 7050

Chen-Edinboro, Lenis P: 4070, 7150

Cheng, Bei: 4370, 7310

Cheng, Greta Jianjia: 2200, 9110

Cheng, Hongtao: 1245

Cheng, Huai Y: 2195, 3065

Cheng, Huai: 3065

Cheng, Huai Y: 7455

Cheng, Jie: 8015

Cheng, Joyce: 9115

Cheng, Kent Jason: 3195

Cheng, Kimberly: 7185

Cheng, Mengling: 2120

Cheng, Philip: 7390

Cheng, Qiqi: 1205

Cheng, Qiyuan: 8005

Cheng, Wan-Ju: 7020

Cheng, Yu-Shan: 8005

Chereches, Flavia: 1240

Chern, Andrew: 8015

Chernauskas, Tess M: 7395

Chernoff, Violet: 7415

Cherry, Barbara: 7145

Cherry, Katie E: 4395, 7450

Chervenick, Olivia N: 8005

Chess, Eric: 1215

Cheuk, Shui-kit: 7320, 7520

Cheung, David: 1385

Cheung, Ethan Siu Leung: 7260, 9115

Cheung, Monit: 7075

Chew, Alex: 1115

Chew, Justin: 7120

Chew, Keng Hao: 7235

Chi, Chih-Lin: 7305

Chi, Hua-Chin: 7305

Chi, Nai-Ching: 3220, 7265, 7305, 7500

Chia, Clement: 4370

chia, Hui Xiang: 3325

Chia, Jia Qian: 7120

Chiang, Hao-Ping: 7205

Chiaravalloti, Nancy: 8015

Chidebe, Runcie CW: 2220

Chie, Wei-Chu: 1365

Chih-Liang, Wang: 7340, 7470

Chikkala, Ashley: 4570, 8005

Child, Shaye: 7350

Chilton, Patricia: 8005

Chin, Janecca A: 3050, 4085

Chin, Meejung: 7290

Ching, Isabel: 8010

Chiriboga, David A: 2385

Chiriboga, David A.: 7210, 7305

Chisholm, Latarsha: 2265, 8010

Chisolm, Cynthia: 8000

Chiu, Wan-Yu: 7220, 7330, 8010

Chiu, Yu-Wen: 2300

Cho, Allison: 7000

Cho, Donghyuck: 7305

Cho, Emma: 7290

Cho, Euna: 4345

Cho, Eunhee: 7185, 7295, 7330, 7525, 8010

Cho, Haa-young: 3045

Cho, Hannah: 7295

Cho, Hyeonji: 4315

Cho, Hyunkeun: 2135

Cho, Isu: 7200, 7380

Cho, Jamie: 8015

Cho, Jinmyoung: 3255, 7475, 8000, 8010

Cho, Ji-Young: 1345

Cho, Joonyoung: 8005

Cho, Jungwon: 7185, 7295, 7330, 8010

Cho, Kelly: 8010

Cho, Kyoungmin: 2230, 7365

Cho, KyungAh: 7220

Cho, Sangyong: 8005

Cho, Seungjong: 3245, 4580, 7415, 7475

Cho, Sung Eun: 2275

Cho, Sung-il: 7090

Cho, Tsai-Chin: 1495, 2050, 4060, 4540

Cho, Yein: 3150

Cho, Yesang: 7295

Cho, Youngmin: 2255, 4385, 7390, 9075

Choate, Ashley: 1470

Chodos, Anna H: 4160

Chodosh, Joshua: 2045, 9030, 9045

Choi, Abigail: 7085

Choi, Bokyoung: 7005, 7135

Choi, Byeong Yeob: 3355

Choi, Edmond Pui Hang: 4585, 7085

Choi, Eun Young: 2005, 2250, 2275, 2345

Choi, Eunhee: 7005

Choi, George: 8015

Choi, Ha Young: 3230, 4320, 7295

Choi, HeeSun: 4590

Choi, HwaJung: 1375, 2050, 3010, 3230

Choi, Hwan: 7495

Choi, Hyein: 9125

Choi, Hyojin: 3135, 7395

Choi, Ian: 7085

Choi, Jean: 7200

Choi, Jeeyae: 7400

Choi, Jennifer L: 8005

Choi, Jeongeun: 7060

Choi, Jihee: 7005, 7135

Choi, JiYeon: 2210, 7055, 7300, 8015

Choi, Moon: 3025, 3075, 3340, 4420, 8000

Choi, Namkee G: 2310, 4300, 4415, 4530, 7020, 7155, 7255, 7395, 7465

Choi, Narae: 4180

Choi, Sangyong: 8000

Choi, Seongmi: 2210, 7055, 7300, 8015

Choi, Seo-Yun: 7210, 7390

Choi, Seung: 4015

Choi, Seung-won E: 3020

Choi, Seung-won Emily: 3020

Choi, Shinae L: 1140, 1365

Choi, Suhyeon: 7540

Choi, Yeon Jin: 7035, 7450, 7475

Choi, Yong Kyung: 2375, 3205, 3215, 4305, 7225, 7515

Choi, Yoonseok: 4270, 4490, 7390, 8010

Chon, Doukyoung: 8010

Chon, Yongho: 8015

Chopik, William J: 3270

Choquette, Elise: 1020

Chou, Kee-Lee: 4300, 7395

Chou, Lin-Na: 2190, 4580, 8015

Chou, Wan-Yun: 1290, 8010

Choudhry, Niteesh: 3390

Choula, Rita: 3070, 4100

Chow, Christine: 3340

Chow, Julian Chun-Chung: 2540

Chow, Stephanie W: 4425, 7455

Chowdhury, Nahian: 8000

Choy, Jacky C. P.: 2135, 8015

Choy-Yung, Rebecca: 9085

Christensen, Kaare: 7345, 8005

Christensen, Leah: 2365

Christofi, Sofia: 7115

Chrzanowski, Brandi: 7225, 7340, 7920

Chrzanowski, Lauren: 7305, 7400, 7415, 7900

Chu, Charlene: 7370

Chu, Chenyu: 4275

Chu, Jun: 2305, 7095, 9140

Chu, Ruotong: 4330

Chu, Sang Hui: 7375

Chua, Grace: 1205

Chua, Shiyun: 7120

Chuah, Joshua: 4060, 8000

Chudasama, Darshan: 2190

Chui, Cheryl Hiu-Kwan: 1135

Chukwu, Ngozi E: 1365

Chukwuemeka, Nkechi A: 8015

Chukwuorji, JohnBosco Chika: 2220, 7405

Chulak, Jamy: 1390

Chun, Gloria: 2350

Chun, Yung: 8010

Chung, Daum: 4335, 7330

Chung, Edwin Ka Hung: 2350, 8005

Chung, Hyeran: 1250, 2060

Chung, Jane: 3260, 4200, 8015

Chung, Jayer: 7300

Chung, Ji Won: 4475

Chung, Joohyun: 2465, 4135, 7220, 7370, 7910

Chung, Ka Hung Edwin: 8015

Chung, Paul J: 7510

Chung, Sandrine Man-chi: 1385

Chung, Soondool: 7210, 7290, 7305

Chung, Sulki: 3020

Chunga, Richard E: 7305

Chung-Bridges, Katherine: 8000, 8015

Chupak, Anna L: 9025

Churchill, Emma G: 7375

Chwa, Amir: 4370

Chyn, Eric T. Y.: 8010

Cicero, Ethan: 1000, 2145, 7110

Cichy, Kelly E: 7115, 7335

Cidav, Tom: 1055, 4450

Cieslik, Katarzyna A: 2125

Cifuentes, Christina: 1275

Cigliana, Jill: 7205, 7300, 7910

Cilluffo, Silvia: 7335, 8015

Cimarolli, Verena: 7075, 7280, 7360, 7475

Ciolfi, Mary Lou: 1115, 4480, 7170

Cipolli, Gabriela C: 7090

Cipriano, Andrea: 4107

Cisek, Edward: 1160, 1225

Cizginer, Sevdenur: 8000

Cizik, Amy M: 1025

Clair, Catherine A: 2030, 7500

Clancy, Erin T: 4050

Clancy, Margaret: 1070, 7120

Clapp, Jonathan D: 7420

Clare, Linda: 2335

Clark, Alexandra L: 4570, 8005

Clark, David J: 4525

Clark, Jeff: 7300

Clark, Kirsty A: 8005

Clark, Lindsay R: 1200, 8015

Clark, Maria F: 7200

Clark, Maria: 8010, 8015

Clark, Melissa: 1465, 2160, 7220

Clark, Phillip G: 2205

Clarke, Mary F: 8010, 8015

Clarke, Philippa: 1075, 1370, 1375, 4440, 7465

Clauw, Daniel: 4450

Clay, Olivio J: 3370, 8015, 9125

Clayton, Jordana L: 8015

Clayton, Tanya: 7220

Clayton-Jones, Mary (Kate) C: 1020

Clem, Sarah: 7085

Clements, Nia: 1040

Clemings, Kelly A: 7405

Clemons, Cailyn: 2005, 7535, 7900

Clervil, Stephanie: 8010

Clevenger, Carolyn K: 1275, 2160, 7185, 8010, 9025

Clish, Clary: 8005, 9155

Cloney, Nicholas D: 9105

Cloughesy, Jonathan N: 8010

Clouse, Kate: 3355

Cloyes, Kristin: 2030, 2255

Clutton, Jonathan: 7265

Coates, Martha: 8005

Coats, Heather: 7085, 7910

Coats, Jacquelyn: 8000

Cobb, Ryon J: 4125

Cobos, kimberly: 8005

Coccia, Kathryn W: 8005

Cochran, Jeremy: 8005

Cochrane, Barbara: 8010

Coco, Laura: 7535

Coe, Antoinette B: 7205

Coen, Paul M: 4275, 8000

Cohen, Andrew B: 8000, 8005, 9000

Cohen, Ann: 1255, 2200, 8015

Cohen, Cari: 7330, 7390

Cohen, Deborah: 4470, 7210

Cohen, Inessa: 7090, 8015, 9140

Cohen, Jessica: 7215

Cohen, Marc: 1130, 1150, 3155, 7085, 7250, 7255

Cohen, Olivia: 1160

Cohen, Steven A: 2255

Cohen, Yafit: 4195

Cohen, Yarin: 1090, 2540, 4315

Cohen-Fridel, Sara: 8015

Cohen-Mansfield, Jiska: 3130

Cohn-Schwartz, Ella: 2370, 2390, 7110

Coker, Karen: 7005

Colón-Emeric, Cathleen: 3135

Colón-Semenza, Cristina: 2380

Colcombe, Stan J: 8015

Cole, Connie: 1300

Cole, Evan: 8000

Cole, Marilyn B: 7275

Cole, Steven: 7115

Coleman, Carissa: 1150, 1515, 2225, 7010, 7250

Coleman, Leonardo: 9090

Coleman, Max: 9030

Coleman, Rachel L: 1115, 7170, 8005, 8010, 8015

Coley, Rebekah L: 3305, 4530

Colicino, Elena: 8010

Collie, Angel: 1285

Collinge, Charles W: 8015

Collins, Amber N: 1060

Collins, Joy: 8005

Collins, Kristin: 7340

Collins, Lauren F: 9080

Collins-Pisano, Caroline: 4575, 7395, 8005

Colman, Allison: 4020

Colon-Emeric, Cathleen: 1435, 3065, 7385

Colon-Vargas, Monica: 3370

Colson-Fearon, Brionna: 4530

Colvin, Calvin: 8000

Coman, Emil: 8010

Combs, Collean: 1165

Comer, Russ: 7280

Compernolle, Nell: 1115, 1445, 4085, 7380, 9005

Conant, Rachel: 4160

Conard, Ryan: 8015

Conde Freniche, Alberto: 8010

Condon, Ashely: 4105

Cong, Zhen: 1400, 2275

Conklin, Jamie: 8010

Connell, Elizabeth H: 2310

Connell, James: 1335

Connelly, Denise: 1430

Connolly, Marie-Therese: 1085

CONNOR, KATIE M: 1050

Connor Eruchalu, Jade: 7205

Conroy, David E: 8000

Constantino, Gailan: 8005

Contento, Angela: 3165

Convoy, Dana: 4075

Cook, Diane: 4045

Cooke, Alicia K: 3345

Cooke, Eric: 7370

Cooley, Marion: 8010

Coon, David W: 1110, 7305

Cooper, Jasmine: 9115

Cooper, Lujira: 2525

Cooper, R. Amanda: 7185, 8010, 8015

Cooper, Shelby J: 7250

Cooper, Zara: 8005, 9150

Coppola, Andrea: 1295

Coppotelli, Giuseppe: 1520

Corazzini, Kirsten: 1395, 1400, 2150

Corbisiero, Kyra: 8010

Cordero, Luis J: 7370

Cordova, Lourdes: 7305

Coresh, Josef: 1155, 1200, 2535, 3105, 4385, 7545

Corley, Robin P: 4110

Corley, Samantha S: 7255

Corlin, Laura: 7315

Corman, Samantha C: 7545

Cornelison, Laci: 4405

Cornelius, Kala: 3005, 4350

Cornish, Emily J: 8005

Cornman, Jennifer C: 2375

Coromac-Medrano, Juliamaria: 7200

Correia, Stephen: 8015

Cortes, Constanza: 3397

Cortes, Tara A: 2015

Cosentino, Stephanie: 1100, 7095, 7415

Costa, Amy N: 1475, 4230

Costa, Carolina L.: 8005, 8010

Costanza, Katelyn: 3295

Costello, Erica: 3350

Costello, Janina: 7150

Cotten, Shelia R: 3340

Cotter, Katherine N: 8000

Cotton, Quinton D: 3375

Cotton, Sam: 1060, 2465

Cottrell-Daniels, Cherell: 8000, 8015

Couch, Elyse: 4330, 7185, 7415

Coughlin, Joseph F: 1405, 3250, 4410

Coulourides Kogan, Alexis: 1215

Coulter-Daly, Isabel: 8010

Covarubbias, Anthony J: 2277

Covinsky, Kenneth: 2245, 9090

Cowan, Ronald L: 2045

Cowan-Pyle, Alexandra E: 7480

Cowley, Martha: 1115

Cox, Brian S: 4475

Cox, Carole: 3055

Cox, Mary A: 8005, 8015

Cox, Sarah: 1085, 3300

Coyle, Caitlin: 1410, 2185, 3000, 3080, 3170, 4130, 4545, 7030

Coyne, Danialle: 4425, 7455

Coyne, Riley: 1215, 9105

Craig, Taylor: 7420

Crainiceanu, Ciprian: 4375

Cram, Peter: 7305

Cram, Peter: 8010

Cramer, Candace L: 4545

Cramm, Jane: 7260

Crandall, Jill: 1455

Crane, Paul K: 3380

Crank, Peter J: 3235

Cranley, Lisa: 3040, 9135

Crawford, James M: 8000

Crawford, Kiersten: 2140

Crawford, Stirling: 8010, 8015

Cray, Hailey V: 8000

Creath, Robert: 8005

Crespo, Michael: 7475

Crews, Deidra C: 3345

Crimmins, Eileen: 2137, 2250, 3127, 7345

Crittenden, Jennifer: 1115, 7170, 8005, 8010, 9125

Crivelli, Lucía: 8015

Crocker, Andrew B: 8000

Crocker, Jillian: 7005, 7110

Croff, Raina L: 1280, 7475

Cross, Dori: 1010

Crowder, Hannah: 7530

Crowder, Victoria: 2255

Crowe, Michael: 2030, 3370, 4030, 4140, 4560, 4570, 7350, 9125

Cruikshank, Kirsten: 4045

Cruise, Cathy: 1305, 7280

Crum, Michelle: 2045

Cruz, Daniel S: 7485

Cruz-Carrillo, Yesenia: 4290

Crystal, Stephen: 2020

Cserepy, Araya: 8000

Cubbin, Catherine: 4530

Cudjoe, Thomas: 1360, 2190, 7330

Cuevas, Jadira: 7095

Cuff, Patricia A: 7075

Cui, Linna: 7480

Cui, Michael: 7485

cui, xianmei: 3075

Cui, Xirong: 7425, 7430

Cullen, Katie: 7275

Culligan, Melissa J: 8010

Culver, Clare: 8010, 8015

Cummings, Steve: 3170

Cummings, Steven R: 1460, 4275, 4495

Cuozzo, Juliana: 3070

Curl, Angela L: 7335

Curran, Sean P: 1520, 2137, 3120, 4227

Curriero, Samantha: 4445

Curtis, Ashley F: 1475, 4230, 7390

Curtis, David S.: 7260

Curtis, Lesley H: 8000

Curyto, Kimberly: 1060

Cushing, Cynthia: 8005

Cutforth, Asia: 1215

Cutforth, Asia: 9105

Cutrona, Carolyn: 4195

Cutty, Maxwell: 8005

Czaja, Sara: 1170, 2325, 3025, 4080, 4525, 8000, 8015, 9085

Czarny, Anna: 9020

Czech, Stephanie J: 8015

Czeisler, Charles A: 7390

D

Da, Wendi: 4070, 4505

da Rosa, Grace: 1065

Dabelea, Dana: 1455

Dabelko-Schoeny, Holly I: 3125, 3305, 4130

Dacey, Catherine: 7545

Dacks, Penny A: 7185

Daddato, Andrea E: 4090, 7085, 7910

Dadgostar, Porooshat: 3115

Dadson, Deborah A: 8000

Dagan Yarkoni, Ayelet: 1515

Dagen, Nicole: 7220

Dagne, Mahederemariam: 4190, 9065

Dahal, Arati: 1270

Dahal, Poshan: 7380

Dahal, Roshani: 3095

Dahl Aslan, Anna: 8010

Dahle, Cheryl L: 8005, 8010

Dahms, Eric: 7510

Dai, Sherwin: 1520, 7480

Dai, Shuting: 9060

Daks, Jennifer: 2085

Dalal, Taykhoom: 3090

D’Alesio, Mark: 4435

Dalgård, Christine: 7345

Daly, Rebecca: 7050, 7375

D’Ambrosio, Conchita: 1210

D’Ambrosio, Joseph: 2110

D’Ambrosio, Lisa: 3250, 4410, 7005, 8005

Damman, Marleen: 2350

Damoiseaux, Jessica S: 1255

Damul, Benmun: 7055

Danaei, Goodarz: 7010

Daneshvar, Nina: 1235

Dang, Kristina Van: 2250

Dang, Linh: 7335

Dang, Stuti: 8010, 8015

D’Angelo, Hannah: 7470

Daniel, Jacquelin: 1260

Daniyalo, Kadir Abdul: 8000

Dannefer, Dale: 2020

Danquah, Ahmed: 4465

Danquah, Ahmed: 8000, 8015

D’Antonio, Patricia M: 2215, 2355, 3210, 4555

Dark-Freudeman, Alissa: 1390

Das, Malay: 8000

Das, Mohana: 7465

Das, Sayani: 3305

Das, Stuti: 8015

Das, Sudeshna: 7415, 9040

Das, Utpol: 8000, 8015

Das Gupta, Debasree: 1355, 4030, 4440, 4560

Dasanayake, Thakshila: 8000

Dasariraju, Satvik: 8000

Dasgupta, Abhirupa: 4150

Dash, Chiranjeev: 7190

Dassel, Kara B: 1380, 4555, 7405, 8010, 8015

Dastgheyb, Raha: 1155, 7245

Dastidar, Bonnie: 2200, 9110

Daudi, Leonarda: 9130

Daugherty, Ana M: 8005

Daugherty, Ana: 8010

Dauphinee, Dale: 8000

Daus, Marguerite: 7485, 7930

Davey, Adam: 2080

David, Daniel: 2440, 4155, 7250

Davidson, H Edward: 7305

Davidson, Patricia M: 7380, 7430, 7495

Davidson, Sylvia: 7340

Davie, Laura: 1395, 2150

Davies, Casey: 2030

Davies, Tracy: 8000

Davies-Teye, Bernard: 2125

Davila, Ana Luisa: 3370

Davila, Carine: 9120

Davila, Heather: 1305, 2085, 8005

Davila-Roman, Ana Luisa: 3370

Davis, Brian: 8010

Davis, Carter H: 7215

Davis, Elizabeth: 9145

Davis, Elizabeth: 9150

Davis, Henry J: 7355

Davis, Isabelle: 2265

Davis, Jennifer C.: 7225, 7905

Davis, Jonathan: 1500

Davis, Matthew: 8005

Davis, Rachel: 2400

Davis, Rachel: 8010

Davis, Robert: 8000

Davis, Scott: 7225

Davis-Hearn, Chelsea N: 7300

Davitt, Joan: 1145, 7085, 7220

Davlyatov, Ganisher: 3365

Davoudi, Anis: 2100, 2480, 4375

Davy, Brenda: 8010

Davy, Kevin P: 8010

Dawe, Connor: 4500

Dawes, Connor: 4500

Dawson, Walter D: 4400, 7010

Dayanandaswamy, Chirantana: 9015

Daylor, Jayne: 7410

Dayrit, Jessika: 8005

De Anda-Duran, Ileana: 3380

de Boer, Bram: 3035

De Fries, Carson M: 1215, 1240, 4040

De Jong, Marla: 4555

De Jonge, Karl E: 3340

de Klerk-Rubin, Vicki: 7215

De Main, Atami: 1055, 1490, 7255, 8015

De Maria, Maddalena: 7335

de Paula Couto, M. Clara: 1210, 2370, 8015

de Rosa, Cristina: 7335

De Sanctis, Pierfilippo: 7025

De Vilmorin, Charles: 8015

De Vito, Alyssa: 2000, 7290, 7305, 8000

De Wit, Liselotte: 8005

de Wit, Sanne: 4580

Deal, Jennifer: 1075, 1200, 2535, 7330, 8010

Deardorff, W. James: 7140

DeBlois, Jacob P: 2085

Debras, Charlotte: 8010

DeCarli, Charles: 2040, 8010

Decker, Autumn: 3295, 4565

Decker, Chase: 9080

Decker, Veronica B: 1055, 7225, 8005

Declercq, Anja: 1060

DeConne, Theodore: 8000

Deek, Rebecca A: 8000

Deep, Gagan: 8015

Defillo Draiby, Julio: 7000

DeForge, Christine: 7305

Degenholtz, Howard B: 1385, 4100, 4260, 7370, 8005, 8010

DeGennaro, Athena P: 1360

DeGennaro, Jennifer: 7340, 7410

DeGloria, Michael: 8000

Dehghanian, Marjan: 2555

Deichert, Jerome A: 4550

DeJohn, Amber: 4245

Del Bene, Victor A: 4570

Del Bosque, Jennifer: 8015

Del Carpio, Karina: 2275

DeLany, James P: 2470

DeLawter, Alana: 8000

Delemeester, Natalie K: 7540

Delgadillo, Jeremy D: 9125

Delgado, Carolina: 8015

Delgado, Ferdinand: 8010

Delgado, Kimberly: 8000

DeLiema, Marguerite: 1010, 2115, 7405

Deliu, Zane: 7145

Delizia, Morgan R: 8000

Delmelle, Eric: 9105

DeLuca, John: 8015

Demchak, Sydney M: 8005

Demers, Camille: 7065

Demesi, Joy C: 1430

Demeter, Naor: 4195

Demetres, Michelle: 1505, 7105

Demir, Gokhan: 3065

Demir, Murat M: 4350

Demiris, George: 3390, 4200, 7205, 7220, 7260, 7290, 7295, 8015

DeMott, Andrew: 3045

Demuth, Ilja: 4050, 4490

Deng, Amy: 8015

Deng, Qiyu: 7395

Deng, Yanran: 2545, 7320, 7520

Deng, Zhirui: 8000, 9050

Denis Oliva, Francyess: 7170, 7220

Dennis, Elizabeth A: 2395, 7130, 7455

Dennis, Paul: 4430

Denny, Bryan T.: 8000

Denny, Sharon S: 7185

Denson, Kathryn: 7455

Denson, Steven: 7455

Dent, Kallisse R: 7255

Dentry, Tyler: 8015

DePaolis, Fernando: 9075

Depp, Colin: 7050

Derby, Carol A: 1110, 4310, 7535, 8005

Derivaux, Mattie: 2535

Deroo, Rebekah: 8000

Derricks, Veronica: 3085

Dertinger, Melinda: 4045

Deschodt, Mieke: 1060, 8010

Deshaies, Marie-Hélène: 7065

Deshmane, Aarohi: 7365

Deshmukh, Madhyami: 7470

DeSilva, Mary: 8015

Desir, Marianne: 2540, 7280, 7350, 8010, 8015

Desjardins, Michael R: 2400

Desmond, Grace: 8000

DeSouza, Dannah D: 8005

Destrampe, Melissa A: 1130, 3155

Deutsch, Judith: 7140

Devenport, Winry F: 8005

Devlin, Kathryn: 7290

DeVone, Frank: 8005

DeVries, Marla: 8010

Dewey, Erica: 3150

DeWitt, Avery: 7210

Dey, Amit: 7315, 7480

Dey, Nutifafa E Y: 8000

Deyoung, Audrey: 4485

Dhakal, Samitinjaya: 7130

Dhakal, Usha: 1500, 3380

Dhammapeera, Phot: 7375

Dhamne, Sayali: 7120

Dhana, Klodian: 4570

Dhanavanthri Muralidhara, Sneha: 4230

Dharia, Aastha: 1370, 8000

Dhingra, Lara: 7305

Dhingra, Radha: 4095, 7520, 8000

di, liu: 7190

Di Florio, Paola: 9020

Di Gessa, Giorgio: 3145

Di Rosa, Mirko: 7070

Diallo, Ana F: 1260, 7095

Dian, Larry: 7225, 7905

Diaz, Aymara: 8010

Diaz, Vivian: 8015

Diaz Nelson, Michele: 7000

Diaz-Asper, Catherine: 4190

Diaz-Santos, Mirella: 2555

Diaz-Valdes, Antonia: 1425, 4325

DiBacco, Grace M: 8015

DiCarlo, Nicholas: 1420

DiCesare, Elyse: 1340

Dicianno, Brad: 2375

Dicken, Megan: 8005

Dickerson, John: 8005

Dickson, Chelsea: 8005, 8010

Dickson, Daniel: 7065

Dictus, Cassandra: 2255, 4095, 4425, 4565

Dieciuc, Michael A: 1285

Diefenbach, Michael A: 2175, 8000

Diehl, Christin: 7515, 7930

Diehl, Manfred: 8015

Dieppa Colo, Emily: 1005

Dierckx de Casterlé, Bernadette: 8010

Diergaarde, Brenda: 8000

Dietz, Anna: 8000

DiFiglia, Stephanie: 2030, 4140

DiFranco, Lacey: 8000, 9010

Dikhtyar, Oksana: 7420

Dikken, Jeroen: 3080

DiLalla, Linda: 1050

Dill, Janette: 2260, 3075

Dillon, Ellis: 1315, 2185, 4135, 7360, 8005

Dillon, Kayla: 1035

Dillon, Simon T: 2505

DiMaria-Ghalili, Rose Ann: 7360, 8005

Diminich, Erica D: 9025

Dinata, Erna: 7375

Dindo, Lilian: 7030

Ding, Dan: 4305

Ding, Jie: 4385, 7545

Ding, Jinfeng: 8015

Ding, Jingzhong: 7480

Ding, Jingzhong: 8000

Ding, Kedong: 1140, 3245

Ding, Linlin: 4230

Ding, Margaret: 1305, 3315, 4205, 7270

Ding, Ruoxi: 4055

Ding, Weiyang: 3205

Diniz, Amanda: 7320

Diniz, Breno S: 4495

Diniz e Silva, Bruno Vinícius: 8000

Dinman, Michele: 8000, 8010

Dino, Michael: 3030, 4450, 7160, 7225, 8000, 8010

Dinov, Ivo: 1510

Dionne, Laura: 1465, 8015

Diunugala, Sithara D: 8000

Dixon, Steve: 8010

Djoroeva, Aydana: 7545

Do, Hien: 2275

Do, Jonghwa: 7055

Doan, Lan N: 3155

Dobbs, Debra: 2490, 3335, 7040, 7220, 7340, 7395, 7420, 7515, 7935

Dobrayel, Marie: 9055

Dodge, Hiroko H: 1185, 2360, 3090, 7325, 7415, 8000

Dodge, Shana G: 1015, 7185

Doering, Sabrina: 4010

Dogan, Anil Sukru: 7315

Dogari, bolu: 3230

Dohan, Daniel: 7185

Doherty, Fiona C: 3125

Dolan, Hanne R: 1110

Dolansky, Mary A: 1485, 7410

Dolberg, Pnina: 7450

Dold, Anna: 7265

Dolenc Nott, Brooke: 7005

Dollar, Blythe: 7085, 7910

Dolma, Rinchen: 2220

Domenicali, Marco: 9020

Domingorena, Sofia: 7145

Domingues, Marisa: 7370

Dominguez, Ahria J: 7290

Domnick, Hallory A: 7410

Donaher, Brooke: 4175

Donahue, Marissa: 7305

Donega, Stefano: 8000

Donelle, Lorie: 1430

Dong, Fanghong: 7290

Dong, Wuyi: 3320, 7200, 7435, 7530

Dong, Yan: 7145

Dong, Yanjun: 1105, 4395, 7295, 7485, 9030

Dong, Yu: 8010

Dong, Yue Coco: 8000, 9050

Dongmei, Zuo: 3285

Donnelly, Rachel: 9040

Donofry, Shannon D: 4585

Donohe, Krista: 1260

Donohoe, Maria: 2135, 4350

Donorfio, Laura KM: 4220, 7295

Donovan, Heidi: 2375

Donovan, Nancy J: 2310

Dorame, Ashley: 3330

Dorame, Breana: 8005

Doran, Kelly: 8005

Doran, Kevin: 7515

Dore, Emily: 1080

Doron, Malka: 4355, 7175

Dorris, Jennie L: 7490

Dos Santos, Luis Miguel: 1385

Dosa, David: 2500, 7255, 7410, 7485

Dougherty, Maura: 7125

Dougherty, Ryan J: 4375

Douglas, Natalie F: 1165, 1300

Douglas, Sara: 7220

Dove, Abigail: 4370

Dow, Patience M: 2465, 4010

Dowd, James J: 3140

Dowden, Lauren: 1015

Dowhower, Dan P: 7005

Downer, Brian: 1250, 2060, 3045, 7240, 7445, 8015, 9075

Doyle, Cassidy: 3185

Doyle, Margaret: 7530

Doyle, Margaret F: 8005

Dozier, Mary: 8015

Dozier, Stephen: 7190

Draica, Florin: 8010

Dremann, Andrea: 8000

Dresden, Scott M: 7485

Drewelies, Johanna: 4050

Drewelies, Johanna: 4490

Dreyfuss, Claire: 8005

Driver, Jane: 1470, 7220, 8000, 8010, 9080, 9090

Drolet, Marie-Josee: 2235

Drost, Jennifer: 4600, 7225

Druda, Ylenia: 9020

Dryden, Eileen: 2540, 3115, 7280, 7350

D’Souza, Deepak Cyril: 4390

D’Souza, Gypsyamber: 8015

Du, Chenguang: 8015

Du, Sixian: 7300, 7410, 7930

Du, Yao: 1235

Duan, Hongzhe: 7415

Duan, Liping: 4275

Duan, Yinfei: 2150, 7300

Duberstein, Paul R: 2320, 3205

Dublin, Sascha: 9090

Dubois, Lindsay: 3190

DuBois, Samantha L: 8005, 8015, 9025

DuBose, Logan: 9025

Dubowitz, Tamara: 2200, 9110

Duchateau, Francois Xavier: 8015

Duchesneau, Emilie D: 1255, 4065

Duchow, Olivia: 8000

Duchowny, Kate: 4440

Dueck, Kimberly: 3025

Duezel, Sandra: 4050

Duff, Kevin: 1185

Duffy, Erin L: 8010

Dufour, Alyssa: 7130

Dufour, Isabelle: 1080

Dufresne, Ruth: 8010, 8015

Dugan, Beth: 4220

Dugan, Elizabeth: 1075, 1335, 7130, 7170, 7185, 7200, 7210, 7225, 7245, 7255, 7260, 7300, 7305, 7375, 7530, 7905

Dulaney, Sarah OCS: 9000

Dumbadze, Sofio: 8010

Dumitrescu, Logan: 9030

DuMontier, Clark: 8000

Dumuid, Dorothea: 3235

Dunbar, Nora: 7365

Dunbar-Jacob, Jacqueline: 8010

Dunk, Michelle: 4370

Dunlap, Shawn: 2085

Dunn Lopez, Karen: 8010, 8015

Dupre, Mason L: 7520

Dupre, Matthew E: 4095, 8000, 8005

Duque, Gustavo: 1520, 3117, 4473

Durant, Raegan W: 4445

Durette, Kailey: 4500

Durham, Sonya: 8015

Duthie, Edmund H: 7455

Dutta, Chhanda: 4008

Dutta, Naibedya: 4275

Dutta, Raghbendra K: 8000

Dutton, Hillary: 2440, 8010

Dworak, Elizabeth M: 7435

Dwyer, Dan: 8000

Dykes, Patricia: 8015

Dys, Sarah: 3155, 3280

E

Eagle, Angelica: 4490

Earls, John C: 7015, 7190

Earp, Jacob E: 8000, 8010

East, Mary Ann: 8000

Eaton, Charles B: 8015

Eaton, Jacqueline: 2135, 2455, 4240, 4355, 4555, 7085, 7915

Eaton, William: 1510

Ebright-Jones, Blake D: 7460

Ebstein, Madison: 7290

Ebulum, Genevieve C: 7380

Echt, Katharina V: 2195

Echt, Katharina V: 7410

Economidou, Sofia: 8015

Edahiro, Ayako: 7440

Edelman, Linda: 2255

Edelstein, Offer E: 7185

Edlund, John E: 3150

Edmonds, Andrew: 2395

Edmonds, Leiha: 8005

Edwards, Madison: 8005

Edwards, Morgyn: 8005

Edwards, Robert: 7120

Edwards, Samuel: 7135

Edwin Sam Devahi, Adharsha Sam: 3090

Eerike, Madhavi: 7510

Efird-Green, Lea: 1435, 7440, 7915

Egbert, Anna R: 7020, 7265

Egge, Robert: 4160

Eglseer, Doris: 1310

Ehlman, MC: 7330

Ehrlich, Joshua R: 1075, 4065

Eich, Teal: 7395

Eichorn, Ann: 7215

Eilat, Shvat: 3080

Einstadter, Douglas: 8005

Eisenstein, Amy R: 2495, 4020

Ejaz, Farida: 1215, 8000

Ejem, Deborah: 4445

Ejikeme, Chidera A: 7390

Eka, Ni Gusti Ayu: 8000

Ekoh, Prince C: 1365

Ekwebelem, Maureen: 4225

El-Abbadi, Naglaa: 4440, 4460

El-Assad, Lauren M: 8015

Elayoubi, Joanne: 7350

Elbejjani, Martine: 1495

ELBOIM-GABYZON, MICHAL: 4355, 7175

Elder, Sadie Leder: 3150

Elfenbein, Pamela: 4355

Elftmann, Hanley: 7415

Elgayar, Sara: 3115

Eliassen, A. Heather: 9155

Eliazar-Macke, Nathaniel: 7350

Eliopoulos, Elaine M: 7270

Elisha, Victor: 8005, 8010

Eliyahou, Ayala: 1090

Elk, Ronit: 3165, 4240

Elkarif, Talia: 1330, 4235

El-Khatib, Lana: 2265

Ellington, Lee: 2135, 2255, 7085, 7915

Elliot, Amy: 4345

Elliot, Meghan R: 7050, 7460

Elliott, Annaliese: 7160

Elliott, Christian: 7305

Elliott, Emily M: 4395, 7450

Elliott, Maxwell: 7025

Elliott, Susan J: 3255

Ellis, Annika M: 8000

Ellis, Katrina: 3245

Ellis, Rebecca: 4390

Ellison, Teena: 8015

El-Makarim Aboueissa, Abou: 4105

Elman, Alyssa: 3300

Elmas, Cagri: 7470

Elmasri, Jeannine: 7015

Elmore, Catherine E: 2190

Elop, Alana: 9090

Elrod, Cathy S: 4190

Emami, Azita: 8000, 8005, 8015

Emans, Sinclair: 2417

Embon Magal, Shiri: 1110, 7040

Emerson, Amanda: 8010

Emery-Tiburcio, Erin E: 2110, 2240, 9140, 9145

Emma, Amanda: 2085

Emrani, Sheina: 4570, 7435

Emrick, Lauryn: 7500

Eng, Chloe: 3380

Engeland, Christopher G: 1445, 3060, 7325, 8005, 8010, 8015

Engelman, Michal: 3265, 4520, 4585, 7260, 7290, 8015

Engelmeier, Joey: 4305

English, Dan: 3255

English, Tammy: 1045, 3270, 4515

Engstrom, Gabriella: 8000, 8005, 8015

Enguidanos, Susan: 2265, 2390, 3300, 7220

Enriquez Hesles, Elisa: 7190

Enroth, Linda: 1080

Ensrud, Kristine: 1455, 1460

Epps, Fayron: 1135, 2420, 3375, 4335, 7015, 7475, 8010, 9025, 9130

Epstein, Mara: 9095

Epstein-Lubow, Gary: 2285

Eraslan, Emre: 8005

Ercan, Semra: 7265

Erices-Ocampo, Paulina: 8005

Erickson, Ashleigh: 8000

Erickson, Kirk: 4585, 7265

Erickson, Melissa L: 8005

Ericsson, Malin: 4030

Erkens, Petra: 7250

Erlenbach, Emily: 7265, 8000

Ermer, Ashley: 4360

Ernst, Jerome: 2395

Ernst, Joy: 7405

Ernsth-Bravell, Marie: 7325, 8005

Erodula, Nandini: 7265

Ersek, Mary: 1300

Ersek, Mary: 9005

Erving, Christy L: 1105, 4250

Escamilla, Valerie: 8010

Escareno, Juana: 3355

Eshraghi, Karen: 7305

Esiaka, Darlingtina: 2220, 2420, 3180, 7200, 8010, 8015

Espejo, Edie: 7010

Espeland, Mark A: 1455, 2470

Espinosa, Cisco: 1170

Espinoza, Kevin Enrique: 8000, 8015

Espinoza, Sara E: 1387, 2557, 4090

Esposito, Michael: 2005, 7465

Essen, Emma von: 1095

Esser, Karyn A: 8005

Estabrooks, Carole A: 2150

Estenson, Lilly: 1250, 8010

Estes, Kayley D: 7050

Estlein, Roi: 1125, 4185

Estrada, Jesus: 9055

Estrella, Mayra L: 8005

Etnier, Jennifer L: 7435, 8000, 8005, 8015, 9025, 9130

Ettman, Catherine: 3315

Etuka, Brandon Asika: 8015

Etzell, Martha: 2055, 8015

Etzkorn, Lacey H: 2100, 3105, 4375

Evangelista, Melanie: 2500

Evans, Abigail: 1145

Evans, Annalise: 7005

Evans, Daniel: 1460, 4495

Evans, Joni K: 1455

Evans, Katie: 4160

Evans, Michele: 7130

Evans, Taylor: 8015, 9050

Evans, Tracy L: 1035

Evans, William J: 1460

Evelyn-Gustave, Allyson: 8015

Everson-Rose, Susan A: 7425

Ewen, Heidi H: 8010

Eze, Ifeoma E: 8015

Ezeihuoma, Obinna: 7380

Ezeokonkwo, Francesca C: 8010

Ezeonu, Brian I: 9120

Ezzi, Zahra: 1310

F

Fabian, Tyfany: 7360

Fabisiak, James: 4305

Fabius, Chanee D: 1010, 2260, 3075, 7360

Facchetti, Teresa: 7095

Factora, Ronan: 7405

Fagan, Zoe: 8010

Fagundes, Christopher: 9015

Fagundes, Christopher P.: 8000

Fahey, Margaret C: 7180

Faieta, Julie: 4305

Falco, Vince: 8015

Falk, Derek: 2060

Falkstedt, Daniel: 1425

Falls, Katherine H: 1260, 7095

Falohun, Tokunbo: 9025

Falvey, Jason R: 7000, 7495, 8010, 9075, 9115

Falvo, Michael J: 8015

Falzarano, Francesca: 2390, 3025, 7040, 7475, 9040

Famula, Jessica: 2450, 4145

Fan, Alei: 7470

Fan, Bolin: 7365

Fan, Erica K: 2200

Fan, Mingming: 7295

Fan, Ning: 7220

Fan, Qiping: 9025

Fan, Xuehong: 4180

Fan, Zhiyu: 7340

Fanelli Kuczmarski, Marie: 7130

Fang, Hsin-Yu: 7315, 8005

Fang, James R: 4060, 7225

Fang, Mei L: 8005

Fang, Mei Lan: 1135, 1325, 8015

Fang, Siyue: 8005

Fang, Ya: 1235, 7010

Fanning, Jason: 2470, 8000

Fanzosa, Emily: 3115

Faran, Yifat: 2105

Faria, Frederico: 7225, 7905

Fariduddin, Osswaah: 8010, 8015

Farina, Mateo P: 1105, 2345, 3380, 7535, 9040

Farmand, Aida: 3310

Farmer, Heather R: 1415, 2345, 4095, 4125, 4360

Farmer, Justin: 8000

Farnham, Trish: 7075

Farrell, David: 4475

Farrell, Timothy W: 7030

Farsijani, Samaneh: 4295, 7345, 8000

Faruque, Irtiza S: 8000

Faselis, Charles: 9020

Fashaw-Walters, Shekinah: 1270, 8005

Fasolino, Tracy: 2075

Fassi, Janelle E: 4115, 7255

Fassnacht, Daniel: 4580

Fastame, Maria Chiara: 7265

Fastbom, Johan: 4370

Faul, Anna: 1060, 2110, 2465

Faul, Jessica: 7345

Faurot, Keturah R: 4535

Fauss, K. Melinda: 8005

Faust, Phyllis: 7415

Fauth, Elizabeth: 4085, 7215, 7305, 7330

Favero, Michelle: 3245

Fazeli, Pariya L: 4570, 7245, 9125

Fazio, Elena: 4008

Fdil, Mariam: 2235

Feaster, Daniel: 8010

Featherston, Kyle G: 7125

Feddoes, Daisy: 8005

Federman, Alex: 9080

Fedorov, Alex: 7015

Fedorovskyy, Maks: 8000

Fedus, Donna B: 2135

Feehan, Jack: 4473

Fein, Taylor: 8005

Feliciano, Leilani: 7020, 7195, 7900

Felix, Cynthia: 4030

Felson, David: 1070

Felt, John M: 1445

Felter, Cara: 7455

Felts, Adam: 3250, 4410

Fender, Chloe: 4347

Fenelon, Andrew: 1375

FENG, GUANGGANG: 7020

Feng, jingchao: 7355

Feng, Yuan: 7435

Feng, Zhaohong: 8000

Fennell, Charles: 7475

Fennell, Gillian: 1070, 7120

Fenstermacher, Ryan: 7435

Ferdows, Nasim: 1020, 2260, 7210, 7395, 7535, 9045, 9150

Fergen, Joshua T: 4045

Ferguson, Dee: 1405

Ferguson, Erin: 4280

Ferguson, Giselle: 1175

Fermin-Martinez, Carlos A: 1155

Fernandes, Cecilia: 2500

Fernandes, Karl JL: 7415

Fernandes, Marta: 7015

Fernandes Junior, Ezemir Dantas: 4425

Fernandez Cajavilca, Moroni D: 2135, 4260, 7185

Fernandez Castello, Ana Isabel: 8015

Fernandez-Romero, Roberto: 8000

Ferrante, Lauren: 7000

Ferraro, Kenneth F: 1080

Ferrarotti, Tina: 9090

Ferreira Leitao Azevedo, Renato: 1505, 4320

Ferrie, Joseph P: 3330

Ferris, Annie: 7410

Ferrone, Christine F: 7410

Ferrucci, Luigi: 4295, 4495, 7315, 8000

Feryn, Alicia: 1460

Fessele, Kristen L: 8005

Fete, Zoe: 2285, 7010, 7185, 7915, 8015

Fick, Donna M: 3205, 4015, 7060

Fiehn, Oliver: 4495, 7315

Fielding, Roger: 3160

Fields, Beth: 1035, 2445, 4435

Fields, Bronwyn: 2450

Fields, Erica Barnes: 4115

Fields, Maizonne: 8015

Fields, Noelle L: 8010, 8015

Fierro, Cecilia: 8010

Figueiro, Vittoria: 2265

Figueroa, Luis A.: 8010

Fillmore, Nathanael: 8000

Finamore, Victoria: 3355

Finch, Brian: 4030

Findley, Kimberly: 2290, 2445

Finegan, Kerry: 8010

Fingerman, Karen: 3185, 7350, 7390, 8005

Finik, Jackie: 2475, 4505

Fink, Howard A: 3065

Finkel, Deborah G: 4030, 7325, 7375, 8005, 8010

Finkelstein, Ruth: 3385

Finlay, Jessica: 2005, 2400, 4110, 4195, 4505, 7465

Finn, Laura: 8010

Finn, Myrna: 1130, 3155

Fiori, Katherine L: 2085, 4360, 7355

Fique, Morgan T: 7455, 9115

Firman, James Paul: 1515, 3215

Fischer, Mya: 2235

Fischer, Rosa-Linde: 8010

Fischer, Stacy M: 2320

Fish, Anne: 8010

Fish, James: 8010

Fishbein, Kenneth W.: 8000

Fisher, Carla: 7350

Fisher, Dr. Anna: 4405

Fisher, Koren L: 7310

Fisher, Lauren: 1015

Fisher, Louis: 9025

Fishman, Paul: 8010

Fisk, Calley: 4440

Fiske, Amy: 1440, 2240, 3100, 7255, 8010

Fitzgerald Wolff, Sharon: 7180

Fitzgibbons, Joanna: 7170, 7290

Fitzpatrick, Neasa: 7490

Fix, Rachel M: 8000, 9095

Flaherty, Grace K: 7545

Flanagan, Jane M: 8000

Flatt, Jace D: 1000, 1420, 2010, 2145, 2550, 7110, 7145, 8010

Fleck, Jessica: 7015, 7150, 7385

Fleet, Alexa: 3155, 7020

Fleming, Jessica: 7060

Fleming, Nicole: 4240, 8010

Fleser, Jessica: 9015

Fletcher, Aisha D: 4250

Flood, Johnna: 9105

Flood, Sarah: 8015

Flores, Belinda: 3355

Flores, Elena: 7190

Flores, Emily V: 7310

Flores, Micayla: 8000

Flores, Vanessa: 8005

Flores Romero, Karla Renata: 4070

Floyd, Alex: 1145, 7500

Floyd, Alex C: 9005

Fogelberg, Donald: 8000

Fogle, Stephen J: 2500, 3140

Foley, Jessica: 1520, 9120

Folker, Ann: 7350

Folorunsho, Sunkanmi: 7200

Fonarow, Gregg: 9020

Fong, Daniel Yee Tak: 4585, 7085

Fong, Florence: 9085

Fong, Steve Fu-Fai: 9085

Fong, Tamara G: 2505, 8010

Fons, Brian R: 4415, 7155, 7255, 7465

Fonseca, Luciana: 8000

Fonseca Valencia, Carolina: 7310

For, Wing-Yin: 1135

Foran, Shannon: 7410

Ford, Cassandra: 4045

Ford, James H: 2135, 7420, 7905

Ford, James C: 8015

Forden, Jessica: 3310

Foreman, Eric R: 8015

Formson, Maud: 8010

Forrester, Sarah N: 1415

Fortier, Catherine: 8005

Fortinsky, Richard: 1110, 1315, 1385, 2185, 4495, 7305, 7345, 8005, 8010, 9020, 9090

Fosnight, Susan: 4600, 7225

Foster, Charlie E M: 2120

Foster, Marcel: 9110

Foster, Norman: 8000

Fowler, Mackenzie E: 3095

Fowler, Nicole: 1435

Fox, Aimee: 1390

Fox, Julia: 7535

Fox Ludden, Emily: 8010

Fracassi, Anna: 8010

Fraiman, Paule-Sarah: 1485, 7410

Fram, Maryah S: 1090

France, Michael T: 9150

Francis, Dania V: 3310

Francis, Hannah: 1365

Francis, Howard: 7530, 8005

Francis-Levin, Jess: 3230, 8010

Frankel, Emily L: 7130

Franklin, Heather: 1165, 7415

Franzosa, Emily: 1305, 3315, 4205, 7270

Frase, Robert T: 2405, 4040, 4185, 7085

Fraser, Sarah: 7205, 8015

Freburger, Janet: 8005

Freddolino, Paul P: 3215, 4320

Fredman, Steffany J: 7380

Fredrickson, Barbara L: 1275

Fredriksen-Goldsen, Karen: 2155, 2525, 7355, 7535

Free, Peyton: 7305

Freeborn, Todd: 4475

Freedman, Vicki A: 2375

Frees, Traci: 8010

Freitag, Simone: 7125

French, Chloe: 9085

Frerichs, Leah: 8005

Freund, Alexandra: 7460

Freund, Katherine: 3170, 7155

Frey, Scott: 2215

Freytes, I Magaly: 7350

Friberg-Felsted, Katarina: 2455, 4355, 4555, 8005

Fried, Jack J: 4305

Fried, Terri R: 3290

Fried, Terri: 7030

Friedberg, Daniel: 4007

Friedland, Hannah: 7005, 7110

Friedman, Batsheva: 8010

Friedman, Ben: 7140, 7905

Friedman, M. Isabel: 7215

Friedman, Mackey R: 2140

Frimpong, Eric: 1075, 7065, 7240, 7375

Frisoli, Alberto: 7320

Fritz, Julie: 8005

Froberg, Ruthann: 9135

Frost, Alanna F: 8015

Frost, Bess: 8005, 8010

Fruhauf, Christine A: 4075, 9090

Frydenlund, Markus: 7205

Frye, Larry: 7545

Fu, Hannah: 9075

Fu, Jason: 7440

Fu, Lillian X: 2095, 4515

Fu, Peipei: 1180

Fu, Rong: 3125, 7145

Fu, Xiaoyu: 1225, 7085, 7375

Fuccione, James: 1335

Fuentes, Carlos G: 3305

Fuentes, Patricio: 9035

Fugate-Whitlock, Elizabeth: 1390, 2330

Fuglestad, Paul: 4095

Fujiwara, Takeo: 7345

Fujiwara, Yoshinori: 7265

Fukushima, Kiyoko: 7160, 7405

Full, Kelsie M: 9030

Fullen, Matthew: 7255, 7375

Fuller, Heather: 2290, 4045, 4165, 8000

Fuller, Patrice M: 1280, 7475

Fuller, Shannon: 4020

Fulop, Tamas: 7315

Fulp-Cooke, Rachael S: 2450

Fulzele, Sadanand: 7245, 8010

Fung, Chi Tat: 8000

Fung, Helene: 1330, 1350, 2250, 2455, 3360, 4300, 7005, 7375, 8010, 9070

Fung, Katherine K.: 8010

Fung, Long Ki: 2455

Fung, Nicole Long-ki: 1330, 8005

Fung, Teresa: 7440

Fung, Victor: 2165

Funk, Cory: 7415

Funkhouser, Dillon: 4435

Furey, Samantha: 7460

Furht, Borko: 2045

Furihata, Ryuji: 2230

Furman, David: 4107

Fussell, Elizabeth: 8015

Futterman, Andrew: 7365, 7400

G

Gabriel, Kelley Pettee: 2535

Gad, Dustin: 2095, 8005, 8010, 9000

Gadbois, Emily A: 1465, 2055, 7050, 7220, 7485, 7930, 8000, 8015

Gadkari, Gauri: 3280, 7185, 7420, 7930, 8005

Gage, Courtney A: 7355

Gai, Junjie: 7515

Gale, Seth: 7185

Galea, Sandro: 3315, 7450

Galecki, Andrzej: 4450

Galik, Elizabeth: 2090, 2350, 3220, 4460, 8005

Galinsky, Arielle: 7350

Gallagher, Virginia: 4485

Gallagher Thompson, Dolores: 2240, 7230, 7305, 8000

Gallo, Joe: 4150

Gallo, Linda: 7535

Galperin, Irina: 7200

Galucia, Natalie: 8000, 8005, 8010

Galvez, Maria: 3025, 8000, 8005, 8015

Galvin, James E: 4045

Gamaldo, Alyssa A: 2280, 3060, 7325

Gamble, Laura D: 2335

Gampetro, Pamela J: 1505

Gan, Mantang: 7470

Gan, Siqi: 7010, 7140

Gan, Wenqi: 4135

Gandhari, Sanjeela: 8015

Ganguli, Sohini: 8010

Gans, Hannah: 2370

Gantumur, Zoljargalan: 4325, 8015

Ganz, David A: 8000

Gao, Alvin: 2395

Gao, Bruce: 8015

Gao, Lan: 4335

Gao, Louise (Siyu): 1010, 7405

Gao, Mingui: 7255

Gao, Qi: 4310

Gao, Qinglin: 7130, 7225, 7255, 7300, 7905

Gao, Shangbang: 7190

Gao, Sibo: 7350, 8005

Gao, Sujuan: 7010, 7910

Gao, Sunan: 2100, 3105, 4375

Gao, Weijing: 9150

Gao, Xiang: 2150

Gao, Ying: 4585

Gao, Yuhan: 8000

Gao, Zan: 9025

Gao, Zhengrong: 2245

Gara, Nabel: 8005

Garabedian, Pamela: 8015

Garbe, Renee: 3300

Garbin, Alexander J: 7410, 7910

García-Lara, Juan M A: 7145

Garcia, Adolfo J: 8015

Garcia, Catherine: 1225, 4290, 4315

Garcia, Cheyenne M: 4285

Garcia, Emily: 7370

Garcia, Gabriela: 7285, 7925

Garcia, Gilberto: 4275

Garcia, Kandyce: 2170

Garcia, Lorena: 2450

Garcia, Marc A: 4290

Garcia, Patricia: 3085

Garcia, Reagan E: 8005

Garcia, Zoraida: 7145, 8000

Garcia De La Garza, Angel: 4310

Garcia Morales, Emmanuel E: 4065

Garcia Walker, Veronica: 8010

Garcia-Arias, Sabrina: 8005

Garcia-Chanes, Rosa: 2060

Garcia-Davis, Sandra: 8010

Garcia-Jaramillo, Manuel: 4347

Garcia-Morales, Emmanuel: 1075

Garcia-Retortillo, Sergi: 8005

Gardiner, Lea: 7000

Gardner, Bailey: 2555, 3010, 7010

Gardner, Raquel C.: 7000

Gardner, Sue E: 7265

Gareri, Michele: 7225, 7340, 7920

Garner, Charity: 8005

Garnett, Anna: 1430

Garrido, Melissa: 1190

Gary-Webb, Tiffany L: 2200

Garza Naveda, Ana Paola: 9120

Gaspar, Lauren: 1520

Gasser, Brent: 7160

Gassoumis, Zachary: 1215, 3300

Gatchel, Jennifer: 7415

Gately, Megan: 2365, 7295, 7310

Gatt, Justine M.: 4470

Gattine, Elizabeth: 9115

Gatz, Margaret: 4030

Gau, Wen-Bing: 7080

Gaugler, Joseph E: 1280, 2010, 2305, 3117, 3155, 3375, 4100, 7300, 9030

Gavett, Brandon: 4540

Gay, Emma L: 8000

Gay, Whitney: 1060

Gaydosh, Lauren: 8015

Gaylord, Susan: 4535

Gazaway, Shena: 3165

Gaziano, J.Michael: 8010

Gazit, Eran: 7200

Ge, Jing: 4370, 7310

Ge, Linghan: 4315

Ge, Meiling: 9150

Ge, Shaoqing: 4065, 4225, 7475, 9010

Geary, Elizabeth K.: 7330, 7390

Geisser, Sophia R: 2240, 4475

Geisser, Zoe R: 2240

Gell, Nancy M: 7265

Geller, Aaron M: 4515

Gelmon, Sherril B: 7010

Gems, David: 4473

Généreux, Mélissa: 4150

Geng, Fangli: 7125, 7185, 8000

Genova, Helen: 8015

Gensoli, Lindsey: 2275

George, Claudene: 4200

George, Kristen: 7435

George, Megan: 4525

George, Miriam: 2465

Geraets, Anouk: 1105

Gerasta, Denae: 2320, 8015

Gerhard, Glenn: 9070

Gerlick, Joshua A: 7410

Gerosa, Laura: 4473

Gershon, Richard C: 7435

Gerstorf, Denis: 1350, 2120, 4050, 4490, 4515, 7380, 7390, 8000

Gesel, Paul: 4005

Getahun, Darios: 7110

Gettel, Cameron: 7485, 8015, 9140

Getz, Marjorie A: 7175

Gevaert, Jessie: 1425

Geyskens, Lisa: 1060, 8010

Ghaemmaghamfarahani, Ida: 8010

Ghaiye, Suruchi: 8015

Ghanem, Karyman: 7230

Ghanta, Manohar: 7015

Ghattas, Ola: 4005

Ghazi Saidi, Ladan: 4045, 7325

Gheller, Mary: 7120

Ghemrawi, Mirna: 9070

Ghiasi, Akbar: 3365

Ghimire, Dirgha J: 1500

Ghimire, Saruna: 4465

Ghodsi Jafari, Aveen: 7305

Gholizadeh, Shadi: 8005, 8015

Ghose, Urmimala: 1350, 7265, 7380, 8000

Ghosh, Indrajit: 4465

Giammanco, Peter Aldo: 7485

Gianaros, Peter J: 2200

Giandrea, Michael D: 3310

Giannakopoulou, Parthenia: 3030

Giannetti, Matthew: 8000

Giannotto, Emily L: 8005

Giard, David: 1305

Giatras, Ava: 2445

Gibbons, Anna: 8015

Gibson, Allison: 2110, 2335, 8000

Gibson, Grant: 9100

Gibson, Regina: 8015

Gider, Nathalie: 3370, 4030

Gieniusz, Marzena: 2175, 8000

Giffuni, Jamie: 7140, 7905

Gil, Efrat: 7210

Gil, Heidi: 7440, 7915, 8000

Gil Hayes, Lauren: 7260, 7290, 7925

Gilbert, Lauren R: 3305

Gilbertson-White, Stephanie: 8000, 8015

Gilfix, Talia: 7435

Gilhuly, Devin: 7290

Gill, Amarjot S: 7370

Gill, Christine: 7515, 7930

Gill, Thomas M: 1375, 8010

Gillespie, Kate: 7185

Gillett, Alexis: 7265

Gilligan, Megan: 2405, 3185, 4040, 4185, 4315, 4485, 7085, 7470, 8000

Gilliland, Jaime: 2220, 8005

Gillis, Elizabeth: 4500

Gills, Joshua L: 7120

Gilmartin, Mattia J: 1485

Gilmore-Bykovskyi, Andrea: 7000, 8005

Gilsanz, Paola: 8010

Gimm, Gilbert: 2190, 2375, 7065, 7900

Ginggeaw, Sangduan: 4135

Ginsberg, Charles: 7345

Ginsberg, Terri: 4570

Giordani, Bruno: 1510

Giordano, Stephanie J: 1010, 2260, 3020, 3190

Giorgio, Luciana: 3245

Giraldo-Santiago, Natalia: 2310

Girling, Laura: 8005

Giroski, Lexi B: 7200

Gitelman, Darren R: 7010

Gitlin, Laura N: 1320, 3085, 4210, 7300, 7415, 8005

Gittleson, Alondra: 2275

Giudicessi, Averi: 8000

Gjika, Olga: 8010

Gladfelter, Jenna: 7540

Gladyshev, Vadim: 8005

Glaser, Karen F: 3225

Glavin, Ye-Fan Wang: 2305

Gleason, Carey: 1160, 2170, 7270

Gleason, Kathy S: 4090

Gleason, Kelly T: 1360

Gleason, Lauren J: 2205

Gleason, Shayna R: 3170

Gleason, Shayna R: 4130

Gleason, Shayna R: 7155

Glei, Dana A: 3285

Glenn, Jordan: 7120

Glennie, Kristen S: 7300

Glesby, Marshall J.: 1490

Gliebus, Peter: 8005

Glinka, Allison: 7305

Glorioso, Danielle: 7050, 7375

Glose, Susan: 7005

Glover, Crystal M: 2550

Glymour, M. Maria: 4280, 7260, 8000, 8005, 9040

Glynn, Nancy W: 8000, 8005

Goble, Daniel J: 8010

Goetz, Matthew B: 8000

Gofine, Miriam: 1505, 7355

Goh, Denise: 8005

Goh, Jing Wen: 2435

Goh, Joshua O S: 4030

Goh, Sarah: 4370

Golant, Stephen: 4340

Gold, Noah: 8000

Goldberg, Emily: 2277

Goldberg, Robert: 2500, 7485

Goldblatt, Hadass: 1240

Golden, Robyn L: 9140, 9145

Golden-Pogue, Andrea L: 8000

Goldman, Alyssa: 1105, 3050, 3285

Goldman, Annika: 2085

Goldman, Max: 8015

Golland, Yulia: 4235

Gollinelli, Daniela A: 8005

Golzarri-Arroyo, Lilian: 2555, 3010, 7010

Gomes Goncalves, Natalia: 4540

Gómez, Bárbara: 8015

Gomez, Elisa: 7470

Gomez, Rowena: 8015

Gómez-Martínez, Héctor M: 7200

Gomez-Lopez, Iris: 1370

Goncalves, Marcus D: 3105

Gondo, Yasuyuki: 7365

Gong, Caixia: 1220

Gong, Jessica: 1040

Gong, Jiaowei: 4185

Gonyea, Judith G: 4575

González-Rivera, Christian: 3385

Gonzales, Alexander: 3300

Gonzales, Ernest: 2530, 3045, 7005, 8000, 8015

Gonzales, Mitzi: 1387, 8010, 9020

Gonzalez, Alexa S: 3380, 4540

Gonzalez, Anelly: 7330

Gonzalez, Evelyn: 2005, 2400

Gonzalez, Hector M: 2040, 7535

Gonzalez, Jeffrey S: 4310

Gonzalez, Karen: 2140

Gonzalez, Richard: 2375, 7230, 7335

Gonzalez, Rodriguez: 7010

Gonzalez, Vanessa: 8010

Gonzalez Pyles, Maria S: 7305

Good, Mary K: 1305

Goodman, Michael: 7110

Goodman, Octavia K: 8015

Goodrich, Glenn: 7385

Goodson, Pauline: 9015

Goodwin, James S: 4365

Goodwin, Richard: 8015

Gooldin, Sigal: 7110

Gordon, Barbara: 1060, 2465

Gordon, Julie A: 7370

Gordon, Kate: 1395, 8005

Gordon, Kate: 8005

Gordon, Kate: 8010

Gore, Janelle E: 9025

Gore, Shweta: 8015

Gorman, Emily F: 4465

Gorman, Lily G: 8015

Gorospe, Myriam: 7480

Gorzelle, Gregg: 8005, 9010

Gosselin, Catherine: 7325

Gosselin, Patrick: 8000

Gotanda, Hiroshi: 8000

Gothe, Neha P: 1255, 7265, 8000

Goto, Ryohei: 8010

Gottesman, Elaine: 3300

Gottesman, Rebecca: 1200, 2535, 7325

Gotwals, Amy E: 2355

Gourley, Amy N: 1210

Gouveia, Gillian: 8005

Goyal, Riya: 7015, 7385

Gozalo, Pedro L: 2160, 7125

Gozalo, Pedro: 8005

Grabowski, David: 2465

Gracia, Deborah: 8000, 8015

Grady, James: 1385

Graf, Allyson S: 3150

Graham, Eileen K: 1265, 4120

Graham, Helen L: 8010

Graham, Kirsten L: 7455, 7935

Grahama-Engeland, Jennifer E: 1445, 7535, 8015

Gramling, Robert: 7395

Grams, Morgan E: 1155, 3105

Granada, Tara: 7340

Granbom, Marianne: 4215

Grande, Giulia: 4370

Grant, Claire: 2030, 7230, 7915

Grant, Haley: 4295

Gras, Laura: 7140

Grasso, Anna: 2380

Gravely, Amy A: 3065

Gravenstein, Stefan: 1435, 7305

Gravenstein, Stefan: 8005

Gravenstein, Stefan: 8010

Graves, Letitia: 8010

Gravett, Christopher J: 7405

Gray, Jullie M: 7170

Gray, Margeaux: 7535

Gray, Michelle: 7120, 7325

Gray, Serena: 7400

Gray, Tamryn: 3355

Greaney, Mary A: 2255

Greco, Carol: 4535

Green, Ariel R: 4090, 7385

Green, Brian: 1050

Green, Charles: 8000

Green, Gavin: 4085, 7215, 7330, 8005

Green, Gizelle: 3040

Green, Michael D: 4095, 8000

Green, Nathan: 9065

Green, Noah: 7435, 8010

Greenberg, Jonathan: 2310

Greenberg, Phyllis A: 7395, 7455, 7935

Greenberg, Sherry A: 7410

Greenberg, Steven M: 2460

Greenfield, Emily: 2170, 2185, 3000, 4130

Greenfields, Margaret: 1325

Greenwood-Hickman, Mikael Anne: 9090

Greger, Jeffrey S: 8010

Gregg, Jay: 2085

Grenier, Sébastien: 8000

Grenko, Lydia K: 7395

Grenz, Adele: 3080

Grey-Coker, Sierra: 3345

Grier, Kimberlee: 7305

Griffin, Hannah: 7455, 7935

Griffith, Christopher X: 7195, 7900, 8010

Griffith, Lauren E: 2545, 7225, 7905

Grill, Joshua D: 3375, 7135, 7335

Grillo, Anthony: 8000

Grindrod, Kelly: 7230

Grippo, Angela: 7040

Griswold, Michael: 2535, 7025, 7325, 7495

Groden, Sheryl R: 7295

Grodsky, Eric: 2120, 7010, 7040

Grodstein, Francine: 8000

Grogan, Elyssa FL: 8015, 9140

Grol-Prokopczyk, Hanna: 1070, 4590

Gronlund, Carina: 7465

Gross, Alden L: 1495, 2000, 2035, 3380, 4060, 4280, 4540, 7040, 8010, 8015, 9045

Gross, Alina T: 9115

Gross, Danae: 1220

Gross, Hana: 7435

Groves, Angela: 7320

Groves, Patricia S: 7225

Growdon, Matthew: 7255, 8005, 8010, 9000

Growney, Claire: 1045, 1195, 1330, 3270, 4040

Gruber-Baldini, Ann: 2395, 7545

Gu, Danan: 9030

Gu, Tongyu: 8015

Gu, Yuchen: 7355, 7470

Gu, Yunke: 8000

Guan, Jean: 1345

Guan, Weihua: 7345, 7425

Guan, Xiangnan: 8010

Guan, Xiwen: 8015

Guay, Sandryne: 7200

Guengerich, Terri: 3070

Guenther, Ariana G: 8015

Guerrero, Omar: 7210

Guest, M. Aaron: 2030, 2455, 4355

Gugliucci, Marilyn R: 3130, 7455, 7935, 8015

Gugliuzza, Lydia: 8015

Guida, Cassidy A: 8005

Guidotti Breting, Leslie: 7330, 7390

Guion, Kent: 7005

Gunning, Faith: 7460

Gunter, Kabreah: 8005

Guo, guifang: 7430

Guo, Jia-Wen: 2030, 8010

Guo, Jin: 7470, 7900

Guo, Man: 1250, 7260, 7290, 7375, 7925

Guo, Muqi: 1495

Guo, Sunan: 2100

Guo, Tangmeng: 4370, 7310

Guo, Tianming: 4505

Guo, Xueying: 7070

Guo, Yinning: 2545, 7090

Guo, Zheng: 3275

Gupta, Aditi: 7090

Gupta, Aditya: 7015

Gupta, Avinash: 8005, 9065

Gupta, Ritom: 7225

Gupta, Santosh S: 2500

Gupta, Tanisha: 9070

Guptarak, Jutatip: 8010

Gurera, Jacob W: 7460

Gurevitch, Jacqueline: 2240

Gurinovich, Anastasia: 8005

Gurlu, Merve: 2350

Gurung, Adina: 1500

Gurwitz, Jerry H: 1435, 2500, 7410, 7485

Gur-Yaish, Nurit: 7000

Gusen, Nanle Joseph: 7305

Gusmano, Michael K: 3335

Gustafson, Deborah: 2395

Gustafson, Deborah R: 8015

Gut, Philipp: 8010

Gutchess, Angela: 4030, 7200

Gutiérrez, Myriam: 8015

Gutierrez, Angela: 3265, 4110, 4125, 4250

Gutierrez, Angelina: 4415, 7155, 7255, 7465

Gutierrez, Carmen: 3235

Gutierrez, Sirena: 3380, 7465

Gutierrez-Martinez, Leidys: 9040

Gutierrez-Ramirez, Paulina: 3025, 8000, 8005, 8015

Gutman, Itai: 2245

Guttoo, Parishma: 8010

Guzman, Benedict: 7185

Gwalani, Pranav: 8000

Gwizdala, Crystal: 8005

Gwon, Nayeong: 7035

H

Ha, Jung-Hwa: 3045

Haack, Linda: 1035

Haardoerfer, Regine: 2160

Haas, Ashley: 1395

Haas, Steven: 4470

Haase, Claudia M: 2095, 4515, 7265

Habadi, Assma: 7015

Hacker, Eileen: 7265

Hackett, Katherine: 9080

Hackett, Sara: 7340, 7935

Hackney, Madeleine E: 7225, 7440, 9110

Haddad, Zena: 8000

Hadson, Kimberly: 4155

Hagan, Anne: 7290, 8005

Hagan, Brenna: 8000

Hagan, Kelsey E: 7375

Hage, David: 7485

Hagedorn, Aaron: 7300

Hagemann, Lauren: 7230

Haggar, Katherine: 3050

Haggerty, Kristin Lees: 2285

Hagiwara, Shotaro: 8015

Hahn, Samantha L: 7145

Hahne, Jessica: 8000

Haig, Sarah: 4305

Haigh, Sylvia V: 2225, 3040, 4240, 4550

Hajjar, Ihab: 2360

Hakanson, Megan: 7370

Halasz, Ildiko: 7040

Halevi Hochwald, Inbal: 8005

Haley, William E: 2490, 7350, 7420, 7425, 8010

Halkjær, Jytte: 7345

Hall, Charles B: 2400, 4310

Hall, Katherine S: 1470

Hall, LaToya: 2115, 7310

Hall, Lauren M: 1305, 3315, 4205, 7270

Halladay, Christopher: 9090

Haller, Nico: 7125

Halliday, Caroline: 7545

Halloway, Shannon: 4570

Halpin, Sean N: 2220, 8010

Halvorsen, Cal: 1195, 1425, 2170

Halwani, Zena: 7350

Ham, Hyunsun: 8015

Hamada, Fumiko: 7290, 7390

Hamada, Shota: 7250, 7465

Hamada, Shota: 8015

Hamasaki, Yoko: 8010

Hamatsu, Carolina E: 8000

Hamers, Jan: 1310

Hamers, Maud: 3035, 7370

Hamill, Randi: 2260

Hamilton, Lucas J: 1095, 2360, 9030, 9055

Hamler, Tyrone C: 3245, 3395, 8000

Hamlin, Abbey M: 4570, 8005

Hamm, Jeremy: 1175, 7325, 7380

Hamm, Sara: 2275

Hammel, Edward: 7320

Hammel, Iriana S: 2195, 3065

Hammill, Bradley G: 8000

Hampton, Marsha: 3060

Hamrick, Mark: 7245

Han, Bing: 4470, 7210

Han, Chengming: 1180, 3045, 7240, 7445, 7535

Han, Donghee: 7445

Han, Duke: 1100, 3117, 3185

Han, Gaeun: 7080

Han, Gyounghae: 7230

Han, Hae-Ra: 1345

Han, Jian: 8005

Han, Ling: 8015

Han, Lisa F: 7305

Han, Mo: 4590

Han, Sae Hwang: 1195, 2025, 8015

Han, Seok Gyu: 8000

Han, Song: 1110

Han, Soojeong: 7125

Han, Xiaowen: 1180

Han, Xijing: 9030

Han, Y. Catherine: 7435

Han, Yeomin: 9065

Han, Ying: 1235

Han, Yunji: 7155

Hanchate, Amresh D: 1255, 4065

Hancock, Cynthia: 2330

Hancock, David: 8005

Hand, Kathryn: 8000, 8005

Hand, Michelle D: 2165, 9100

Handa, Satoru: 7225

Handique, Swasati: 8010

Handler, Steven M: 4305

Handler, Steven: 4305

Handler, Steven M: 7225, 7515

Haneuse, Sebastien: 9155

Haney, TaFarra: 7220

Hang, Ling: 7090

Hang, Qiqi: 4505

Hankes, Michael: 2245

Hankinson, Susan: 8005, 9155

Hanley, Meredith: 2495

Hannan, Marian: 9155

Hanratty, Barbara: 7400

Hansmann, KJ: 4435

Hanson, Laura: 3015, 3290

Hao, Shira: 8005

Haozous, Emily A: 3345

Happ, Mary Beth: 8005, 8010

Happel, Christine M: 1000

Haque, Nashua: 8000

Harada, Ann SM: 1455

Harada, Ko: 4425

Harden, J Taylor T: 4190

Hardt, Madeleine M: 2220

Harel, Dovrat: 4235

Harel, Ofer: 7345

Harezlak, Jaroslaw: 3085, 7415

Hargand, Sari: 7280

Hariri, Ahmad: 7025

Harley, Dumichel: 9110

Harmon, Misty: 8010

Haro-Ramos, Alein Y: 4095

Harrington, Karra: 2360

Harrington, Samantha: 2365

Harris, Daniel A: 7305, 9090

Harris, Emily: 8000

Harris, Lauren: 8005

Harris, Maurita T: 9110

Harris, Megan: 8005, 8015

Harris, Rebekah: 1470, 7040, 8000, 8010, 9095

Harris-Gersten, Melissa: 7350, 7930

Harrison, Ben: 7045, 7315

Harrison, Krista L: 2185, 9000

Harrison, Tracie C: 7165, 8010

Hart, Alexis: 7340, 7920

Hart, Autumn: 8015

Hart, Evelyn Grace: 8000

Hart, Peter D: 8010, 8015

Hart, Sara E: 4555

Hartley, Gregory W: 7075, 7140

Hartley, Sigan: 8000

Hartlieb, Kristen: 7020

Hartmann, Christine W: 1300, 4475

Hartmann, Julie A: 7340

Hartung, Heike: 2140

Hartz, Stephanie M: 7410, 7910

Harvey, Idethia Shevon: 7430, 8005

Harvey, Jessica: 2120

Harwell, Carla: 8010

Hasan, Sayed: 7190

Hasan, Wordh Ul: 3340, 7015

Hasan, Wordh Ul: 8000

Hasche, Leslie: 1515, 8005

Hasdell, Peter: 7465

Hasegawa, Nanako: 7250

Hashemzehi, Amin: 1065, 7395

Hasin, Deborah: 4390

Hasnani, Madison E: 7500

Hassan, Malaz: 4190

Hassel, Bret: 7455

Hasselgren, Caroline: 7375

Hassmiller Lich, Kristen: 8005

Hastings, Susan: 1470, 4430, 4565, 7350, 7930

Hastings, Waylon J: 8005, 8010

Hasworth, Serena B: 8015

Hatch, Melissa A: 7465

Hathaway, Wendy: 8015

Hattakitjamroen, Varitnan: 4080, 8005

Haub, Mark: 7130

Haurin, Donald: 3285

Hausdorff, Jeffrey M: 7200

Haverhals, Leah M: 1300, 8000, 8015, 9095

Hawes, Courtney: 7050

Hawes, Frances: 4007, 8010

Hawkley, Louise: 1445

Hawks, Zoë: 1265, 1265, 4120

Hawley, Chelsea: 8015

Hayashi, Atsuko: 7140

Hayden, Mary: 3235

Hayek, Roee: 1220, 2245

Hayes, Cellas: 1340

Hayes, Courtney: 4205

Hayes, Jessica M: 1255

Hayes, Kaleen N: 7305

Hayes-Larson, Eleanor: 2390, 4280, 8000, 8010

Hayman, Laura L: 7200

Haynes, Laura: 8000

Haynos, Ann F: 7375

Hays, Ron D: 3290

Hayslip Jr., Bert: 4085, 7470

Hazelett, Sue: 4600, 7150, 7340, 7920

Hazlett, Susan: 2485

Hazuda, Helen P: 1455

He, Caihong: 7130

He, Diana: 8000

He, Jasmin: 1215

He, Jianghua: 7225

He, Jie: 8000

He, Juanjuan "June": 9115

He, Junjie: 1160, 2105

He, Ping: 4370, 7310

He, Ranran: 2405, 4040, 4185, 7085

He, Rendong: 2480, 4070

He, Wenqian: 2275

He, Yilin: 7015

He, Zhe: 1285

He, Zhe: 8000

He, Zhe: 8010

Heath, Diana M: 1275

Heaton, Karen: 4140

Hebdon, Megan: 2190

Heber-Brown, Jennifer: 2330

Heck, Andrew: 2240

Heck, Jennifer: 7005

Heckbert, Susan R: 2400

Heckmann, Kimberly: 7455

Heckman-Stoddard, Brandy: 1455

Hed, Sara: 4010, 7255

Hedges, Hanna: 8010

Heeren, Amanda: 1305

Heffernan, Kevin S: 2085

Heffner, Kathi L: 2310

Heflin, Colleen M: 3285

Heid, Allison R.: 1300, 7305

Heiland, Frank: 3385

Heilman, C. Cassie: 7475

Hein, Maria L: 1150, 1515, 2225, 3220, 4015, 7250

Heinemann, Laura L: 3140

Heintz-Monette, Hannah L: 7375

Heinz, Melinda: 4035, 7015, 7150, 7220, 7275, 7285, 7350, 7380, 7410

Heinze, Rebecca G: 8005

Heist, E. Kevin: 7510

Heitner, Rachael: 9145

Heller, Sloane: 7010

Heller, Tamar: 2430

Helm, Faith: 2205

Helmer, Dashiell: 8005

Helmly, Victoria: 2485

Helsabeck, Nathan P: 2135

Hemmy, Laura: 8010

Hemrajani, Girish: 7515

Hendricks, Justin: 2405

Hendricks-Ferguson, Verna L: 4430, 7220

Hendrix, Cristina: 3355

Henke, Maria: 7300

Henkens, Kene: 2350

Henninger, William: 7285, 7350

Henning-Smith, Carrie: 1010

Henricks, Evan: 3240

Henry, Kevin: 2400

Henry, Maureen: 2075

Henry, Samantha: 8000

Henry, Yater: 7385

Heo, Jinmoo: 7065, 7365

Heo, Jun: 8015

Heo, Seok-Jae: 7325

Hepburn, Kenneth: 1275, 2160, 3375, 4485, 7015, 7475, 8010

Hepple, Russell: 4275

Herbeck, Brandon J: 2485

Herd, Pamela: 4585

Hermens, Daniel: 4580

Hernández, Diana: 7040

Hernández-Ruiz, Virgilio A: 7145

Hernandez, Adriana: 7030, 7050

Hernandez, Mario A: 4080

Hernandez, Rolando J.: 8000

Hernandez-Chilatra, Jessica A: 7015

Hernandez-Tejada, Melba: 4415

Herrera, Carolina: 9125

Herrick, Ashton: 7160

Herrick, Tamara H: 7225

Herring, Laura: 1220

Herzberg, Suzanne: 7410

Herzig, Sébastien: 8010

Herzog, Naemi: 8015

Heselton, Hyeon Jung: 7150

Hess, Moritz: 3080

Heston-Mullins, Jennifer L: 4365, 7540, 8015

Hetherington, Allisyn: 8005

Hetherington-Rauth, Megan: 4473

Hewner, Sharon: 7370

Heyman, Janna C: 7355

Heyn, Patricia C: 2070, 2175, 4190, 9065

Hicken, Bret L: 1305, 2365

Hickey, Johanna: 1435

Hickman, Louis: 3270

Hickman, Susan: 1145, 1435, 8010

Hickner, Andy: 3025

Hicks, Daniel: 4275

Hicks, Stephanie: 8005

Hicks-Patrick, Julie: 3335, 7005, 7350, 7475

Hiersteiner, Dorothy: 3190

Higareda-Garza-Ramos, Andrea: 7145

Higgins, Connor: 8000

Higgins, Maureen: 7225

Higgins, Melinda: 7475

Highberg, Nels: 4220

Higuchi-Sanabria, Ryo: 4275

Hilderbrand, Zoey: 4350

Hilgeman, Michelle M: 1060, 2085, 4240, 7105, 8005

Hill, Carl V: 3180, 4125, 4160

Hill, Cristal: 2417, 4227

Hill, Jordan R: 2555, 3010, 7010

Hill, Nikki L: 8000, 8005

Hill, Patrick L: 4040, 8005, 8015

Hill, William D: 8010

Hill-Jarrett, Tanisha G: 2200, 4280, 8015, 9110

Hillman, Charles: 7265

Himmelfarb, Cheryl R: 7380, 7430, 7495

Himmelmann, Laura: 4450, 7200

Hinkle, Janice: 8000

Hinneh, Thomas: 8015

Hinrichs-Kinney, Lauren A: 7140

Hinyard, Leslie: 4430, 7220

Hirata, Takumi: 7365, 8010

Hiratsuka, Vanessa: 1120

Hirsch, Drew: 9020

Hirsch, Jana A: 2400, 7040, 8005

Hirsch, Jenavry R: 8010

Hirschberg, Julia: 7545

Hirschman, Karen B: 1015, 8000, 9135

Hirsch-Matsioulas, Orit: 1130

Hirsch-Matsioulas1, Orit: 4355

Hirst, Jeremy: 8015

Hiss, Jacob: 7090

Hittner, Emily F: 7265

Hladek, Melissa D: 1220, 3345, 4010, 4445

Hlaing, WayWay M: 8010

Ho, Jessica Y: 1375

Ho, Ken Tsz-Kin: 1135

Ho, Rainbow Tin Hung: 4300, 7395

Ho, Roger: 8005

Ho, Tuong-Vi: 7440

Ho, Yuen Wan: 1350, 7375

Hoang, Bao: 3090

Hoang, Jason N: 7010

Hoang, Minh-Nguyet: 9025

Hobday, Amy: 7300

Hobday, John V: 2010

Hoben, Matthias: 4095

Hock, Robert: 2540

Hodes, Richard: 3033, 4008

Hodges, Sierra: 7435

Hodgson, Nancy A: 7415, 8010, 9135

Hoedjes, Meeke: 7035

Hoedl, Manuela: 1310

Hoeffgen, Gwendolyn: 8015

Hoel, Sydney: 1035, 7305

Hoenig, Helen: 7310

Hoeppner, Bettina: 1160

Hoffman, Abigail: 8000

Hoffman, Charity M: 8010

Hoffman, Elise: 4535

Hoffman, Geoffrey: 7485

Hoffman, Jessica M: 2417, 7045, 8010, 8015

Hoffman, Rashelle: 1020

Hoffman, Samantha: 7320

Hoffman, Yaakov SG: 1100, 8005

Hoffmann, Thomas: 9060

Hofman, Albert: 9040

Hogan, David B: 2545

Hogans, Beth B: 2195, 4600

Hohman, Timothy J: 7145, 9040

Holaday, Louisa W: 8000

Holden, Richard J: 2555, 3010, 3085, 7010, 7415

Holena, Sophia: 7500

Holland, Alexandra: 2005

Holley, Lyn M: 7295

Hollingshaus, Michael: 1025

Hollingsworth, David W: 4240

Hollis, Linda: 1450

Hollister, Brooke A: 7205

Hollman, Nicholas: 1510, 2245

Holm, Johanna B: 9150

Holman, Alison: 7050

Holmes, Ashleigh: 7380

Holmes, Rachel: 7415

Holmes, Sarah: 1395, 2150, 2350, 3220, 4460, 7290, 7495, 8005

Holmes, Sofia: 8005, 8010

Holt, Janet K: 1020, 2170, 4045

Homayouni, Ramin: 1370

Hon, Kristin C: 3025

Honda, Ayumi: 7070, 7235

Honda, Sumihisa: 7235

Honea, Robyn: 4585

Hong, Chenlu: 4455

Hong, Dahye: 7325, 7395, 7520

Hong, Dongfang: 7185, 7200, 7255, 7260

Hong, Eunhye: 8000

Hong, Hyeon: 8015

Hong, Ickpyo: 4525

Hong, Jiyeong: 9130

Hong, Junyuan: 8000

Hong, Kyung-Zoon: 7295

Hong, Michin: 7290

Hong, Seunghye: 7475

Hong, Song Hee: 8000

Hong, Sungjae: 4320

Hong, Sungsoo: 8015

Hong, Ting: 8000

Hong, Wan: 3340, 4420

Hong, YinHui: 2300

Hood, Michelle M: 1110, 4460, 7345

Hoogendijk, Emiel O.: 2510, 7240

Hoogendoorn, Claire J: 4310

Hooker, Julia: 7500

Hooper, Chloe: 7255

Hoppmann, Christiane: 1350, 4270, 4490, 4515, 7390, 8000, 8010

Hoque, Asef Raiyan: 7145

Hord, Norman: 4347

Horiuchi, Takashi: 7380

Horn, Amanda J: 2515

Horn, Janet: 9035

Horn, Linda B: 7455

Horn Mallers, Melanie C: 7455

Horowitz, Chloe S: 7365

Horsfall, Laura: 2250

Horstman, Molly J: 1035

Hort, Jakub: 7295

Horton, Madison: 7185

Hosgood, Dean: 2400

Hossain, MD Sarafat: 8010

Hossain, Mohammad Sajjad: 7475

Hossain, Sheikh: 8015

Hosseinian, Zahra: 7435

Hoti, Kreshnik: 7250, 7330

Hottinger, Lulu: 8000

Hotz, V. Joseph: 3050

Hou, Chien-Chou: 7165

Hou, Ryker: 8015

Hou, Su-I: 1290, 2300, 3030, 4175, 8010

Hou, Yifei: 4040

Hough, Elizabeth A: 7340

Hough, Katrina A: 4020

Houston, Denise K: 1455, 2470

Houston, James R: 7380, 8000

Houston, Leah N: 7310

Houts, Renate: 7115

Hovland, Cynthia: 4600

Howard, Alexandra: 7310

Howard, Kristen R: 8010

Howard, Marjorie: 7165

Howd, Beth: 3220

Howe, Melissa J: 4085, 8010, 9005

Howe, Rebecca: 9080

Howell, Britteny M: 1120, 3295

Howell, Cynthia: 2515

Howell, Veronica: 7375, 7395

Howland, Chelsea: 4230

Howren, Bryant: 1020

Hshieh, Tammy: 2505

Hsiao, Janet Hui-wen: 8005

Hsieh, Evelyn: 7245

Hsieh, Jessica: 7340, 7920

Hsieh, Katherine L: 1230, 9080

Hsieh, Shu-Chen: 8015

Hsu, Amy T: 7370

Hsu, Benjumin: 2125

Hsu, Clarissa W: 9090

Hsu, Erh-Chi: 7200, 8005, 8015, 9005

Hsu, Fang-Chi: 2470

Hsu, Hui-chuan: 7205

Hsu, Kelly: 7215

Hsu, Sheng-Chin: 7490

Hsu, Wan-Ling: 7205

Hsu, Yi-Hsiang: 8000

Hsu, Yu-Chi: 7090

Htoo, Zaw Wai: 7130

Hu, Elise: 1160, 3355, 7015

Hu, Haomin: 2375, 4305, 7225

Hu, Jingwen: 9075

Hu, Jinyu: 4505

Hu, Ke: 7410

Hu, Linlin: 2305, 7145

Hu, Mengyao: 1075

Hu, Rita X: 4285

Hu, Tiantian: 7015

Hu, Tiffany Y: 7410, 7920

Hu, Ting: 7365, 7405, 8015

Hu, Tingfei: 2300, 7100

Hu, Yan: 7015

HU, Yanqiu: 7305

Hu, Yaomin: 3160

Hu, Yinin: 8015

Hu, Yue: 7240

Hu, Yue: 8005, 8010

Hu, Zhiwei: 7240, 7445, 7535, 8005

Hua, Cassandra L: 3280, 8005, 8015

Hua, Nan: 4055, 7095, 7915

Hua, Shuxian: 9075

Hua, Yijing: 7030

Huang, Alison R: 1075

Huang, Alison J: 9115, 9150

Huang, Bin: 3025, 4225, 8015, 9010

Huang, Chih Yun: 7235

Huang, Chiyue: 7045

Huang, Codiez Z. D.: 1225

Huang, Jie: 7320

Huang, Jing: 3015, 4010, 4385, 7390

Huang, Jun-Rong: 7465

Huang, Liming: 8010

Huang, Qiongrong: 7190

Huang, Rui: 1070, 1370, 4590

Huang, Rui: 7025, 7195

Huang, Ruizhi: 8010

Huang, Sean: 3280

Huang, Shuo J: 4125

Huang, Siyuan: 7180

Huang, Tianyi: 8005

Huang, Wenxuan: 1180, 1505, 8005

Huang, Yan: 1125

Huang, Yen-Ming: 1365, 2135, 8005

Huang, Yi: 7365

Huang, Yuanqing: 7540

Huang, Yuncheng: 7030

Huang, Yuting: 7365

Huang, Yuxuan: 8000

Huang, Zewen: 7375

Huang, Zongan: 7015

Huanran, Liu: 7350, 7495

Hubbard, Erin: 7130, 7290

Huberty, Jennifer: 4590

Hubner, Sarah: 7095, 7280

Huckfeldt, Peter: 1455

Hudda, Urooj: 8015

Hudson, Darrell: 7260

Hue, Lisa: 8015

Hueluer, Gizem: 1350, 2120, 7350

Huemer, Marie-Theres: 1245

Huff, Hannah N: 8015

Hugenschmidt, Christina E: 8000

Hughes, Jaime M: 4205, 4525, 7390

Hughes, Jeff: 7250, 7330

Hughes, MacKenzie L: 1120

Hughes, Meredith: 4305, 7515

Hughes, Michelle: 7225

Hughes, Susan: 3045

Hughes, Timothy: 2400, 7040, 8005

Hughes, Timothy: 8005

Hughes, Timothy: 9040

Hughes, Travonia: 8005

Huisingh-Scheetz, Megan: 1220, 2100, 4200

Hulen, Elizabeth: 7135

Hulko, Wendy: 8015

Hull, Peter: 7540

Hulme, Claire T: 2335

Hultgren, Kyra: 7365, 7400

Hummel, Eleanor: 8005

Hundal, Harleen: 7440

Hung, Lillian: 2090, 2165, 3260, 7205, 7235, 7295, 7440, 7915, 8005, 8010

Hung, William: 3115, 4425

Hung, William W: 8010, 8015

Hung, Yat Tung: 8005

Hunkins, Nate: 8015

Hunt, Anne: 7340

Hunt, Lauren: 1025, 2185, 2560, 7010

Hunter, Gerald: 2200, 9110

Hunter, Heather: 7165

Hunter, Sandra K: 7120

Huo, Meng: 1275, 2025, 3185, 4485, 7185, 7335

Hurlburt, Michael: 7085

Hurley, Kelley: 2055

Hurwitz, Max: 8000

Hussain, Saad: 7185

Hussaini, Fareeha: 4230

Hussein, Mostafa: 4005

Husser, Erica K: 7060, 9075

Hutchinson, Jasmin: 9130

Huth, Megan: 1395, 2285, 7010, 7185, 7915

Hutto, Lashawn D: 7255

Huxhold, Oliver: 4490, 7355

Huynh, Duong: 8015

Huynh, James: 4250

Hwang, Dabin: 3205, 8015

Hwang, In Jeong: 8015, 9140

Hwang, Jeongeun: 7425, 7430

Hwang, Jinseon: 7020

Hwang, Mina: 7375

Hwang, Min Jun: 7265, 7305

Hwang, Misun: 7295

Hwang, Phillip: 1510, 8015

Hwang, Seo-Yeon: 9060

Hwang, Sinwoo: 7185, 7295, 7330, 8010

Hwang, Ula: 7090, 7485, 8010, 8015, 9140

Hwang, Woosang: 2405

Hwang, Yeji: 7155, 7290, 7375

Hyde, Martin: 7375

Hyer, Madison: 3285

Hyson, Rosemary T: 3385

Hyun, JaeWon: 2210, 7055, 7300, 8015

Hyun, Jinshil: 2400, 4310

I

Iacob, Eli: 1025, 8010, 8015

Ibrahim, Stephanie: 1295

Idemudia, Erhabor S. S: 7405

Idler, Ellen L: 1080

Igarashi, Ayumi: 2210, 7440

Igarashi, Ayumi: 9020

Ignacio, Daniel: 7145

Igwe, Ivuoma: 3065

Ihara, Emily S: 2165, 4600, 7005

Ijima, Momoka: 7140

Ikebe, Kazunori: 7365

Ikeuchi, Tomoko: 7445

Ikhide, Afeo: 8010

Iktilat, Khalil: 2105, 2415, 7090

Ilardo, Joan L: 4075, 7540, 8010

Iloputaife, Ikechukwu: 8010

Ilugbo, Oluwakayode J: 4565

Im, Eun-Ok: 7305, 8005

Im, Sua: 7065, 7365

Im, Yunju: 7325

in der Schmitten, Jürgen: 7220

Incerti, Lisa: 8010

Infurna, Frank J: 4290, 8000

Ingle, Pilar: 1215, 3245, 9105

Ingleton Gold, Lori: 8010

Ingram, Zalen: 7545

Ingvalson, Stephanie: 7300

Inker, Jenny: 7010

Inoue, Megumi: 2265

Inoue, Shigeru: 7345

Inouye, Sharon K: 2505, 8000, 8010

Intraligi, Valerio: 7070

Intrator, Orna: 3115, 8010, 9095

Ionescu, Elena: 7075

Ip, Edward: 8000

Ipiankama, Sampson C.: 2220

Irani, Elliane: 3300, 4215, 4335, 7335

Irbık, Dilare Ecenur: 4325

Irimia, Andrei: 8000

Irshaid, Hisham: 3230

Irving, Kate E: 7010

Isaac, Alexis: 7255, 7375

Isaac, Gregory: 7000

Isaacowitz, Derek M: 1045, 7460, 8000

Isaacson, michal: 3200

Isales, Carlos M: 8010

Ishikawa, Tomoki: 7250

Ishioka, Yoshiko: 7260

Iskandar, Mark H: 8000

Islam, Jessica: 8015

Islam, Shahidul: 7410, 8000

Islas huertas, Gabriela: 2555

Issac, Gregory: 3220

Isvan, Nilufer: 3190

Ito, Kae: 7300

Ito, Kae: 8010

Ito, Keitaro: 7140

Ito, Tomoko: 8000

Itoh, Sakiko: 4590

Iveniuk, James D: 1105, 4285

Ivenuik, James: 2295

Iwagami, Masao: 8000, 8010, 8015

Iwami, Taku: 2230

Iwasaki, Michiko: 7365, 7400

Iyer, Sandhya: 8005

Izekenova, Aigulsum: 7445

Izekenova, Assel: 7445

Izumi-Mishima, Yuna: 8015

J

Jaber, Sausan: 7265

Jablonski, Rita A: 7280

Jablonski, Rita: 8000

Jablonski, Rita A: 8015

Jacelon, Cynthia: 7250

Jack, Clifford R: 1200

Jacklin, Kristen: 1160, 2170, 4045

Jackson, Dylan B: 3235

Jackson, Emma: 2030, 8010

Jackson, Eva M J: 7010

Jackson, Faith M: 8005

Jackson, Kathryn: 1265, 4120

Jackson, Kelley L: 2045

Jackson, Lynn: 7440

Jackson, Phronie: 7050

Jackson, Phyllis D: 3165

Jackson, Yolanda: 7200

Jackson-Sagredo, Andrea: 7430

Jacob, Alexandra E: 4570

Jacob, Christine: 4155

Jacobs, Jeremy M: 1155

Jacobs, Karen: 4345

Jacobs, Leslie: 7010

Jacobs, Lindsey: 7310

Jacobsen, Juliet: 4575

Jacobson, Mireille: 1250, 8010

Jacobus, Claire: 7150

Jaen, Joce: 4540

Jagasia, Krish: 8015

Jahn, Jacob: 4560

Jain, Felipe A: 3025, 8000, 8005, 8010, 8015

Jain, Jennifer: 8010

Jain, Shalini: 7190

Jak, Amy: 8010

Jakiela, Laura W: 4205, 7390

Jamali, Ali Akbar: 7205

James, Bryan D: 1200

James, Dara L: 1440

James, Everette: 7515

James, Nick: 7375

James, Peter: 2400, 8000

James, Sarah-Naomi: 1105

James, Shirley A: 8000

Jamieson, Scott: 7435

Jamil, Md Shafayat: 7190

Janevic, Mary: 7390, 8010

Jang, Aeji: 8010

Jang, Haejin: 8015

Jang, Heejung: 8000, 9015

Jang, Jeong H: 7305

Jang, Jinwoo: 2045

Jang, Jiyoon: 7350

Jang, Joy Bohyun: 1105, 8010

Jang, Seyeon: 7415

Jang, Soeun: 7390, 7415, 8010, 8015

Jang, Soong-nang: 7005, 7135, 7540

Jang, Sua: 7300

Jang, Yunseo: 7295

Jang, Yuri: 3325, 4465, 7210, 7305, 7355, 7390

Janicki, Matthew P: 1395, 4265

Janke, Robert: 7485

Jankowski, Catherine: 7085, 7910

Jansen, Taylor: 1075, 1320, 3170, 7170, 7210, 7245, 7305, 7375, 7530

Janssen, Leah M.: 8015

Janssens, Lien: 8010

Jantea, Rachel: 8005

Jao, Ying-Ling: 3035, 3220, 7465, 7500, 9030

Jaros, Maria: 1150

Jarrín, Olga F: 3205, 8005, 9045

Jarrott, Shannon E: 1240, 1505, 7105, 7470, 7545, 8005

Jasuja, Guneet K: 2085, 8005

Jayakody, Oshadi: 8015

Jazwinski, S. Michal: 4330

Jeamjitvibool, Thanakrit: 4225

Jeffery, Molly M: 7485

Jehu, Deborah A: 9080

Jen, Sarah: 2525, 7110, 7220

Jenkins, Emerald: 4265

Jenkins, Molly: 9145

Jennette, Kyle J: 3355

Jennings, Lee A: 1510, 2445, 7415, 8000

Jennings, Stephen: 1470

Jensen, Christine J: 7010

Jeon, Dojin: 8015

Jeon, Jaemin: 7470

Jeon, Pureum: 4525

Jeon, Sangha: 7355, 8010

Jeon, So Yeon: 3235, 8015

Jeong, Ansuk: 9140

Jeong, Chung Hyeon: 8010

Jeong, Daeun: 7295

Jeong, Haelim: 7500

Jeong, Jihyeong: 7090, 7475, 8010

Jeong, Seohye: 8005

Jeong, Seonghyun Ellin: 9110

Jesdale, William: 7255

Jesmin, Syeda S: 7145

Jespersen, Brooke V: 7135

Jessee, Lisa: 3195, 4360

Jester, Dylan: 7370

Jetson, Raymond A.: 7440

Jezak, Samantha T: 4347

Jha, Sonya: 1435

Jhaveri, Kavya: 7095

Ji, Linying: 7535

Ji, Minghui: 4505

Ji, Molin: 2160

Ji, Xuefan: 4135, 7415

Ji, Yuwen: 8000

Jia, Heling: 9045

Jia, Shuli: 9150

Jia, Xueqing: 7240, 9150

Jiakponnah, Nwanyieze Ngozi: 4330

Jian, Bo: 4315

Jiang, Da: 1350, 4300, 7375, 7395

Jiang, Jing: 7265

Jiang, Lin: 8015

Jiang, Nan: 7015, 7095

Jiang, Nisi: 4347

Jiang, Xia: 9150

Jiang, xiangxiang: 4265

Jiang, Xiaoqian: 7015

Jiang, Xiaoyan: 9150

Jiang, Yanan: 8000

Jiang, Yanping: 4070, 4505, 7090, 9025

Jiang, Yingyue: 1115

Jiang, Yuling: 7225

Jiang, Yun: 7295

Jiao, Hongmei: 7090

Jimenez, Andreina: 2440

Jimenez, Daniel: 1055, 7035, 7205

Jimoh, Itopa I: 1150

Jin, Bora: 8010

Jin, Guoping: 4530, 7090, 7260, 7435

Jin, Liangyi: 7365

Jin, Yichen: 4295

Jin, Ying: 7525

Jin, Ying: 8015

Jin, Yuanyuan: 1125

Jing, Bocheng: 7140

Jing, Mingrui: 7015

Jing Weinberg, Astrid: 9110

Jingyuan, Xu: 7010

Jo, Ryan: 8000

Johansson, Patrik: 7440

Johns, Lily: 3100

Johns, Michelle: 8010

Johnson, Abree: 2125

Johnson, Alexa: 8010

Johnson, Alisha H: 8005

Johnson, Benjamin: 7005

Johnson, Charles: 1105

Johnson, Chloe: 7245

Johnson, Daniel: 7150

Johnson, Dayna: 7465

Johnson, Elma: 7010

Johnson, Florence: 1510, 2375

Johnson, Ian M: 1420, 4165

Johnson, Jack: 4265

Johnson, January: 2170

Johnson, Jeremy: 8015

Johnson, Julene K: 2135, 7260

Johnson, Kim G: 7010

Johnson, Kimson E: 3175, 4580, 7200

Johnson, LaShaune P: 3140

Johnson, laurinda: 2260

Johnson, Madelyn: 2285

Johnson, Peace: 9000

Johnson, Rhea: 1505

Johnson, Sallie: 1135

Johnson, Shannen: 8005

Johnson, Shawn M: 9005

Johnson, Sterling C: 1200, 8015

Johnson, Ted: 1080, 7225

Johnston, Carrie: 1490

Jolin, James: 1465

Jolin, Pierre: 1465, 8015

Jon, Emily: 8005

Jones, Anna: 8010

Jones, Brian D: 7225

Jones, Christine D: 7485, 7930

Jones, Emily: 7005

Jones, Hannah: 8015

Jones, Katie: 4090

Jones, Kayleigh: 7010

Jones, Kelly: 8005

Jones, London B: 7225

Jones, Mary: 1275

Jones, Megan D: 7120

Jones, Randy A: 8015

Jones, Raymond: 2245, 3180, 7245

Jones, Richard N: 1225, 2000, 2505, 3380, 4540, 7435, 8010

Jones, Samia: 4190

Jones, Tessa: 4430

Jones-Cobb, Brittany R: 8010

Jones-Locklear, Jennifer: 1020

Joo, Jin hui: 1345, 2310, 9105

Jordan, Jacqueline: 3040

Jordan, Meg: 8000

Jor’dan, Azizah J: 8010

Joseph, Joshua: 3285

Joseph, Preetha: 7240

Joseph, Sarah: 2445

Joshi, Amol: 4065

Joung, Hyesong: 8015

Jovalekic, Aleksandar: 7415

Joyce, Jillian: 7095

Ju, Catherine H: 2240, 3100

Juang, Yu-Chen: 7220

Juarez-Briggs, Melida A: 8010

Juckett, Lisa: 7545

Judd, Suzanne E: 7260

Judge, Katherine: 2030, 7230, 7915, 8010

Judon, Kimberly: 8010

Jun, Hee-Jin: 7495

Jun, Jiweon: 7230

June, Andrea: 3150

Jung, Dukyoo: 8010

Jung, Grace: 8005

Jung, Hailey: 2525, 7355, 7535

Jung, Hyejin: 8010

Jung, Hye-Young: 1385, 7250, 7540, 9090

Jung, Inhye: 7145, 7355, 7395, 7470

Jung, Misook: 7220, 7325, 7425, 7430

Jung, Wi Hoon: 7470

Jung, Wonkyung (Kelly): 3205, 7395

Jungo, Katja: 7250

Junker, Tom: 7035

Jurczak, Michael J: 8000

Jurgens, David: 3100

Juris, Jill: 3260, 4600, 7105, 7175, 7215, 7470, 7545, 7920, 8015

Jurkowski, Elaine T: 7340, 7920

Just, Allan: 8015

Just, Danielle: 3040

Justice, Jamie: 8000

Justice, Jen R: 8000

Justin, Moore: 4525

Jutkowitz, Eric: 1010, 1430, 2260, 3020, 3075, 3190, 7185, 7200, 7300, 7530, 7930, 8005

Jylha, Marja: 1080

K

Kabalan, Mayssan: 1495

Kabel, Allison M: 7065

Kabua, Philmar M: 7005

Kachal, Josefa: 7135

Kadiu, Teuta: 4105

Kado, Deborah M: 3065

Kado, Deborah: 8005

Kaeberlein, Matt: 8000

Kahkoska, Anna: 8005

Kahsay, Eskira: 2105

Kahya, Melike: 1255, 7495

Kaiser, Ronald: 3215

Kaiser-Grolimund, Andrea: 7250

Kaizi-Lutu, Marc A: 1510

Kajiyama, Bruno: 7230, 7305, 8000

Kajiyama, Claudia: 7305

Kakuta, Momoho: 4195, 7365

Kalender-Rich, Jessica L: 1485

Kales, Helen C: 8015

Kalesnikava, Viktoryia A: 3100, 9035

Kalir, Daphna M: 4205

Kalousova, Lucie: 1140, 3175, 7055

Kalter, Steve: 7300

Kalu, Michael: 7300

Kalyani, Rita: 7315

Kamalyan, Lily: 8010

Kamaraj, Heera: 8000

Kamath, Vidyulata: 2535

Kamer, Angela: 3110

Kamide, Kei: 7365

Kamis, Christina: 7260

Kamiyamasaki, Etsuyo: 7330

Kämpfen, Fabrice: 9150

Kan, Ge Lin: 7295

Kan, Junpeng: 7090

Kane, Alice: 7090

Kane, Esther: 1015, 7185

Kane, Kevin: 8000

Kang, Ashley J: 8010

Kang, Bada: 2210, 7325, 7395, 7520, 7525

Kang, Chaeryon: 4585

Kang, Donghun: 3025, 4420

Kang, Hun: 2210, 7055, 7300, 7375, 8015

Kang, Jaewon: 2445

Kang, Jee eun: 1445, 2360

Kang, Ji Young: 7275

Kang, Jihee: 8005

Kang, Jinkyung: 4450

Kang, Lin: 3160

Kang, Min Jeoung: 8015

Kang, Minji: 7395

Kang, Miseon: 3325, 7470

Kang, Naixin: 8015

Kang, Ning: 4255

Kang, Sarah Sunghye: 3020, 7495, 8005, 8010

Kang, Seerat K: 7460

Kang, Seokjo: 2277

Kang, Suk-Young: 9030

Kang, Sung Hye: 1405

Kang, Sunny: 7515

Kang, Yu Jin: 3040

Kannan, Lakshmi: 7225

Kanter, Jeremy: 4360

Kao, Hsueh-Fen S: 2175

Kao, Lihan: 9140

Kaplan, Celia: 9115

Kaplan, Daniel: 8000

Kaplan, Jordan: 7420

Kapoor, Alok: 2500, 7410, 7485

Kappler, Caitlin B: 1470

Karabukayeva, Aizhan: 1150

Karachaliou, Angeliki: 8000

Karagiannis, Tanya T: 8015, 9155

Karakida, Maki: 7395

Karakose, Selin: 1120, 1265

Karakullukçu Erdem, Sima: 3310

Karasik, David: 1520

Karasik, Rona J: 2145, 3120, 4035, 7340, 7920

Karayanidis, Frini: 3235

Karel, Daniel: 1190

Karg, Patricia: 4305

Karimi, Zahra: 7060

Karki, Dinesh: 7390, 7475

Karlin, Nancy J: 7280

Karliner, Leah: 9090, 9115

Karlsen, John E: 4200

Karlsson, Ida: 4030

Karmacharya, Isha: 4465

Karmarkar, Amol: 8015

Karmarkar, Shriya: 7290

Karr, Daniel W: 1305

Karra, Vijay Kumar M: 7510

Karsky, Sam: 7465

Karvonen-Gutierrez, Carrie A: 1110, 4460, 7345

Kashiwabara, Kosuke: 8000

Kashiwagi, Kimikazu: 8000

Kashyap, Divya: 8005

Kaskie, Brian: 3150, 4390, 7070, 7185, 7930

Kasl-Godley, Julia: 2240

Kaslow, Angela "Jackie" A: 1370

Kaspar, Roman: 7380

Kastner, Keri: 1385, 7370

Katagiri, Keiko: 7365, 7395, 7925

Katsumata, Asako: 7160, 7405

Katz, Amy: 7010, 7910

Katz, Benjamin: 4007, 7020, 7095, 7200, 8000, 8005, 8010, 8015

Katz, Mindy j: 2400, 4310

Kauffman, Timothy L: 4255

Kaufman, Brystana: 4430

Kaufman, Jennifer E: 2395, 3385

Kaufman, Joel: 2400, 7040

Kaufman, Sarah: 9100

Kaufmann, Christopher N: 1295, 1475, 2035, 3235, 4385, 8015

Kaup, Migette L: 2080

Kaushal, Navin: 4560, 9130

Kavanagh, Grace: 7350

Kawabata, Kan: 8000

Kawachi, Ichiro: 1225

Kawai, Hisashi: 7225

Kaye, Jeffrey: 1280, 4200, 7335, 7545

Kaye, Lenard W: 7170, 8005, 8010, 8015

Kayser, Jay: 3175, 7395

Kayser, Kylie: 7435

Kazakoff, Megan: 8010

Ke, Jin: 7035

Keach, Nancy: 9010

Keage, Hannah: 3235

Keefe, Bronwyn: 2490, 4075, 4345

Keefe, Francie: 4185

Keefe, Francis: 7205

Keisari, Shoshi: 1330, 4205, 4235, 7410

Keiser, Brennan: 4535

Keith, Amy: 2330

Keith, NiCole: 3085, 7415

Keith, Verna: 4140

Keleher, Jennie: 4475

Kelekar, Uma: 1355, 4030, 4190, 4440, 4560

Kelle, Nadja: 4490

Keller, Hallie: 7220, 7410

Keller, Michelle: 7485, 8010

Kellett, Kathy: 1315, 7360

Kelley, Heather: 4085, 7215, 7305

Kelley, Jessica A: 1405, 2020, 3117, 3285, 8005

Kelley, Lynette: 2540, 3115, 7280, 7350

Kelley, Shaelyn R: 7545

Kelly, Christopher M: 4550, 7130, 7515

Kelly, Heather: 7330

Kelly, Kathleen: 2450

Kelly, Madeline: 7480

Kelly, Megan: 7140

Kelly, Megan M: 7300

Kelly, Megan: 7905

Kelson, Mark J: 2120

Kemeny, Betsy: 9080

Kemnitz, Joseph W: 7045

Kemp, Candace L: 1275, 2160, 4550

Kemp, Elizabeth: 4205

Kemp, Elyria: 8005

Kendzor, Darla E: 1510

Kenj Halabi, Dima: 7430

Kennedy, Anna: 7500

Kennedy, Katherine A: 2225, 3040, 4010, 4550, 9005

Kennedy, Meaghan A: 3115, 7300

Kennedy, Richard: 1060

Kennedy, Teri: 3255, 8000

Kennerley, Clare L: 7435

Kenney, Jamesia: 7325

Kennison, Robert F: 7460

Kenny, Anne: 1160

Kenny, Brooks: 9010

Kenowsky, Sunnie: 7010

Kent, Erin E: 4450, 8000, 8005

Kenyon-Pesce, Lisa: 9090

Keough, Katie: 8010, 8015

Kermel-Schiffman, Ile: 1090

Kern, Kristopher C: 7220

Kern-Lyons, Calista: 3295

Kerssens, Chantal: 7225

Kessler, Dorothy: 7340

Kessler, William: 8010

Ketcher, Dana: 1160, 4045

Ketel, Christian: 3355

Key, Jamie: 4240

Key, Kent: 2515

Key, Mickeal N: 7265

Keys, Matthew T: 7345

Keysor, Julie: 8015

Kezios, Katrina: 2390, 8000, 8005, 9120

Khalaila, Rabia: 8010

Khaleghzadegan, Salar: 2045

Khalidi, Sarah: 4140

Khalil, Abdelouahed: 7315

Khan, Karim: 7225, 7905

Khan, Maham: 8000

Khan, Najah A: 7320

Khan, Nasrin: 1060

Khanpour, Sahar: 4590

Khasawinah, Sarah: 7185

Khatri, Rekha: 8015

Khattar, Jayati: 2545

Khatun, Asifa: 1005

Kheirbek, Raya Elfadel: 1190, 2125, 2485, 3235, 4600, 9005

Kheirbek, Raymond: 2125

Khera, Nina: 8000

Khobragade, Pranali Y: 4540

Kholodovsky, Eric: 4560

Khoo, Eliza: 4370

Khor, Ern Chern: 4420

Khorgamphar, Mohammad: 8000

Khorob, Kvitoslava: 3360, 4270

Khosla, Sundeep: 2137

Khurana, Amit: 8010

Kibildis, Samuel W: 8005, 8015, 9025

Kidd, Lori: 4600, 7340, 7920

Kiefer, Lea: 1470, 7030

Kieffer Blass, Elana: 9125

Kiel, Douglas P: 7130

Kietzman, Kathryn: 8000, 8010

Kiflom, Rebecca: 8010, 8015

Kilaberia, Tina R: 7015, 7250

Kilcrease, Amanda K: 4015

Kilduff, Courtney: 2310

Killian, Emily: 2135, 8000

Killian, Meredith: 8015

Killian, Tim S: 7475

Kim, Agnes Jihae: 3075, 8000

Kim, Andrew: 8015

Kim, Andy Jeesu: 8005

Kim, Bo: 7270

Kim, Boah: 8010

Kim, Boeun: 3345, 4125, 9005

Kim, Bo-Kyung Elizabeth: 4250

Kim, Bo-Kyung Elizabeth: 4250

Kim, Bomgyeol: 2210, 7055, 7300, 8015

Kim, BoRin: 4070, 4185, 7495, 8005, 8010

kim, byungju: 8010

Kim, Changhyun: 8010

Kim, Chang-O: 7005

Kim, Chang-O: 7135

Kim, Da Eun: 8015

Kim, Dae Hyun: 1340, 4090, 9000, 9070, 9080, 9085, 9105, 9135, 9145

Kim, Dahee: 1055, 3030, 7090, 7160, 7225, 7380

Kim, Daniel: 9150, 9155

Kim, Dongmi: 7305

Kim, Dongmi: 8005

Kim, Dongwook: 7295

Kim, Doyoung: 8005

Kim, Elissa W: 1415

Kim, Eric S: 7115

Kim, Eunbea: 4195, 4315, 7040, 9140

Kim, Eunbee: 7475

Kim, Eunice: 9145

Kim, Eunji G.: 9000, 9085, 9105, 9145

Kim, Eunjung: 4335, 7390

Kim, EunKyo: 7185, 7295, 7330, 8010

Kim, Eunkyung: 7400

Kim, Gee: 1485

Kim, Giyeon: 3325, 7035, 7080, 7090, 7115, 7165, 7330, 8000, 8005

Kim, Gwang Suk: 7090, 7145, 7225, 7245, 7265, 7300, 7375, 8005

Kim, Haeun: 7035

Kim, Hae-Young: 9060

Kim, Ha-Neul: 3215

Kim, Hansol: 8010

Kim, Heekyung: 8015

Kim, Heewon: 7500

Kim, Hyejin: 8000

Kim, Hyejoong: 7080

Kim, Hyun: 1345

Kim, Hyun Jung: 7475, 7545, 7910

Kim, Hyun-Jun: 7355, 7535

Kim, Jaehyun: 7265

Kim, Jaeyoon: 7290

Kim, Jeehoon: 7295, 7395, 8015

Kim, Jennifer L: 7295

Kim, Jeong Eun: 7335

Kim, Jeong Lan: 3235

Kim, Jeonghun: 7255

Kim, Jeongwoon: 7435, 8005, 9130

Kim, Jeung Hyun: 3370

Kim, Ji Chul: 9040

Kim, Jichan: 4335, 7390

Kim, JinShil: 7220

Kim, Jiwon: 7435

Kim, Jiwon: 8015

Kim, Jiyeon: 3315

Kim, Jiyoung: 8010

Kim, John: 8015

Kim, Joohyun: 7230

Kim, Ju Hyun: 7390

Kim, Jueon Aaron: 7085

Kim, Junesun: 9060

Kim, Jung-Nim: 7220

Kim, Junhyoung: 7265, 7305, 8000, 8015

Kim, Keun You: 8015

Kim, Kyeongmo: 3325, 7260, 8010

Kim, Kyeongwon: 4250

Kim, Kyung Soo: 3355

Kim, Kyungmin: 2025, 3185, 3335, 4485, 7115, 7185, 7230, 7335, 7355

Kim, Laura: 9025

Kim, Layoung: 7090, 7145, 7225, 7245, 7265, 7300, 7375

Kim, Manjun: 7295

Kim, Maryanne: 8010

Kim, Meeryoung: 8000, 8015

Kim, Miji: 7325, 8010

Kim, Min Hee: 2020, 3050

Kim, Minjin: 8005

Kim, Minzae: 1510

Kim, Nahyun: 7395, 7925

Kim, Namhee: 7090, 7225, 8010

Kim, Nam-Hee: 8000

Kim, Namhyun: 8005

Kim, Natalie: 8000

Kim, Nicholas J: 8000

Kim, Ocksim: 7375

Kim, Patricia: 7540

Kim, Sangkyu: 4330

Kim, Sara: 1345

Kim, Sein: 7230

Kim, Seohyeon: 8015

Kim, Seokmin: 1075, 7070, 7375

Kim, Seon: 7260

Kim, Seoyoun: 1195

Kim, Seulki: 3020

Kim, Sewon: 7300

Kim, Sion: 7395

Kim, Sohee: 7420, 7905, 8005

Kim, Sohyun: 3260, 4320

Kim, Soo Hyun: 7380, 7430, 7495

Kim, Soo Yeon: 8010

Kim, Soohyun: 8005

Kim, Steffi: 3165, 4350

Kim, Suhyun: 7100, 7295

Kim, Sun Ju: 7060, 8010

Kim, Sun Kyung: 7475

Kim, Sunjung: 9125

Kim, Suyoung: 3265, 4195

Kim, Tae: 2200

Kim, Taekyung: 3025, 3075

Kim, Tina: 1345

Kim, Yeoeun Kate: 7085

Kim, Yeonwoo: 4580

Kim, Yijung: 3340

Kim, Yongseop: 4580, 7265, 8010

Kim, Yoojee: 2310

Kim, Yoojee: 9110

Kim, Yoon Chung: 1395, 2150, 8000, 8005

Kim, Young Sun: 9060, 9065

Kim, Young Woo: 8005

Kim, Yuhoung: 8015

Kim, Yulri: 1120, 4315, 9140

Kimball, Patricia: 1085

Kimerling, Rachel: 2085

Kim-Godwin, Yeoun Soo: 7005, 7400

Kim-Knauss, Yaeji: 1330

Kimpel, Christine: 3355

Kimron Greenblatt, Lee: 3360

Kimura, Yuya: 9020

Kinariwala, Kush: 2065

King, Angelique: 3295

King, Barbara J: 4425, 7495

King, Caroline: 4500

King, DJ: 7195

King, George A: 8010

King, Jessica: 2225

King, Kelsey: 1160

Kinkade, Emily: 8015

Kinney, Jennifer: 8005

Kinzig, Elizabeth: 2485

Kiosses, Dimitris: 4010

Kirby, Mackenzie: 7455

Kirch, Matthias: 4360, 4390, 7250

Kirch, Matthias: 9070

Kircher, Julie A: 8015

Kirk, Greg: 7545

Kirkegaard, Allison: 3010

Kirkpatrick, Tara: 7380

Kirkwood, David: 7370

Kirsch, Jaclyn: 8015

Kirsch, Julie A: 4215

Kirwan, Jack B: 8010

Kiselica, Andrew: 9070

Kishimoto, Kyoko: 2145

Kisley, Michael A: 2095

Kissam, Stephanie: 7385

Kisselburgh, Hannah: 9130

Kitamura, Koji: 7225

Kitamura, Satomi: 7250, 7465

Kitchens, Katherine: 8010

Kittle, Krystal R: 4140

Kitt-Lewis, Erin: 1310, 2485, 4480, 8010, 9075

Kitzman, Dalane: 2245

Kitzman, Dalane: 9145

Kivipelto, Miia: 1290, 3030

Kivisäkk, Pia: 7415

Kivowitz, barbara: 2500

Kiwak, eliza: 7030

Kiyohide, Fushimi: 9020

Kizilhan, Jan: 8005

Kizony, Rachel: 7210

Klaiber, Patrick: 1480, 7035

Klatt, Maryanna D: 2135

Klayn, Rami: 7060

Kleckner, Amber: 7130

Klein, Kelly: 8010

Klein Druyan, Adar: 1515

Kleinstein, Steven H.: 8000

Klenczar-Castro, Brittany: 2010

Klepacz, Laura: 1175, 7380

Klepin, Heidi D: 1255

Kleszynski, Keith: 7415, 8000

Klinedinst, Jennifer: 7290, 7495, 8005

Klinedinst, Tara: 1510, 2245, 2445

Kling, Mitchel A: 4570

Klinger, Christopher: 7340, 7920

Klomhaus, Alexandra: 8010

Klonover, Eyal: 2105

Klopack, Eric: 8015

Klug, Jenna: 1520

Kluger, Benzi M: 3165

Knebl, Janice: 2065

Knell, Tammy A: 7410

Knibbeler, Marjolein: 7250

Knight, Christy: 4425

Knight, Cynthia M: 1050

Knight, Erik L: 8015

Knight, Joseph: 2010

Knight, Julie: 7500

Knodt, Annchen: 7025

Knopman, David: 2535, 7325, 7495

Knowles, Justina E: 8015

Knox, Sara: 8005, 9035

Ko, Clifford: 7500

Ko, Eunjung: 1400, 2475, 4170, 8005

Ko, Fred: 4425, 8010

Ko, Jisook: 8015

Ko, Jong: 7475

Ko, Kwangman: 7085

Ko, Soohyeon: 7335

Kobayashi, Erika: 2120, 7265

Kobayashi, Hiroyuki: 8015

Kobayashi, Lindsay: 1495, 2000, 3380, 4060, 4455, 4540

Koch, Christina: 8005

Koestner, Sophie A: 7065

Koffer, Rachel E: 4050

Koffski, Maya J: 2330

Koga, Hayami K: 8000

Koglin, Norman: 7415

Koh, Vanessa Jean Wen: 2435

Koh, Yen-Chun: 8015

Koh, YoungBin: 7005, 7080

Kohlbrenner, Briana: 1215

Kohler, Susan: 9145

Kohler, Thibault: 4580

Kohlhepp, Bayard: 9020

Kohli-Hailovski, Tal: 4355, 7175

Kohn, Ella: 8000

Kohon, Jacklyn: 3280

Koike, Teruhiko: 7480

Koirala, Binu: 7380, 7430, 7495

Kojima, Misao: 7265

Kolachalama, Vijaya B: 3127

Kolanowski, Ann: 3220

Kolawole, Francis: 7300

Kole, Debarati: 2290, 8000

Kolluri, Surya: 2115

Kolpin, Lola: 8005

Komalasari, Renata: 8000, 8005

Komiyama, Jun: 8000, 8015

Kondrikov, Dmitry: 8010

Kong, Chunyan: 3020

Kong, Dexia: 7305, 8015

Kong, Eun-Hi: 8000, 8015

Kong, Jian: 7435

Kong, Jooyoung: 4520, 7290

Kong, Lingsong: 9070

Konkle-Parker, Deborah: 2140

Kononowech, Jennifer: 1300

Konopka, Adam: 1387

Koo, Katherine: 7485

Kooij, Dorien: 1480

Kooshiar, Hadi: 8005

Kopera-Frye, Karen: 2290

Koren, Chaya: 1130, 4195

Koren, Omry: 2105

Koren, Yael: 7435

Korenman, Sanders: 3385

Korfage, Ida J.: 7220

Korlat, Selma: 3145

Kornadt, Anna E: 1210, 1480, 3360, 4270

Koroni, Hamid R: 8015

Koroukian, Siran: 7220

Kosar, Cyrus M: 2000, 7540

Kositsawat, Jatupol: 4025

Koss, Catheryn: 1215, 7015, 7920

Kossowska-Kuhn, Dorota: 4420, 8005

Koszalinski, Rebecca S: 8015

Kota, Veda: 2320

Kotal, Ashwin: 4280

Kotian, Bhumi Jagadish: 7340

Kotterman, Amy: 1300

Kottorp, Anders: 4215

Kotwal, Ashwin A: 2190, 3050, 4020, 4070

Kovaleva, Mariya: 7125, 9000

Kowalchick, Kalei: 4480

Kowalski, Rebecca: 8015

Kozakowski, Kevin M: 7320

Koziel, Michele: 1300

Kozlov, Elissa: 2320, 7370

Králová, Josefína: 9070

Kraal, A Zarina: 3380

Kraemer, John D: 7530

Kraft, Malissa: 3005, 7195

Kramer, Arthur: 7265

kramer, Benjamin: 4200

Kramer, Philip A: 2245, 2470, 8005

Kramer-Kostecka, Eydie: 4020

Kraschnewski, Jennifer L: 4480

Kratz, Anna: 4195

Kravchenko, Julia: 1235, 4010, 4460, 7185

Kravljaca, Nikolina: 7325

Kreider, Amanda: 4260

Kreider, Consuelo M: 2290, 2445

Krein, Sarah: 4450

Kremen, Sarah: 8010, 9020

Kremer, Ian N: 4160

Krendl, Anne: 1095, 2360

Kreuzinger, Isa-Marie: 7020, 7265

Krick, Katelynn E: 2460

Kriebernegg, Ulla: 2140, 7445, 7490

Kristensen, Katrine L: 7345

Kritchevsky, Stephen: 1075, 2470, 4275, 4295, 8000

Krok-Schoen, Jessica L: 2220

Kropp, Denise J: 7340, 7920

Krueger, Katie: 3255

Krumm, Isabella: 3150

Kruse, Lance: 8005

Ku, Hok Bun: 8005

Ku, Nai-Wen: 7090

Ku, Te-Lien: 1035, 1365, 2135, 2445, 8005

Ku, Vivian: 8010

Kuan, Yun-Han: 3035, 7465

Kuang, Huijing: 8015

Kube, Erin R: 2240

Kubiak, Jenna: 3170

Kubota, Sayaka: 7330

Kubzansky, Laura D: 8000, 8005, 9155

Kucharska-Newton, Anna: 4450, 7345

Kuchel, George A: 4025, 4473, 4495, 7345, 8000, 8005, 8010, 9090

Kuehnemann, Chisaka: 8005

Kuffel, Randall: 7255

Kuglin Jones, Ann: 2255

Kukull, Walter: 8000

Kullgren, Jeffrey T: 4360, 7250

Kulminski, Alexander: 8005

Kumapayi, Peace: 7300

Kumar, Amit: 8015, 9045

Kumar, Ashish: 8015

Kumar, Manish: 7040

Kumar, Sanjana: 2320

Kumar, Vivek: 7190

Kumfor, Fiona: 4470

Kummerfeld, Erich: 9070

Kung, Alina: 8000, 8010

Kung, Po-Jen: 2480

Kunicki, Zachary J: 2000, 2505, 3380, 4015, 4540, 7290, 7435, 8000, 8010, 8015

Kuniholm, Mark: 8015

Kunik, Mark E: 1035, 4265

Kunkel, Suzanne: 7170

Kunzmann, Ute: 8000

Kuo, Chia-Ling: 4025, 4495, 8000, 8010

Kuo, Fang-Lin: 7475

Kuo, Jasmin: 7130

Kuo, Tony: 7225, 7905

Kuo, tsuann: 7150, 8000

Kuo, Yong-Fang: 1070, 4365, 7305, 7535, 8000, 8005, 8010

Kupferschmidt, Anthony: 1325

Kura, Niharika: 8005

Kuribayashi, Shihomi: 7225

Kurita, Kayoko: 8000

Kurth, Jordan D: 8005

Kurth, Maria: 2280

Kurtz, Chava: 7145

Kurzhiparambil, Leya T: 3280

Kusmaul, Nancy: 1145, 1395, 2150, 4395, 7220

Kuspinar, Ayse: 8000

Kusters, Cynthia DJ: 1475

Kutner, Jean S: 3375

Kutney-Lee, Ann: 8005

Kutz, Alexander: 8015

Kuzmik, Ashley: 2180, 4380, 8015

Kwait, Jennafer: 2140

Kwak, Jung: 2130, 7305, 8015

Kwak, Min Ji: 8000

Kwak, Minyoung: 7470, 7925, 8015

Kwan, Crystal: 1115

Kwan, Ngai: 3205

Kwan, Rick Yiu-cho: 4180

Kwapis, Janine: 2137, 7190, 8010

Kwiatek, Susan: 7410

Kwoh, C. Kent: 7500

Kwok, Alex Pak-ki: 4180

Kwok, Amy: 8005

Kwok, Eric Lun Yiu: 7255

Kwok, Ian B: 3340

Kwok, Jennifer H: 2040

Kwok, Jojo Yan Yan: 4300, 7085, 7395

Kwok, Yu Hon Kenneth: 1030

Kwon, Eunsun: 7275, 8015

Kwon, Jenny: 4345, 8010, 8015

Kwon, Oh Sung: 8000

Kwon, Simona C: 1345

Kwon, Soonhyung: 3325, 7305

Kwon, Sooyoung: 7090, 7245, 7265, 7300, 7375

Kwon, Suyoung: 4125

Kwong, Katherine: 4035

Kyaw, Kaela: 3295

Kyeremeh, Ebenezer: 7360

Kyriacou, Corinne: 3040

L

La, Jennifer: 8000

Létourneau, Dominic: 2235

Lévesque, Marie-Hélène: 2235, 4150

Löckenhoff, Corinna: 3275, 4300, 7380

López-Lorenzo, Karla Daniela: 3355

Laager, Rahel: 8015

Lac, Andrew: 7395, 7505

Lach, Helen W: 7225, 7905

Lachman, Margie E: 4000, 7115, 7200, 8010

Lachs, Josh: 8005

Lachs, Mark: 1170, 1215, 2325, 3275

Lackmeyer, Abbe: 7170

LaCroix, Tracy: 8005, 8010

Ladda, Shawn: 7305

Ladiges, Warren: 1520, 7480

Ladner, Daniela Patricia: 7185, 8000

Laflamme, Maeva: 7455

Lafontant, Kworweinski: 3030, 8005, 8010

Lafountain, Olivia: 2525, 7110

LaFrance, Marianne: 8000

Lagisetty, Pooja: 4450

LaGorio, Kara: 7265

Lagrotteria, James: 7085, 7910

Lahlou, Yusuf: 8000

LaHue, Sara C: 4107

Lai, Chao-Qiang: 4460

Lai, Daniel W. L.: 1215, 1225, 2260, 2315, 2325

Lai, Jin-Shei: 4015

Lai, Jocelyn: 1045, 3270

Lai, Patrick Ho Lam: 1425, 2530

Lai, Vincent D.: 9015

Lai, Wenhua Zoey: 2050, 3010, 7230

Lai, Wen-Hua Zoey: 1060

Laird, Lance: 4535

Laitman, Adam: 7500

Lakomy, Martin: 2405

Lam, Gigi: 4180

Lam, Julia: 8000

Lam, Justin W: 7295, 7410

Lam, Michael: 8000

LAM, Tommy L H: 4455

Lamar, Melissa: 8005

Lambrou, Nickolas: 1160

Lamontagne, Marie-Eve: 8005

Lamp, Amanda H: 3295

Lampe, Nik M: 1315, 4220, 8005

Lamura, Giovanni: 7070

Lan, Yue: 7265

Landi, Francesco: 8010, 8015

Landreville, Philippe: 8000

Lane, Sandi J: 2090, 7075

Lane, Scott: 8005

Lanford, Tiera: 4430

Lang, Frieder R: 1330

Lang, Lang: 7245

Langa, Kenneth M: 4540

Langdon, Jacqueline M: 9045

Lange-Maia, Brittney: 8005

Langendoerfer, Kaitlyn: 8010

Langston, Emily M: 4080

Langworthy, Benjamin: 1430, 3020, 3075, 7300, 7530

Lankowski, Ava: 8015

Lanternier, Paula: 9015

Lanza, Dalila V: 3155

Lanza, Stephanie: 7180

Lapacek, Brian T: 8010

Lapane, Kate: 2555, 7255, 7415

Lapidus, Jodi: 1460, 7315

Lares, Gabino: 8005

Large, Edward W: 9040

Lariviere, Matthew: 9100

Larkin, Jennie: 4008

Larkin, Marni: 7140

Larkina, Marina: 7305

LaRoche, Ashley: 8015

LaRoche, Dain: 4005

Larouche, Julie: 4105

Larson, Kelsie: 4045

Larson, Peter: 7465

Larson, Scott: 7305

Larson-Smith, Yvonne: 8005

Laskin, Grant: 8015

Laskow, Thomas: 2510

Lasseigne, Brittany N: 3397

Lassell, Rebecca KF: 2380, 3085, 4525, 7415

Latham, Nancy: 2395, 8015

Latham-Mintus, Kenzie: 1190, 1415, 2485

Lathrop, Laura: 1440

Latimer, Abigail: 7360, 7475

Lau, Bobo: 9085

Lau, Bobo Hi Po: 1385, 4180, 7205

Lau, Fung Yee: 4585

LAU, Gary K. K.: 1225

Lau, Matthew Zhi Yuan: 3215

Laurienti, Paul J: 1075

Laurita-Spanglet, Jessie: 8000

LaValley, Michael: 1070

Lavayen, Susana: 7455

Lavretsky, Helen: 4535

Lavu, Siva: 8010

Law, Chancen: 8000

Law, Makenna E: 1160, 2310, 7295

Law, Stephanie: 1385

Law (Jang), Jeein: 4030, 4440, 4560, 7320

Lawrence, Carly W: 2085

Lawrence, Jennifer: 2115

Lawrence, Joycelyn: 8000, 8015

Lawrence, Karen A: 7035, 7450

Lawrence, Katharine: 4170, 8015

Lawrence, Molly: 7305, 8015

Lawrence, Reagan W: 7330

Lawrence, Sean: 8005

Lawson, Denise: 7185

Lay, Jennifer C: 1350, 7375

Lazarus, Rachel: 7025

Le, Qun: 8000, 8015

Le, Thai Minh: 7390

Leach, Susan: 7140

Leahy, Suzanne: 7330

Leal, Kelly: 7265

Leary, Natalie: 7205

LeBlanc, Raeann G: 4135, 7110, 8000

LeBrasseur, Nathan: 4107, 7190

LeCouteur, David: 7240

LeCroy, Madison N: 3155

Lee, Ah Rim: 7325, 7425, 7430

Lee, Angella: 7310

Lee, Ann: 2440

Lee, Aryn: 7350, 7540

Lee, Beatrice: 8010

Lee, Boram: 8015

Lee, Chae Man: 1385, 2185, 4135

Lee, Chaiwoo: 3250, 4410

Lee, Chanam: 1135, 3125

Lee, Chang Dae: 7335

Lee, Chioun: 3285

Lee, Chiyoung: 7010, 7500

Lee, Christopher S: 2255

Lee, Dahyeon: 7295

Lee, Dahyeon: 7425

Lee, Daniel: 8010

Lee, David R: 3290

Lee, Dayoung: 2120

lee, Donghyun: 9065

Lee, Ellen: 1295

Lee, Eunju: 4485, 7230

Lee, Eunseo: 7295

Lee, Gina: 1065, 4520, 7290

Lee, Gyeonghee: 7235

Lee, Haerim: 7220

Lee, Hanna: 8015

Lee, Harold H: 8010

Lee, Hayan T: 9070

Lee, Hazy H.Y.: 2315

Lee, Heashoon: 8010

Lee, Hee: 1365, 7295

Lee, Hee Yun: 8000

Lee, Heejin: 7325

Lee, Heeyoung: 8000, 9050

Lee, Hochang Benjamin: 1345

Lee, Hyangkyu: 7060

Lee, Hye Soo: 4080, 7380, 8005

Lee, Hyo Jung: 3325, 7145, 7355, 7395, 7470

Lee, Hyunjoo: 8015

Lee, Hyunjoo: 9065

Lee, Ian: 8015

Lee, Jae Jun: 7145, 7225, 7300, 7375

Lee, Jaesung: 8000

Lee, Jane Y: 2530

Lee, Jane H: 7460

Lee, Jean Mui Hua: 2150, 3135

Lee, Jeesun: 7380

Lee, Jennifer: 4227, 7315

Lee, Jeong Eun: 1120, 4315, 7040, 9140

Lee, Jeongeun: 7430

Lee, Jeonghwa: 7005, 7400

Lee, Jeongmin: 7125

Lee, Jessica: 4415

Lee, Ji Hyun: 4285, 7295, 7395

Lee, Ji Yeon: 8000

Lee, Jiha: 7120

Lee, Jihwan: 7005, 7135

Lee, Jinkook: 2000, 3010, 3380, 4280

Lee, Jin-kyung: 8005, 8015

Lee, Jin-Moo: 1355

Lee, Jongwon: 7435

Lee, Joohyun: 4060

Lee, Joy: 4240

Lee, Juhyeong: 4270, 7090, 7165, 7390

Lee, Jung Ah: 8015

Lee, Jung-Ah: 7220, 7475, 7545, 7910

Lee, Jungha: 8015

Lee, Jungjoo: 7305, 8000, 8015

Lee, Jun-Young: 8015

Lee, Kangeun: 8000, 8015

Lee, Kathy: 1020

Lee, Kayoung: 7125

Lee, Kayoung: 8010

Lee, Knoo: 9070

Lee, Kristine H: 8005

Lee, Kyuho: 7470

Lee, Kyung Hee: 2210, 7525, 8000

Lee, Kyungmi: 7430, 8005

Lee, Kyungun: 7115

Lee, Kyuri: 7330, 8000

Lee, Lewina O: 3330

Lee, Lewis H: 7500

Lee, Linda: 7230

Lee, Mi Jung: 2445

Lee, Mina: 7085

Lee, Miu Yee: 1385

Lee, Othelia O: 4180

Lee, Pei-An: 1110, 7295, 7465, 7495

Lee, Pin-Shiuan: 7020

Lee, Pui: 8000

Lee, Rebekka: 2310

Lee, Rokbit Sanghee: 7065, 7365

Lee, SangA: 7185, 7295

Lee, Sei: 1025, 7140

Lee, Shinduk: 1300

Lee, Shinduk: 9025

Lee, Shwn-Jen: 7370

Lee, Soohyoung Rain: 9100

Lee, Soomi: 1475, 2230, 2385, 4050, 4215, 7365, 7390, 8000, 8005

Lee, Su-In: 2137

Lee, Sun Ah: 2280

Lee, Sung-Jin: 4210

Lee, Sun-Kyung: 7185

Lee, Sunmin: 7210

Lee, Tae-Ho: 8005

Lee, Traci: 1215

Lee, Vincent W. P.: 1225

Lee, Wai Tong: 2455

Lee, Wongyeong: 8010

Lee, Yen-Chin: 9050

Lee, Yen-Han: 1235

Lee, Yeonjae: 8015

Lee, Yeonjung: 2140

Lee, Yeonjung: 3020, 4210, 7005, 7035, 7080

Lee, Yeonjung (Jane): 1065

Lee, Yeon-Shim: 7160, 7375, 7415

Lee, Yoojin: 3370, 7510

Lee, Yookyong: 7300

Lee, Youjung: 2340, 4185

Lee, Young-Shin: 7495

Lee, Younho: 8015

Lee, Yura: 8000

Leedahl, Skye N: 4600, 7105, 7215, 7545

Leeman, Jennifer: 7105

Lees Haggerty, Kristin: 1085, 9135

Leff, Bruce: 1270, 2480

LeGars, Abigail K: 8010

Leggett, Amanda N: 1060, 3010, 4015, 7230, 8005, 8010

Leggett, Christopher: 8000

Lehmberg, Lisa J: 2165

Lehning, Amanda J: 4150, 7090, 8010

Lehr, Marcus: 4460

Lehtisalo, Jenni: 1290

Lei, Lianlian: 4390

Lei, Tingyu: 4055

Leis, Aleda: 2000, 4460, 7345

Leiser, Scott F: 2417

Leist, Anja: 1105, 8000

Lekhak, Nirmala: 2500

Leland, Natalie E: 1270, 8000, 8010

Leme, Daniel: 8000, 8010

Lemire, Emma: 9080

Lemus, Armando: 8015

Lenchik, Leon: 7165

Leng, Iris: 2245

Leng, Sean X: 3160

Lennon, Danielle B: 3005, 4350

Lent, Jaia: 7470

Lentz, Jeffrey G: 4135

Leo, Hubert: 3105

Leon, Lydia: 7345

Leonard, Claire: 7065, 7900

Leong, I tek: 1400

Leontovich, Natalia: 7330

Lepetit, Anne: 8015

Lepinteur, Anthony: 1210

Lepola, Noah: 8010

Lepore, Michael J: 1395, 2150, 2335, 2465, 3117, 4135, 4160, 8000, 8005

Lerma, Sofia: 7190

Lerner, Edie: 7290

Lerner, Emily: 4360, 7290

Lero, Christi M: 7485, 8000

LeRolland, Emily: 9040

Leshchyk, Anastasia: 8005, 8015, 9155

Lesser, Janna: 3355, 7255

Leszczynski, Jennifer: 8010

Letts, Elisa: 4445

Leung, Cindy: 7325

Leung, Daniel Dick-man: 4180

Leung, Dara Kiu Yi: 1235, 4455, 7255

Leung, Hannah Shui-Wah: 8015

Leung, Jasen Kin-Fung: 7145

Leung, Rachel Hoi Laam: 2250

Leung, Vania: 3240

Leutwyler, Heather C: 7130, 7290, 9060, 9090

Levan, Darius: 7290

Levasseur, Mélanie: 2235, 2545, 4150

Levasseur, Melanie: 2235

Leveille, Suzanne: 4560

Levenson, Robert W: 1275, 4085, 4515, 7290

Levin, Gali: 2105

Levin, Jason: 7265

Levin, Michael: 7215

Levitan, Eric: 1230

Levitt, Daniel: 8005

Levkoff, Sue: 1090

Levy, Becca R: 2370, 8000, 9035, 9125

Levy, Cari: 1130, 1145, 1300, 1435

Levy, Natalie: 7285, 7925

Levy, Sorah: 2350, 4460, 8005

Levy-Storms, Lené: 3020, 8010, 9145

Lewinson, Terri: 4150

Lewis, Charity T: 7290

Lewis, Gabrielle: 7265

Lewis, Jordan P: 2420, 3165, 4350

Lewis, Jordan P: 8005

Lewis, Joshua Christian Julian: 2155

Lewis, Lauren: 2135

Lewis, Mickal D: 7010, 9030

Lewis, Nathan: 4490, 4515

Lewis, Stuart: 1085

Leykum, Luci: 8010

Li, Aaron: 8000, 8005

Li, Ai-Tzu: 7080

Li, Boyuan: 8000

Li, Caiwen: 8015

Li, Chenxi: 7345

Li, Chien-Ching P: 2300, 7100

Li, Chihua: 4060

Li, Chihua: 4540, 7365

Li, Chih-Ying: 8010

Li, Daniel: 8015

Li, Guoyan: 7265

Li, Hao: 1460

Li, Haolin: 8005

Li, Haoru: 7025

Li, Hongyu: 7430

Li, Huan: 8015

Li, Huixian: 8005, 8015

Li, Isadora: 8015

LI, Jessica J.: 1225

Li, Jiajia: 9150

Li, Jianan: 3020, 7290, 7470, 7925, 8010

Li, Jiaxin: 7090

Li, Jiaying: 4385, 7390

Li, Jie: 1385

Li, Jing: 2185, 7535

Li, Jingyi: 4180

Li, Jingyu: 8015

Li, Juan: 3340

Li, Juan: 4330

Li, Juan: 7015

Li, Juan: 7030

Li, Juan: 7430

Li, Juan: 8000

Li, Junxin: 2480, 4010, 4070, 4335, 4385, 7390, 9075

Li, Ke: 4070, 4185, 7495, 8000

Li, Kelin: 8005

LI, Kin-Kit: 8015

Li, Lihua: 2185

Li, Ling: 4370

Li, Liping: 7225, 7905

LI, Lun: 2140, 2315, 8010

Li, Lydia W: 7395

Li, Meiyi: 7260

Li, Meng-Hao: 2265

Li, Mengting: 9105

Li, Mengze: 8005

Li, Niying: 2190, 4580

Li, Qian: 7040

Li, Qiwei: 7380, 7430

Li, Rosemary: 8010

Li, Ruoxuan Rosalyn: 2230

Li, Shan: 7240

Li, Shaojie: 7225

Li, Shuang: 4365

Li, Shuang: 7300

Li, Shumin: 1245

Li, Shuning: 9020

Li, Shuzhuo: 7470, 7900

Li, Sizhe: 8005

Li, Tao: 7410, 8015

Li, Tianyuan: 3275

Li, Tianzi: 1245

Li, Wanling: 3075

Li, Wei: 8015

Li, Wenhan: 4370, 7310

Li, Wenjing: 1060, 7230

Li, Wenjun: 8000, 8015

Li, Xiang: 8010

Li, Xiaoang: 4275

Li, Xiaoli: 7250, 7380, 7430

Li, Xiaoli: 8005

Li, Xiaoyouxiang: 8005

Li, Xingyu: 8015

Li, Ya-Hsin: 2300

Li, Yanping: 8010

Li, Yaru: 8000

Li, Yawen: 2305, 7305

Li, Yaxin: 7410

Li, Yezhen: 2000, 3010

Li, Yiang: 2295

Li, Yibo: 9105

Li, Yingru: 7380

Li, Yixia: 7205, 7255

Li, Yuanyuan: 7315

Li, Yucan: 7320

Li, Yue: 4345

Li, Yuehua: 8005

Li, Yuekang: 7145

Li, Yuelin: 7150, 8000, 9050

Li, Yuhang: 1070

Li, Yun: 7125

Li, Zhen: 7350, 7930

Li, Zheng: 8005

Lian, Zheng (Noah): 1140, 7055

Liang, Bo: 7300, 7430

Liang, ChangPu: 7085, 7435

Liang, Jersey: 2120

Liang, Jiaming: 2540, 4565

Liang, Jie: 7030

Liang, Siqi: 3090

Liang, Siqi: 8000

Liang, Xiaoxuan: 7300, 7430

Liang, Yanjing: 4180, 7100

Liang, Zhen: 3160

Liao, Gerald Y: 1520, 7480

Liao, Hsiao-Wen: 2350, 7525, 8015

Liao, Shuyao: 3275

Liao, Yanru: 3370

Liao, Yo-Jen K: 3035, 3220, 7465, 7500

Libby, Julia B: 7145

Libera, Theresa L: 8010

Libermann, Towia A: 2505, 8000

Libon, David J: 4570

Lichtenberg, Peter: 2115, 3130, 7190, 7310

Lieber, Sarah B: 4525

Liebermann, Erica: 7105, 7215

Lifshitz, Rinat: 1090

Lighthall, Nichole: 7375

Likhterman, Miriam M: 7350

Lim, Angelica: 7205, 7915

Lim, Arum: 7380, 7430, 7495

Lim, Donovan: 4370

Lim, Hayun: 8000

Lim, Junyub: 3370

Lim, Kimberly: 4475

Lim, Lionel: 7410

Lim, Wee Shiong: 7120

Lim, Wen Huey: 2435

Lim, Xinru: 8005

Lim, Ye-Ji: 7100

Lim, Youngmi: 8015

Lim, Zhi Zhen: 8005

Lima, Julie: 1435

Limani, Merita: 1070

Lim-Soh, Jeremy: 1205, 3325

Lin, Asenath: 2350

Lin, Carol: 7485, 8000

Lin, Chia Jou: 7060

Lin, Feng-Chang: 1235

Lin, Frank: 1075, 1200

Lin, Gloria: 8005

Lin, Haiqun: 3205, 9045

Lin, Hongmei: 1330, 3360, 4300

Lin, Hui-Yi: 2300

Lin, I-Fen: 1280, 3050, 4085

Lin, Jason: 9070

Lin, Joshua: 1340, 4090, 7510

Lin, Ju-Ping: 7165

Lin, Li-Hui: 8010, 8015

Lin, Molly Zhixuan: 1330, 2250, 4300

Lin, Natong: 1110

Lin, Nelson: 3155

Lin, Rose: 8005

Lin, Sabrina: 1185

Lin, Shih-Yin: 1435, 2015, 2380, 3205, 4015, 7185

Lin, Shiqi: 2275

Lin, Tzu-Yu: 7445

Lin, Xin Yao: 3025, 4080, 8015

Lin, Yan: 7305

Lin, Yi: 7190

Lin, Yunann: 7340, 7470

Lin, Yuqian: 7010, 7125

Lin, Zhiyong: 2040, 9105

Lin, Zhuoer: 1160, 2040, 2105, 3200

Lind, Lisa: 2240

Lindauer, Allison: 1015, 1165, 7415

Lindberg, Brian W: 1450, 2215, 2355, 3210

Lindberg, Daniel: 1085, 3300

Linden, Wolfgang: 4490

Lindenberger, Ulman: 4050

Linder, Jeffrey: 8000

Lindsay, Rebecca: 7390

Lingard, Ayaka: 3215

Lingler, Jennifer: 4045, 7335

Link, Jessica: 1315

Linker, Alexandra: 8000

Linker, Maddison: 7200

Linscott, Robert: 7390

Linsky, Amy: 4090

Lipitz, Sarah: 8015

Lipsitz, Lewis A: 1255, 3117, 3235, 7025, 7130, 7295, 7465

Lipton, Richard: 2400, 4310, 7535, 8015

Lipworth, Loren: 9030

Lisa, Jenna: 8005

Littleton, Tenesha: 2340

Liu, Aihong: 4370, 7310

Liu, Amy: 4520

Liu, Bian: 1270, 8000

Liu, Carissa: 8015

Liu, Chiung-ju: 2290, 7265, 8010

Liu, Christopher: 1465, 8015

Liu, Chunyu: 7040

Liu, Chunyu: 8000

Liu, Chunyu: 8015

liu, Dan: 7240

Liu, Darren: 2090, 2300, 8000

Liu, Ethan: 8015

Liu, Eunbee: 8000

Liu, Fangyu: 1155, 4495

Liu, Hang: 9100

Liu, Hao "Howe": 4255

Liu, Haoyuan: 7015

Liu, Ho Kan: 8000

Liu, Hui: 1125, 2295, 3050, 7255

Liu, Hui: 7320

Liu, Jianfang: 7125, 7185

Liu, Jiaru: 3225

Liu, Jingwen: 1240

Liu, Jingyuan: 7335

Liu, Jinyu: 9115

Liu, Julianna: 2505

Liu, Jun: 1340

Liu, Junxian: 8000, 8010

LIU, Karen P. Y.: 4455

Liu, Keyao: 2125

Liu, LiFan: 1290

Liu, Longjian: 7015

Liu, Marian: 1085

Liu, Mengjing: 4200

Liu, Mengshi: 7130, 7185, 7200, 7210, 7255, 7260, 7300, 7305

Liu, Mengting: 2305

Liu, Mengyu: 1515

Liu, Michelle: 7390, 9075

Liu, Peiran: 4495

Liu, Pi-Ju: 8010

Liu, Qiyun: 7385

Liu, Rui: 7345

Liu, Ruotong: 4505

Liu, Ruotong Mona: 3110, 4505, 7485, 8010

Liu, Shao-Hsien: 7255

Liu, Sofia: 4070, 4385, 9075

Liu, Tianyin: 8000

Liu, Wanru: 7010, 7185, 7930

Liu, Wen: 3220, 4015, 4125, 7330, 8000

Liu, Xiaodong: 4030

Liu, Xiaojuan: 1340, 4090, 7510

Liu, Xueyan: 8005

Liu, Yaqing: 7300, 7410, 7930

Liu, Yi: 7160, 7225

Liu, Yichun: 7330

Liu, Yifan: 2480

Liu, Yiming: 8005

Liu, Yin: 4520, 7435

Liu, Ying: 2120, 4435

Liu, Yingjia: 8000

Liu, Yiwen: 1105

Liu, Yu: 7435

Liu, Yuanhao: 7015

Liu, Yuetong Toria T: 8015

Liu, Yujun: 1505, 4030, 7040

Liu, Yunrui: 8010

Liu, Zeyu: 7455, 7915

Liu, Zuyun: 7240, 9150

Liu-Ambrose, Teresa: 7225, 7905

Livingston, Melvin: 1085

Livne, Ofir: 1500, 4390

Lizarazo, Maria Jose: 8010

Llaneza, Danielle H: 7220

Lo, Grace: 8010

Lo, Jeanie: 7350, 7930

Lo, On-Yee: 1110, 1255, 7435, 7495

Lo, Yi-Chen: 8015

Lo, Ying-Ying: 7515

Lober, William B: 4200

Locascio, Joseph: 2310

Locher, Julie L: 1465

Loeb, Susan J: 2485

Loerzel, Victoria: 7425, 8005, 9100

Logan, Jeongok: 8010

Logue, Caroline: 4450

Logue Cook, Rachel N: 7120, 7310

Lohman, Matthew: 8000

Lohmar, Sabine: 1440, 7255

Loibl, Cäzilia: 3285

Loizos, Maria: 9080

Lok, Kris Yuet Wan: 7085

London, Andrew S: 2085

Londono, Valeria: 3340

Long, Armari: 7225

Long, Monica: 2205

Long, Yanan: 4200

Longo, Valter: 3267

Lopez, Bruna C: 1425

Lopez, Janet: 1055, 7090, 7160, 7225, 7350, 7380, 8005, 9100

Lopez, Ruth Palan: 8005, 8015, 9000, 9095

Lopez, Ruth Palan: 1145

Lopez Blanco, Jose David: 7210

Lopez-Ortega, Mariana: 2060

LoPilato, Danielle: 8000, 8015

Lopis, Joyce A: 8005

Lor, Maichou: 8015

Lord, Justin: 3365

Lord, Marie-Michele: 2235

Lord, Sébastien: 4150

Lords, Hannah: 8005

Lorenzo, Erica: 8000

Losin, Elizabeth: 8000

Lou, Vivian Weiqun: 1030, 2350, 7350, 7360, 7365, 7495, 8000

Lou, Yifan: 1140, 2265, 4325, 7005, 7325, 7375, 8000, 8005, 9115

Loui, Psyche: 9040

Louie, Patricia: 4440

Louis, Elan: 7415

Louis, Sandro: 8010

Louis-Charles, Willencia: 4080

Loutet, Katrina: 7225, 7905

Louzoun, Yoram: 2105

Lovasi, Gina S: 2400

Love, Sheltoria: 8000

Lovence, Keisha: 2245

Lowenthal, Julie: 2075

Lu, Hongzhou: 4055

Lu, Jinxia: 7300

Lu, Kevin: 4265

LU, Lucy Xi: 4455

Lu, Minjie: 8005

Lu, Peiyi: 7360, 7365, 7495, 8010

Lu, Shiyu: 4370, 7150, 7220, 7925

Lu, Shu-Chen: 7370

Lu, Sihan: 7515

Lu, Siyao: 8000

Lu, Thomas: 7440

Lu, Xiaomin: 8010

Lu, Xuan: 8015

Lu, Yangfan: 7435

Lu, Yi: 3275, 4300, 7380

Lu, Yifei: 1200

Lu, Yijun: 7325

Lu, Yvonne: 7010, 7265, 7910, 8010

Lubetsky, Sara: 9145

Luborsky, Mark: 8010

Lucantoni, Davide: 7070

Lucas, Jennifer: 7515

Lucas-Wright, Anna: 4250

Lucca, Kaitlyn: 1015

Lucci, Katherine: 7230

Lucey, Brendan: 4385

Luchetti, Martina: 1120, 1265, 1445, 1480, 4360

Luciana, Monica: 8015

Ludwig-Borycz, Elizabeth: 7325

Lui, Fan: 4180

Lui, Shirley Shuk-Kuen: 7395, 7440

Luk, Sau Man Angela: 1030

Lukkahatai, Nada: 4010

Lum, Terry Yat Sang: 1030, 1135, 1235, 4455, 7255

Luna, Kenya: 4440, 8015

Lunak, Azylen M: 2330

Lundebjerg, Nancy: 2215

Lundquist, Tessa: 1440

Lundy, Theresa L: 9055

Lunetta, Kathryn L: 8005

Luo, Aaron: 8010

Luo, Huabin: 3110, 7485, 8010

Luo, Jing: 1265

Luo, Juan: 7045

Luo, Jun: 2380

Luo, Junyuan: 3270

Luo, Lan: 9000, 9085, 9105

Luo, Lan: 9135

Luo, Lan: 9145

Luo, Li: 7410, 8015

Luo, Minxia: 4270

Luo, Tianyi: 8005

Luo, Xiao: 3025

Luo, Yan: 2545

Luo, Yanan: 4055, 4455, 7010

Luo, Yuan: 2460

Luo, Zhehui: 1500

Luong, Gloria: 2095, 3270

Luong, Thuc: 7380

Lusardi, Michelle M: 7140

Lusignani, Maura: 8015

Lussier-Therrien, Marika: 2235

Lustbader, Wendy: 3055

Lustria, Mia Liza A: 1285

Lute, Sara M: 8000

Luth, Elizabeth: 2320, 7220, 8000

Luth, Elizabeth: 8010, 8015

Luther, Brenda: 1300

Lutsey, Pamela L: 4385, 7545, 8010, 8015

Lv, Gang: 4265

Lv, Yuebin: 8005

Lwi, Sandy J: 4085

Ly, Ka: 7000

Ly, Timothy K: 3185

Lydon, Lizzy: 1165, 9085

Lynch, Colleen: 7265

Lynch, Connor: 8005

Lynch, David: 1220, 1235

Lynch, Delanie: 7165

Lynch, Grace: 7390

Lynch, Jennifer: 9100

Lynch, Karla: 7465

Lynch, Kathleen: 1090

Lynch, Scott M: 8000

Lynn, Freda: 2135

Lynn, Rachel: 2255

Lyons, Heidi A: 2295

Lyons, Karen S: 3375, 4485, 7335, 8000, 8015

Lyons, Kathleen: 7300

Lysandrou, Apollonia E: 7375

Lyu, Wangyizhuang: 7305

M

Ma, Carol Hok Ka: 7235

Ma, Chenjuan: 2440, 8000, 8010

Ma, Chunxuan: 8005

Ma, Da: 7480

Ma, Gu: 3275

Ma, Guoyong: 2305

Ma, Guoyong: 8015

Ma, Jessica: 4430

Ma, Jinhui: 7225, 7905

Ma, Kaiting: 7015

MA, Lina: 8005, 9085

Ma, Maggie S. L.: 2135

Ma, Mingyue: 8005

Ma, Qing: 7090, 7315, 7320

Ma, Runze: 8015

Ma, Stefan: 2230

Ma, Tongyu: 9020

Ma, Winkie: 1265

Ma, Yuan: 1245, 7090, 9040, 9155

Ma, Yuxuan: 2305

Ma, Zhenxuan: 8005

Ma, Ziqi: 8005

Müller, Beat: 8015

Mabry, Marshall: 8010

MacArthur, Barbara: 8000

Macaulay, Susan: 8000

MacConell, Leigh: 8010

MacConnachie, Lauren: 1110, 4460

MacDonald, Catherine: 1005

Macdonald, Karen C: 7275

MacDonald, Stuart: 7115

MacDougall, Sarah: 7365, 7400

Mace, Ryan A: 1160, 2310, 7295, 9110

Macedo, Luciana: 7225, 7905

Macedo, Sabrina: 8005

MacGillivray, Beth: 8000

Machiano, Joseph: 7225

Machida, Masaki: 7345

Maciel, Alvaro: 8005

Mack, Laurin J: 9140

MacKay-Brandt, Anna: 8015

Mackenstadt, Darby: 8015

MacKenzie, Kelsey: 8015

MacKenzie Greenle, Meredith: 8000

Mackintosh, Siobhan: 8015

MacNeil, Andie: 7180

MacPherson, Andrew: 2355, 2495

Madden, Ken: 4490

Madden, Kenneth: 7225, 7905

Maddow, Charles: 1085

Mader, Michael J: 8000, 8010

Madera, Sonia: 8005

Madison, Mali A: 8005

Madlock-Brown, Cherisse: 8010

Madrigal, Caroline: 1470, 4480, 9080

Maeda, Akari: 2210

Maestre, Gladys Elena: 8015

Magalhaes, Larissa: 8000

Magaziner, Jay: 7495

Magdalena Giovani, Élcio CC: 7300

Mage, Susanna M: 2390, 3300, 7085

Maggs, Jennifer: 7180

Magid, Kate: 1300, 8005

Magnusson, Dawn: 7140

Magruder, Kathryn M: 2085

Maguire, Teagan: 7485

Maham, Abeera: 9080

Maher, Brion S: 1510, 7040, 8015

Maher, Joanne: 9020

Mahfuza Haque, Mahi: 7290

Mahmood, Atiya: 1325, 2515, 3305

Mahmoud, Kafayat: 3195, 4580

Mahmoud, Nourhan: 7315

Mahmoudi, Elham: 1160, 3355, 7000

Mahone, Anais: 8000

Mahoney, Jeannette R: 2535, 4200

Mai, Chunyan: 8005, 8010

Mainieri, Tina: 3230

Mains, Douglas: 7540

Mair, Christina F: 2200

Mair, Christine A: 1240

Maitland, Daniel W M: 7355

Majette, Nadya: 7350, 7930

Majgaonkar, Hritik N: 8010, 8015

Majon-Valpuesta, Dolores: 2235

Mak, Felix: 2350

Mak, Jonathan Ka-Long: 2510

Mak, Wingyun: 7250, 7330

Makaroun, Lena K: 1085, 9140

Makela, Meghan J: 9080

Makita, Meiko: 1135

Makris, Una: 7120

Malani, Preeti: 4390

Malatyali, Ayse: 1055, 4450, 7160, 7380, 8005

Malcolm, Millicent M: 7410, 7525

Malecki, Kristen: 7260

Malette, Holly: 2240

Malhotra, Atul: 2035

Malhotra, Atul: 8015, 9030

Malhotra, Rahul: 1205, 2230, 2435, 3325

Malkowski, Olivia S: 2120, 8010

Maloney, Gabrielle: 4325

Malta, Jessica S: 8015

Manalel, Jasmine A: 2395, 3385

Mandelblatt, Jeanne: 1475, 7190

Mandl, Lisa A: 4525

M’Angale, Peter: 8005

Mangialasche, Francesca: 1290, 3030

Mangiardi, Rosemarie: 7075

Mangila, Jessica: 2275

Mangione, Kathleen: 8005

Mangone, Laura: 8000

Manheim, Chelsea: 8000, 9095

Mani, Sneha Sarah: 1495, 2000, 4060

Manini, Todd M: 1295, 1390

Mankowski, Robert T: 7190

Manly, Jennifer J: 3380, 7040, 7435

Mann, Ajit Singh: 4300

Mann, Frank D: 7255

Mann, Jim: 7205, 7915

Mannan, Masuma: 2300

Mannheim, Ittay: 2370

Manning, Innessa A: 7500

Manning, Kenneth: 1470

Manning, Lydia K: 1135, 3150

Manocha, Samiya: 7290

Manor, Brad: 1110, 1255, 7025, 7295, 7465, 7495, 8010

Mansfield, Kelly: 2030

Mansfield, Melissa: 7380

Mansfield, Tyler: 1460, 4275

Mansion, Natalie: 1260, 7095

Mansoor, Marrium: 7020, 7200, 7230, 8010

Mansourian, Farnoush: 8005

Manzo, Alma A: 7305

Manzour, Rezvaneh: 8000, 9050

Mao, Hui-Fen: 8010

Mao, Jieyu: 7195

Mao, Lingchao: 8015

Mao, Weiyu: 1400, 2475, 4170, 8005

Mao, Xi: 8015

Mao, Ziling: 2245, 4295

Mar, Amanda B: 1185

Marabini, Camilla: 2350

Marcano Maldonado, Patria: 7495

Marcantonio, Edward R: 2505, 7060, 8000

Marcatti, Michela: 8010

Marchant, Erik D: 8015

Marcos, Christine: 8005

Marcus, Cara: 3170

Marcus, Michael: 4595, 8000

Marcus, Zeve: 7155

Maredia, Asma: 8000

Mareverwa, Amos: 7430

Marfeo, Elizabeth: 1305, 2365, 7230, 7280, 7350

Margolis, Rachel: 1405

Marhánková, Jaroslava Hasmanová: 9070

Mariani, Diane: 8010, 9145

Marie, Boltz: 2180

Marincic, Michaela: 7465

Marini, Christina M: 2085

Marino, Francesca R: 1510

Marino, Victoria: 3330

Marinov, Aleksandar: 1325

Mark, Anthony: 8005

Marka, Nagaraju: 8000

Markham Risica, Patricia: 1465

Markides, Kyriakos S: 4290, 7465

Markland, Alayne D: 9115

Marks, Jane: 2205

Marnfeldt, Kelly: 1215, 3300

Marottoli, Richard: 7245, 7530, 8005

Marques, Brenda: 7535

Marquez, David X: 8005

Marr, Calum: 4205

Marroig, Alejandra: 3265, 4110, 7145, 8015, 9005

Marron, Megan M: 2000, 2245, 7345

Marrs, Sarah: 7340

Marsh, Kaetlin F: 7435

Marshall, Gillian: 3180

Marsiske, Michael: 4570, 8005

Marston, Hannah R: 3080, 9100

Marston, Holly: 8010

Martey, Ebenezer: 3370, 8000

Marti, C. Nathan: 7020

Martin, Allison: 7475

Martin, Cindy: 2170

Martin, Corby: 3267

Martin, Diane: 4345

Martin, F. Michael: 7150

Martin, Jennifer L: 1295

Martin, Peter: 1065, 4600, 7040, 7395, 7470

Martin, Seth S: 4385, 7545

Martin, Jr., Wesley T: 3005, 4350

Martindale, Jonathan: 7120

Martine, Shareck: 4150

Martinez, Alejandra: 2045

Martinez, Alejandra: 8015

Martinez, Alejandra: 9045

Martinez, Erica: 4445, 7410

Martinez, Iveris L: 2490

Martinez, Lusiana: 8000

Martinez, Madelyn: 7340

Martinez, Natalie A: 8010

Martinez, Pauline D: 2450, 3000

Martinez, Ryan: 7345

Martinez, Vanessa: 7325

Martinez Escudero, Jose: 7410

Martinez-Amezcua, Pablo: 1075, 4450, 7330, 7535

Martin-Peters, Tasha: 8000

Martinson, Melissa: 7485

Martinson, Wendy: 7305

Martire, Lynn: 1125

Martyr, Anthony: 2335

Marzetti, Emanuele: 8010, 8015

Marziliano, Allison: 2175

Masaki, Harue: 7215

Masaki, Kamal: 1065

Mason, Amara: 2340

Mason, Hannah Ç: 2325, 4080

Mason, Tyara R: 1470

Masoud, Sara S: 1135, 3355, 8000

Massa, Fernando: 7145

Massa, Franklin: 9005

Massihzadegan, Setarreh: 7030

Massimo, Lauren M: 1015, 7205

Massingale, Shermetria: 4135

Massizhadegan, Setarreh: 1415

Mast, Benjamin T: 7305, 7400, 7415, 7900

Masters, Julie L: 8010

Mastrostefano, Aglaé: 7065

Masui, Yukie: 7365

Masumoto, Shoichi: 8010

Masurkar, Arjun V: 7010

Matchar, David Bruce: 2435

Mathai, Paul: 7510

Mather, Mara: 8005

Mathers, Kate: 8010

Mathew, Jason R: 4200

Mathis, Lindsey M: 9115

Matlock, Daniel D: 2320, 2500

Matos-Moreno, Amílcar: 7200

Matovu, Schola N: 7085, 7915

Matsui, Hiroki: 9020

Matsumoto, Alvin M: 8000

Matsumoto, Hiroshige: 7440

Matsuo, Masafumi: 8015

Matsuzawa, Akemi: 8010

Mattek, Nora: 1280, 7475

Mattero, Joana: 9115

Matteson, Tyler J: 1330

Matthews, Alicia K.: 7100

Matthews, Jean M: 8000

Mattke, Soeren: 4435

Matz, Christina: 1425, 2050, 2530, 7500

Mauck, Erin: 7085

Mauer, Jess: 1335

Maurer, Jürgen: 4070

Mausbach, Brent T: 1295

Maust, Donovan T: 1010, 8005, 8010

Maxfield, Molly: 1440, 7010

Maxi, Andrea: 9100

Maxwell, Cathy A: 7075, 7295, 7545

May, Au Myat G: 8015

May, Jennifer T: 8000

May, Melissa: 4020

Maye, Jacqueline E: 8010

Mayeda, Elizabeth Rose: 4280

Mayer, Jamie: 4030, 7040

Maynard, Madison: 7375

Mayo, Nancy: 8000

Mays, Allison M: 8010

Maza Santibañez, Valentina: 9015

Maziarz, Lauren: 7370

Mazonson, Baylah: 8015

Mazor, Kathleen: 7485

Mazumder, Nikhilesh R: 7185

Mazzarella, Diana: 4070

Mazzola, Paolo: 1220

Mbakile-Mahlanza, Lingani: 7010

Mbam, Kingsley C: 7070, 7080

Mbanefo, Onyinye: 4565

Mbao, Mbita: 7455

M’Bra, Paule: 7415

McAlinden, Michael: 4205

McAllister, Denise: 4210

McArdle, Patrick: 2125

McAtee, Robin: 7015, 7920, 7935

McAuley, Edward: 1255, 7265, 8000

McBee-Black, Kerri: 7065

McBride Afonso, Tara: 1050

McCall, Stephen: 1495

McCall, Stephen: 3315

McCampbel, Jody: 1085

McCarthy, Ellen P: 9000, 9085, 9105, 9135, 9145

McCarthy, Michael J: 2170

McCarthy, Teresa: 2010, 7370

McCartney, Jenn: 4305

McCauliff, Jocelyn: 7410, 7910

McClellan, Angela: 4265, 7440, 7525

McClelland, Robyn: 7040

McClure, Leslie A: 2335

McClure, MaKenna: 2260

McClure, Nicole: 7375

McConeghy, Kevin: 7305

McConnell, Eleanor S: 2210

McConnell, Eleanor: 3040

McConnell, Eleanor S: 3135, 4075

McConnell, Eleanor: 4425

McConnell, Eleanor S: 7325, 8000, 9035

McConnell, Kelly M: 2220, 8000

McConnell, William R: 3050

McCormick, Mark: 2417

McCorvey, Stephanie A: 2350, 7085, 7495

McCoy, Brianah M: 8015

McCoy, Megan C: 1000

McCoy, Rozalina: 9075

McCracken, Courtney: 7110

McCrackin, Sarah: 1315

McCrae, Christina S: 4230, 8000

McCreedy, Ellen: 2055, 4475, 8005, 8015

McCullough, Megan B: 1300

McCune, Teagan: 7050

McDaniel, Miranda: 8015, 9050

McDannold, Sarah: 8005

McDermott, Cara L: 3065

McDermott, Kate: 2310

McDevitt, Ross A: 8000

Mcdonald, Anthony: 4435

McDonald, Claire: 7470

McDonald, Jerry: 1295

McDonald, Margaret: 1170, 7440, 7545

McDonough, Ian: 9040

McDougall, Graham J: 8005

Mcelfish, Pearl A: 7005

McFadden, Bridget: 8010

McFarland, Malcolm N: 8015

McFeeley, Brittany M: 7050, 7075

McGaffigan, Erin A: 1130, 3155

McGee, Jocelyn Shealy: 8005, 9105

McGee Lawrence, Meghan: 7245

McGovern, Justine: 2165, 4115, 9055

McGrath, Colleen B: 8000

McGready, John: 4450

McGregor, Casey: 7105, 7215

McGrory, Aileen: 2540, 7280, 7350

McGuinness, Bernadette: 4205

McGuire, Catherine: 4475

McGuire, Courtney: 7410, 7910

McGuire, Lisa C: 1395, 9010, 9125

McGuire, William: 7020

McIlvennan, Colleen K: 7085, 7910

Mcintosh, Nathalie: 2225, 3040, 4550

McInturff, Sophia: 8005

McIntyre, Cecily: 7410, 7920

McIntyre, Madeline: 7005

McKay, Michelle A: 4560, 8000

Mckay, Tara: 8005

McKendrick, Karen: 2185, 8000

McKenna, Kevin: 4565

Mckenzie, Barbara: 7000

McKibbon, Kristy: 7075

McKiddy, Sarah: 8010, 8015

McKinney, Lana P: 4210

McKinnon, Annette: 7230

McKoy, Khalil: 8005

McLaren, Jaye E: 2365, 3005, 7300, 7310, 8005

McLaughlin, Sara: 2260

McLees, Laura K: 7345

McLennan, Stuart: 7145

McLin, Maia: 2340

McManus, Caoimhe A: 2480

McManus, David: 2500, 7410, 7485

McManus, Regan: 7280

McMillan, Colleen: 7230

McMullen, Tara: 4160, 8005

McNamara, Maeve S: 9080

McNeish, Brendan L: 7425

McNichols, Christine C: 7300

McNicoll, Lynn: 7255

McPeek, Ashley: 2205

McPherson, Rachel: 2180, 3220, 4460

McPhillips, Miranda V: 4215, 4335

McQuown, Colleen: 1085

McShane, Michael: 9075

McSparran, Emerson: 2335, 4160, 7330, 9010

McTigue, Kathleen: 4535

Meadows, Jeff: 3060

Meah, Noah: 4015

Mecca, Adam P: 7530

Mecca, Marcia: 7030, 7410, 7920

Mechaber, Amanda: 7310

Medina, Jose T: 1425, 4325

Medina, Morgan N: 7270

Medina-Junge, Meira: 4235

Medlin, Laura M: 8010

Medya, Sourav: 4590

Meehan, Amy: 7050

Meeker, Daniella: 8015

Mehegan, Laura L: 7025

Mehl, Matthias: 8005

Mehmood, Yasir: 7320

Mehra, Eshan: 8000

Mehrotra, Sanjay: 7185

Mehta, C. Christina: 2395, 9080

Mehta, Raj: 9100

Mei, Haokun: 4505

Mei, Sharon: 2300, 7100

Meier, Madeline H: 4390

Meier, Tabea: 4515

Meier, Tabea: 4515

Meijer, Erik: 3380, 4280

Meisel, James: 7410

Meister, Melanie: 4230

Mejbaryan, Veranika: 7485

Mejia, Shannon: 3230, 4320, 4420, 7225, 7295

Mejia-Arango, Silvia: 8015

Melamud, Eugene: 7045

Melanson, Celia: 8005

Melekis, Kelly A: 4510, 8015

Mellow, Maddison L: 3235

Melms, Tobias: 7125

Melo, Ruth C: 7090, 7140, 7370

Melson, Jacob: 7280

Meltzer, David: 7430

Meltzer, Gabriella Y: 1090, 7040

Melville, Lucas: 8005, 8015

Memmer, Nicole: 3215, 7295

Memon, Ashraf: 8005

Mena, Emily: 1310, 3035

Menaker, Hannah: 8000

Menard, Alixe: 8015

Mendes de Leon, Carlos F: 1495, 1500, 7335

Mendez, Jennifer: 8010

Mendez, Xenia: 4260

Mendez Campos, Barbara: 2050, 7110, 7290

Mendez-Chacon, Ericka: 7090

Mendoza, Alonzo: 8010, 8015

Mendoza, Jose: 8000

Mendoza, Katie C: 1050

Mendoza, Nancy: 2340, 3055

Mendoza Ruvalcaba, Neyda: 7040

Menen, Tetla: 4240

Meng, Hailong: 8000

Meng, Han: 8000

Meng, Hongdao: 7040, 7190, 7305, 7395, 7420

Menin, Giovana: 7320

Menne, Heather L: 1395, 2260, 2335, 4160, 7085, 7330, 7540, 7915, 8005

Mentch, Heather: 7370

Meraiyebu, Ajibola Bart: 1430

Meraz, Rebecca: 8005, 9105

Merdjanoff, Alexis: 1090, 2250, 7345

Merin, Nathan H: 8010

Mero, Kiana: 8015

Merrick, Charlie: 4010

Merrilees, Jennifer J: 4085, 4515, 7290

Merten, Natascha: 1200, 2535, 8015

Mertz, Laurel: 1490, 7295, 7380

Mese, Patrick O: 7250

Messman, Brett: 8015

Meszaros, Katherine: 7470

Metcalf, Emily E: 7295, 8005

Metz, Regan: 8010

Metzler, Janna: 7345

Meuser, Aaron M: 3170

Meyer, Kylie: 3300, 7085, 8005, 8010

Meyers, David: 4430, 7485, 7540, 7930

Meynadasy, Melissa: 1050, 8010

Meynet, Stéphanie: 4150

Mez, Jesse: 1510

Mezuk, Briana: 2105, 3100, 4580, 7335

Mi, Hong: 1090, 2305, 7305, 8015

Mia, Hasib: 7415

Miao, Huali: 3075

Miao, Mary: 2545

Michaud, Francois: 2235

Michaud, Kaleb: 7120

Middleton, Addie: 9095

Middleton, Laura: 7225, 7905

Middleton, Lefkos: 1290

Miezah, Dennis: 8015

Migliaccio, John: 4595

Migneault, Deborah: 1385, 2185

Mihailovic, Aleksandra: 7530

Mike, Leigh Ann: 8010

Mikels, Joseph A: 2095, 7265, 7460, 8010

Mikus, Ellen: 9130

Milad, Elizabeth: 1265

Milani, Sadaf Arefi: 1070, 2005, 2345, 3370, 4560, 7465, 8000, 8015

Milberg, William: 7040, 8005

Mildrum Chana, Sofia: 4140

Miles, Declan: 7390

Milidonis, Mary: 7455

Milisen, Koen: 1060

Miljkovic, Iva: 7345, 7425

Millar, Courtney: 7130

Millar, Roberto J: 7515, 7930

Millenbah, Ashley: 1440

Miller, Amanda: 1265

Miller, Benjamin F: 2555

Miller, Christopher: 4090

Miller, Edward A: 1150, 2130, 2150, 3335, 4365, 7250

Miller, Eleanor: 7305

Miller, Emika: 8005

Miller, Jacqueline: 4260

Miller, Julie: 2530

Miller, Katherine: 1360, 2375, 2520, 3315, 4265

Miller, Kenneth L: 7140

Miller, Kyra A: 9110

Miller, Lindsay: 7345

Miller, Luke R: 4395, 7450

Miller, Matthew: 2245

Miller, Michael E: 1075, 2470

Miller, Patrick: 8010

Miller, Sara: 7180

Miller, Sarah J: 7180, 7505

Miller, Steven J: 8010

Miller, Steven: 9140

Miller, Vivian J: 7355, 7370

Milliard, Carrie: 7185

Milliken, Laurie: 3205

Mills, Corina I: 4250

Mills, Whitney L: 1060, 2225, 3040, 4240, 4550

Milman, Sofiya: 2557, 4495, 8000, 8015

Mimbs, Hope: 4245

Min, Deborah: 1345

Min Htike, Wai Yan: 8005

Minahan Zucchetto, Jillian: 7040

Minami, Christina: 8005

Minami, Kota: 7195

Mindlis, Irina: 1055, 4010, 4225, 7255, 8015

Minemoto, Sara: 7395

Miner, Brienne: 4335

Miner, Jennifer A: 4015

Ming, Yan: 8005

Mingo, Chivon A: 2550

Mino, Matthew: 7455

Minor, Bryan J: 4045

Minyo, Morgan J: 2285, 7010, 7915

Mion, Lorraine: 7545

Mirabella, Rosie: 8005

Mirin, Nicholas L: 8005

Mironi, Nava: 2390

Mirza, Mansha: 3265

Mirza, Raza M: 7340, 7920

Mishra, Ayati: 8000

Mishra, Shalini: 8015

Mishra, Swarup: 7190

Misra, Shalini: 4007

Misra, Sumi K: 2195, 7410

Missell-Gray, Rachel: 7145

Mistler, Lisa A: 2045

Mistler, Lisa A: 9045

Mital, Avani: 7045

Mitchell, Barbara: 7470, 7900

Mitchell, Chloe: 1400, 8010

Mitchell, Connor: 8015

Mitchell, Diane C: 7480

Mitchell, Jamie: 2515, 7190

Mitchell, Lauren: 4195, 7350

Mitchell, Uchechi: 2040, 7395

Mitlak, Hannah: 7440

Mitsova, Diana M: 4045

Mittelman, Mary S: 1400, 4170

Mittman, Brian: 4535

Mitzner, Tracy L: 1230, 2430, 4080, 8005, 9065

Miura, Chiharu: 7365, 7395, 7925

Miwa, Hiroyasu: 4590

Miyamae, Fumiko: 7440

Miyano, Yurina: 2210

Miyashita, Satoshi: 7195

Miyawaki, Christina E: 4265, 7440, 7525

Miyazaki, Atsuko: 9100

Mizrahi, Chanelle: 8010

Mizukami, Taeko: 2210

Mlecko, Maura R: 7200

Mlinac, Michelle E: 1050, 2240

Moberg, Ylva: 1095

Mock, Colleen: 7505

Moczygemba, Walter: 9005

Moeller, Eric: 7400

Moeller, Kelly: 1010

Moen, Phyllis: 1115, 1180

Moffitt, Chad: 8000

Moffitt, Terrie: 7025, 7115

Mogle, Jacqueline: 1175, 1480, 4050

Mogle, Jacqueline: 4310

Mogle, Jacqueline: 8000

Mogos, Mulubrhan F: 2255

Mohamed, Hodan: 9040

Mohamed, Rania E: 7170, 7220

Mohammed, Adam: 8000

Mohanty, Subhasis: 8000

Mohr, David: 2225, 3040, 4550, 9005

Mois, George: 4080, 4320, 8005

Mojtabai, Ramin: 7255

Mokhtari, Negar: 8000

Mola, Giada: 1330

Molina, Anthony: 4107, 7050, 7190, 7375

Molinari, Victor: 7390

Molinsky, Jennifer H: 7300

Moll-Jongerius, Annemarie: 4105

Molton, Ivan: 7495

Momin, Anmol: 7300

Monaghan-Geernaert, Pamela: 2290

Monane, Mark B: 7010

Mondol, Lucy: 1005

Money, Victoria L: 8005

Mongelli, Jessica: 2175, 8000

Monin, Joan K: 2095, 2135, 4490, 7210, 8000, 8005, 8010

Monroe, Todd B: 2045

Monroe-Lord, Lillie: 7050

Montalva, Nicolas: 1425

Montano, Anna-Rae: 7410

Montemuro Rode, Lauren: 7055

Montepare, Joann M: 1130, 2145, 3150

Montero Herrera, Bryan: 7435, 9130

Montero Odasso, Manuel: 7225, 7905

Montesinos, alexandra: 7315

Montgomery, Lynnette: 4525

Montgomery, Ruth R: 8000

Montgomery, Stephen: 1520

Monti, Stefano: 1520, 8015

Montoro-Rodriguez, Julian: 4085, 7230, 7305, 7470

Montoya, Ana: 7250, 8005

Montoya, Yadira: 3070

Moo, Lauren: 1305, 2085, 2365, 3005, 3115, 7230, 7280, 7295, 7310, 8005

Moon, Chooza: 1475, 4230, 7265

Moon, Heehyul E: 7160, 7350, 7415, 8000

Moon, Hyun: 7435

Moon, Jasmine: 7460

Moon, Jennifer: 2045

Moon, Saemi: 3020

Moon, Sanghee: 8010

Moone, Rajean P: 2010, 3000, 4220, 7370

Mooney, Aimee: 1015, 1165, 7415

Mooney, Graham: 2400

Mooney, Laken A: 7365, 7435

Moore, Alison A: 8015

Moore, Jeffry: 8015

Moore, Justin B: 4205

Moore, Kari A: 2400

Moore, Karlin: 4210

Moore, Laura: 1050

Moore, Michelle: 9055

Moore, Miranda: 2160, 3255

Moore, Raeanne C: 1295

Moored, Kyle D: 2400, 8000, 8005

Moorehead, Jeanmarie: 2440

Moorhead, Sue: 8010, 8015

Moorman, Sara M: 2295, 3050, 4520, 7355, 7900

Mor, Maria K: 9140

Mor, Vincent: 7540

Mora, Alexandra: 1145, 8015

Mora, Dalilah: 8015

Morag, Ido: 3200

Moraga Franco, Cristina: 4460

Morais, José: 7315

Morales, Gabriela: 7285, 7925

Morales, Santiago: 8005

Moran, Daniel S: 8005

Morgan, Amanda C: 7005

Morgan, Brianna: 2045, 2555, 4155

Morgan, Jasmine: 7025

Morgan, Jennifer Craft: 1005, 1275, 2225, 3040, 4550

Morgan, Robert O: 4475

Morgan, Sarah S: 3165

Morgan, Stephanie: 7420

Morhardt, Darby: 1015

Mori, Hiroki: 7265

Morin, Ruth: 7205

Morishita-Suzuki, Kumi: 7230

Morita, Kojiro: 9020

Moritz, Robert L: 7315

Moriyama, Yoko: 8010

Morone, Natalia: 4535

Moropoulos, Kaylie: 8005, 9120

Morris, Nichole: 8010

Morrison, Liane: 9105

Morrison, Sean: 4430, 7010

Morrison, Wanda: 7220

Morrow, Corey: 7495

Morrow, Hannah: 8005

Morrow-Howell, Nancy: 1195, 1425, 8000, 8005

Mortby, Moyra E.: 4470

Mortenson, W.Ben: 7205, 7915

Morton, Alec: 8005

Morton, Claire: 8005

Moschella, Sophia: 8015

Moseley, Sydney: 8015

Moses-Eisenstein, Michelle: 7020

Mosher, Hilary: 2085, 8005

Mosing, Miriam: 4030

Moslander, KoriAnne C: 7515

Mosley, Thomas: 1200, 7325, 7495

Mosqueda, Laura: 1215, 3300, 3350, 7230

Moss, Charlotte: 8005

Moss, Karen O: 2135, 8005, 8010

Mott, Catharine: 2475

Mould, Diane R: 8010

Moulton, Stephanie: 3285

Moura, Winny Eveny: 8000

Mowbray, Orion: 2340

Moxley, Jerad: 7540

Moy, Marilyn L: 7265

Moye, Jennifer: 2085, 7265

Moyer, Caleb R: 7200

Moyosoreoluwa, Jacobs: 2375

Mpofu, Elias: 7250, 7430, 8015

Mroczek, Daniel K: 1265, 3330, 4000, 4120

Mroz, Emily L: 1210, 1275, 2135, 3375, 8005, 8015, 9000

Mroz, Tracy M: 1270, 4450, 8000, 8010

Mu, Christina X: 8000

Muñoz, Elizabeth: 2005, 2345, 3060, 3395, 4110, 7145, 7200, 7435, 8000

Mubashshira, Tasneem: 2040, 8010

Muchow, Courtney R: 7340

Mudar, Raksha A: 1165, 4080, 8015, 9085

Mudhur, Jasmine: 7090

Mudrazija, Stipica: 2060

Mueller, Christine: 4340

Mueller, J. Tom: 7180

Mui, Ada Yuk-Sim: 9115

Mukherjee, Namrata: 7110

Mukherjee, Shalini: 4270

Mulhorn, Kristine A: 7520

Mullan, Aidan F: 9045

Mullen, Rebecca: 7290

Muller, Chandra: 2120, 7010, 7040

Muller, Emma: 4230

Mulligan, Grace: 8010

Mullins, C. Daniel: 7495, 8015

Mumma, Kara T: 1230

Munell, Alicia: 4100

Mungas, Dan: 4540

Muniz-Terrera, Graciela: 2510, 2545, 3265, 4110, 4120, 7145, 9005

Munjak-Khoury, Ceresa: 8000

Munly, Kelly: 4035

Munn, Lindsay Thompson: 7390

Munoz, Elizabeth: 1175

Munoz, Richard: 8010

Munro, Cynthia: 1510

Munshi, Nikhil: 8000

Munsterman, Ellen: 8010

Murabito, Joanne M.: 3127

Murabito, Joanne M: 8000, 8005

Murali, Komal Patel: 8010

Muramatsu, Naoko: 1160, 3355, 7015

Murayama, Hiroshi: 7345, 7375

Murdaugh, Kimberly: 3340

Murdock, Cristina: 8000

Murmann, Maya: 7370

Muroff, Jordana: 4075, 4345

Murphy, Blake: 1020

Murphy, Diane: 9065

Murphy, Erin C: 4035

Murphy, Rachel A: 4490

Murphy, Sara C: 2065, 7540

murphy, Terrence: 3220, 4480, 7500

Murray, Andrew: 1300

Murray, Briana: 1395

Murray, Camille: 8005

Murray, Catherine-Anne: 4500

Murray, Deven: 3245

Murray, Dorothea: 7135

Murray, Sarah: 1305

Murthy, Venkatesh: 2000, 2245, 7345

Musema, Jane: 3085

Musi, Nicolas: 2557, 4090

Musinguzi, Nicholas: 3020

Muskin, Ryan: 9055

Musty, Allison: 1255

Musunuri, Raaga Likhitha: 9045

Mutambudzi, Miriam: 8000

Mutz, Julian: 7090

Myagmarjav, Sugarmaa: 8015

Myatt, Glen D: 2535

Myers, Clarice: 2000

Myers, Keith: 2445

Myers, Todd: 8010

Myint, Wah Wah: 7375

N

Na, Annalisa: 8005

Na, Eunung: 8000

Na, Hunhui: 4420

Naas, Taqwa: 8010

Naavaal, Shillpa: 4440

Nabi, Michelle: 7410

Nabors, Emily: 4180

Nacimben, Gabriela: 7370

Nadarajah, Nandhini: 4425

Nadarajan, Gayathri Devi D/O: 2150, 3135

Nadash, Pamela: 3335, 7085, 7255, 8000

Nadig Nair, Krishnaa: 7540

Nadorff, Danielle K: 2340, 3335

Nagarajan, Niranjani: 8010

Nagel, Corey: 7165

Nagib, Ahsan: 4347

Nagy, Myra: 9105

Nagykaldi, Zsolt: 1510

Nah, Suyoung: 7230

Naher, Samsun: 2075

Naidu, Nidhi: 8010, 8015

Naik, Aanand D: 1035, 4445, 7030

Naim, Sigal: 1130, 4355

Nair, Anil: 7025, 7195

Nair, Kalyani B: 8010

Nair, Malini: 7025, 7195

Nair, Sridevi Unni: 7190

Najafi, Bijan: 7300

Najar, Mohsen: 3230

Nakagawa, Takeshi: 2120, 7365, 7380

Nakahara, Jun: 7355

Nakamura, Jeanne: 4300

Nakamura, Masahide: 7140

Nakanishi, Miharu: 7220

Nakashima, Taeko: 7330

Nakayama, Gen: 8010

Nakayama, Takeo: 2230

Nakhmani, Arie: 8000

Nalamachu, Sri: 7500

Naliyat, Lakshmi: 9130

Naliyatthaliyazchayil, Parvati: 9130

Nam, Gina E: 8000

Nam, Hee-kyoung: 7090

Nam, Sanggon: 8005, 8015

Nam, Youngwon: 7115

Namkung, Eun Ha: 1405, 7235

Nanna, Michael: 8000

Nanson, Kate: 7205

Napoleon, Betty J: 7410

Narine, Donnette: 7115

Naseem, Rahat: 3230

Nash, Paul: 1450, 2395

Nashef-Hamuda, Reem: 1125, 1240, 4205, 7410

Nathan, Rima: 4245

Navarro, Adria: 8010

Navarro-Millan, Iris: 4525

Navos, Melanie L: 8000

Nayak, Veena: 7510

Naylor, Mary: 8000

Nazario-Acevedo, Jaminette M: 7145, 7900

Ndebele, Hanani: 7010

Ndjaboue, Ruth: 4150

Nduka, Chinelo: 7245, 8005

Ndukwe, Derrick: 9130

Ndurue, Light O: 3035, 7465, 9030

Neal, Amy: 8005

Nearing, Kathryn A: 1130, 2195, 7170, 7410, 7910

Needham, Valerie: 8005

Negassa, Meti: 8015

Nehrkorn-Bailey, Abigail: 7265

Neiberg, Rebecca H: 2470

Neilands, Torsten: 7260

Neises, Kristen: 3240

Neller, Sarah A: 2135

Nelligan, Labrini: 9125

Nelson, Elizabeth: 1190

Nelson, Heather H: 7425

Nelson, Ian Matthew M: 2335, 7330, 7420

Nelson, Lindsey C: 8010

Nelson, Matt: 2335, 4160, 7250

Nemati, Donya: 2135, 4560, 9130

Nemec, Mary: 8005

Nemeth, Samuel: 1195, 2320

Nemia, Gianna: 2275

Nemmers, Natasha L: 1060, 1120, 3010, 4485, 7230, 8005

Neogi, Tuhina: 1070, 7120

Neri, Melinda: 7215, 7925, 8000

Nesbit, Tyler S: 7350

Netta Bentur, Netta Bentur: 7090

Nettles, Brenda: 7030

Neumann, Lycia: 4160

Neupert, Shevaun D: 1120, 1480, 3095, 3360, 4270

Neville, Charlotte: 4205

Nevriana, Alicia: 1425

Newell, Bradley J: 3255

Newman, Anne B: 2000, 2245, 2470, 4295, 7345, 7425, 8000, 8010

Newman, David: 2045

Newsham, Tina: 1380, 1390, 2330, 3120, 3295, 7150

Newton, Wendy: 7045

Ng, Angus Jun Jie: 2150

Ng, Boon Peng: 2435, 8000

Ng, Boon Peng: 8015

Ng, Boon Peng: 8015

Ng, Christine Tsz Ying: 7085

Ng, Clement Cheuk-Wai: 7395, 7440

Ng, Crystal: 4490

Ng, Crystal: 9085

Ng, Wee Qin: 3275

Ng, Xuan Min: 4425

Ng, Yee To (Crystal): 4195, 4360

Ngai, Joe Tsz Kin: 1385

Ngan, Kerry: 1340

Ngandu, Tiia: 1290

Ngo, Duyen: 7280

Ngo, Long H: 2505, 8000

Ngo, Victoria: 1305, 2365, 7230, 7280

Nguyen, Andrew H: 2365, 8005

Nguyen, Anh Thy: 7185

Nguyen, Ann W: 1415, 3245

Nguyen, Annie: 8015

Nguyen, Brian: 8015

Nguyen, Christopher P: 2135

Nguyen, Dinh: 1355

Nguyen, Duy: 7300

Nguyen, Hoang Van: 4347

Nguyen, Louis L: 8005, 9150

Nguyen, Minh: 2275

Nguyen, Nguyen TK: 7440

Nguyen, Tam: 3155

Nguyen, Taylor: 7435

Nguyen, Thai: 8010

Nguyen, Tra: 4535

Nguyen, Xuan-Mai T: 8010

Ngwa, Che Henry: 1495

Ngwu, Millicent O: 8015

Ni, Xuelei S: 8015

Niceforo, Michaella: 8015

Nicholls, Karen: 4500

Nichols, Emma: 1040, 2000, 3380, 4280, 4540

Nichols, Madeline: 7335

Nicholson, Jody S: 1285, 8000

Nickels, Shirley: 7225

Nickerson, Allison: 7540

Nickerson, Brett: 2220

Nicklas, Barbara J: 2470, 4205, 7165

Nicklas, Barbara: 8005

Nicklett, Emily J: 3245

Nicolay, Sarah: 2485, 9010

Nicoloro-Santabarbara, Jennifer: 7460, 8000

Nie, Hao: 7320

Nieboer, Anna P: 7260

Nielsen, Natalia: 7255

Nigro, Lisa: 8005

Nikitin, Jana: 1210, 1350, 3145, 4270

Nikpay, Sayeh: 3095

Nikzad-Terhune, Katherina A: 2330, 3150

Nilsen, Charlotta: 7375

Nimrod, Galit: 1375

Nishar, Shivani: 4010

Nishida, Yoshifumi: 7225

Nishimura, Jun: 7370

Nishino, Kenshi: 8010

Nishita, Yukiko: 7330

Nissen, Adam T: 1265

Niu, Jinbo: 8010, 8015

Nix Parker, Tamara: 4445

Nixon-Lambert, Erika: 7340

Niznik, Joshua: 1235

Nizza, Megan: 4075, 4345, 4530

Nketsiah, Ebow T: 8000

Nkimbeng, Manka: 2010, 3155, 3375

Nnadi, Laura: 2395

Noble, Liz: 4180

Noble, Molly E: 1300, 2260

Nocera, Joseph (Joe): 7225

Noel, Lailea: 4530

Noel, Sabrina E.: 8000, 8015

Nogal, Bartek: 7015

Noguchi-Watanabe, Maiko: 8000

Noh, Heeju: 7090, 7315

Noh, Hyunjin: 1365, 7310, 7500

Noh, Sung Gi: 8000

Nolan, Cindy: 7410

Nolan, Raena N: 7295

Noll, Laura: 7365

Nolte, Julia: 3275

Nomura, Ayano: 7225

Nomura, Kazuhiro: 8015

Noonan, Curtis: 9015

Noonan, Madeline: 2310

Noor, Mirza Ishrat: 8015

Noppert, Grace A: 4440, 8015

Nordyke, Alexandra: 7010

Noriega, Xavier: 2525, 7110

Noriega-Makarskyy, Daisy T: 1100

Norihiro, Nishioka: 2230

Norris, Emily: 7185

Norstrand, Lu: 7340, 7935

Northcutt, Caitlin A: 3370, 4030, 4560

Norton, Jonathan D: 7385

Nothelle, Stephanie: 8005

Nothern, Alexandra: 9080, 9090

Noun, Margaret: 1160

Noureldin, Marwa: 2455

Nouryan, Christian: 7215, 7410, 8000, 9055

Novack, Miriam A: 7435

Noval, Christian: 8005

Novisky, Meghan: 3235

Novruz, Ayla: 2400

Nowakowski, Alexandra “Xan” CH: 4220

Nowels, Molly: 4135, 7500

Nowinski, Cindy J: 7435

Nsor, Neke: 2025

Ntinda, Kayi Ntinda: 8015

Nuckols, Teryl: 8000

Numata, Hanako: 2210, 8000

Nunez, Johanna G: 3265

Nunez, Matthew: 4415

Nurse, Ashley: 7210

Nusetor, Frederick: 4460

Nutakor, Jonathan A: 7425

Nute, Andrew: 9015

Nwakasi, Candidus: 7200, 7245, 8005

Nweke, Chizobam: 7185, 8010

Nyblade, Laura: 7530

Nygaard, Marianne: 4030

Nyhuis, Casandra: 1510

O

Oakes, Connor: 7190

Oakman, Kimberly: 8010

Obando, Viviana: 7025, 7195

Obeid, Carla: 7145

Obeng, Afia: 8005

Obermiller, Corey: 7390

Obi, Perpetua C: 7300

Obioha, Wisdom C: 8015

Oblon, Nicole: 7090

O’Brien, Amanda: 7265

O’Brien, Catherine: 7440

O’Brien, Erin: 2350

O’Brien, Jennifer L: 1285

Ocoko, Faith: 7270

O’Connell, Denise: 9125

O’Connell, Megan E.: 2545, 8015

O’Connor, Maureen K: 2365, 3005, 7195, 8005

O’Connor, Melissa: 4560

O’Connor, Victoria: 7410

Odabas, Sena: 7365

Odden, Michelle: 8000

O’Dea, Mary: 8000

Odebiyi, Bolanle: 9085

Oderberg, Jane: 2065

Odo, Obinna C: 1365, 7200, 7330

Odom, Hannah: 7265

Odom, J. Nicholas: 4445, 8015

O’Donnell, Amy J: 7400

O’Donnell, Arden: 4575

O’Donnell, Mary Gemma: 2170

O’Donnell, Mary Gemma: 8005

Oduro, Elisha B: 1145, 8000, 8015

Ofili, Divine-Favour: 1080

Ofir, Ruty: 1515

Ofori, Edward: 7315

Ofrey, Ebi: 1430

O’Gara-Standiford, Shannon: 1215

Ogawa, Elisa F: 7040, 8010, 9095

Ogawa, Madoka: 7395, 7925

Ogbuniro, Joseph: 7300

Ogden, Lydia P: 1410

Oginni, John: 9025

Ogland-Hand, Suzann: 2240

Ogle, Destiny M: 2405, 4040, 4185, 4315, 4485, 7085, 7470

Ogletree, Aaron M: 1505, 4007, 7105

O’Grady, Thomas: 7330

Ogugu, Everlyne G: 2450

Ogunjesa, Babatope: 3205

Oh, Esther S: 8005

Oh, Eunna: 7150, 8005

Oh, Hamilton Se-Hwee: 1155

Oh, Haneul: 7395

Oh, Hans: 4125, 4250

Oh, Jeewon: 2405

Oh, Kyeung Mi: 7545, 7910, 8015

Oh, Oonjee: 7205, 7220, 8015

Oh, Patricia: 8010, 8015, 9115

Oh, Sungho: 9135

Oh, Yun Taek: 1115, 3175, 7070

Ohana, Irit: 8005

O’Hara, Leeanne: 4205

Ohno, Yuka: 8005

Ohwobete, Jemima: 4305

Ojaimi, Nadim: 2125

Ojeda, Lauro: 8010

Ojumah, Esther O: 8000

Okada, Akira: 7250

Okada, Hiroshi: 2230

Okada, Jamil: 8005

Okamura, Tsuyoshi: 8010

Okechi, Ikenna D: 8015

Okereke, Olivia I: 1160

Okine, Joana: 2340

Okolie, Tochukwu J: 1365, 7085, 7915, 8005

Okoli-Umeweni, Adaora: 4570

Okoye, Safiyyah M: 4210, 7360

Okun, Sarit: 2370

Oladimeji, Abolade: 8005

Olander, John: 9105

Olanrewaju, Sherif A: 8005

Olaru, Gabriel: 1240, 3270

Olayeni, Bolanle: 1505

Olds, Timothy: 3235

O’Leary, Bryant J: 7265

O’Leary, Justin M: 8005

O’Lenick, Cassandra R: 3235

Olinger, Bradley: 7480

Oliveira Bueno de Souza, Roberta: 7140

Oliven, Ron: 7060

Oliver, Brant: 1485

Oliver, Debra: 7220, 7260

Oliver, Emily: 1095

Olivero, Noah: 9065

Olivier, Michael: 3397

Olivieri-Mui, Brianne: 9080

Olsan, Tobie: 7495

Olsen, Bonnie J: 1215, 7230

Oltmanns, Thomas F: 2350, 4040, 7525, 8005

O’Mahony, Sean: 8005

O’Malley, Kelly A: 2110, 7455, 7935, 8010

O’Mara, Megan: 7145

Ommering, Belinda W.C.: 4105

O’Neil, Maya E: 8010

O’Neill, Desmond: 4115

Oner, Sudenaz Fatma: 7315, 8005

Ong, Anthony D: 1265, 4120

Ong, Chong Yau: 2150

Ong, Liane: 7185

Ong, Lydia Q: 1480

Ong, Marcus EH: 2150, 3135

Ono, Mayo: 7235

Onorato, Nicole: 7545

Onsando, Wambui Moraa: 2045, 4150

Onuh, Samuel C: 1365

Onyedibe, Maria Chidi C.: 2220

Onyema, Ezenwa: 8000

Opazo Breton, Magdalena: 1245

Opitz, Alexander: 7025

Oppong, Beatrice: 4325

Oppong, Kwaku Duah: 4445, 8015

Ordoñez, Maria J: 7285, 7925

O’Reilly, Maria: 7155

O’Reilly-Jacob, Monica: 7185

Orejuela, Melanie: 7350

Orewa, Gregory N: 1150, 1430, 3365

Orkaby, Ariela: 8000, 8010, 9155

Orkibi, Hod: 1330, 4205, 4235

Ornelas Van Horne, Yoshira: 2005

Ornstein, Katherine: 1025, 1270, 2190, 2375, 2480, 4070, 4385, 4450, 7390, 7545, 8005, 8010

O’Rourke, Hannah: 3040

Orozco, Tatiana: 7350

Orr, Miranda: 2557

Orsega-Smith, Elizabeth: 7130

Orsulic-Jeras, Silvia: 7185, 8000, 8015, 9010

Ortega, Calyope: 7470

Ortega, Felix: 7345

Ortega, Julian: 8015

Ortega-Ruiz, Frida: 8000

Ortiz, Gabi Celia: 7110

Ortiz Villaman, Jezebel: 8010

Ortiz-Ortiz, Karen: 2555

Orwig, Denise: 2395, 4070, 7545, 8015

Orwoll, Eric: 1460

Ory, Marcia: 7475, 9025

Osal, Ryan: 7225

Osareh, Helia F: 7495

Oseroff, Benjamin H: 2480

Osevala, Nicole M: 4480

Osime, Esther: 7085

Osokpo, Onome H: 4070, 7430

Ospina, Natalia: 4020

Ost-Mor, Sharon: 1090, 1365, 3040, 7450

Østbye, Truls: 7520

Ostrem, Nicholas: 8000

O’Sullivan, Kelly S: 7175, 7920

O’Sullivan, Roger: 1185

Oswald, Austin: 2525, 7110, 7535

Ota, Tomoyuki: 7465

Otgon, Saranchuluun: 8015

Otsuka, Rei: 7330

Otto, Amy: 4045

Otu, Hasan H: 2505

Ou, Alison Y. T.: 1225

Ouellet, Gregory M: 7410, 7920

Ouellet, Jennifer A: 7410, 7920, 8005

Ouellet, Marie-Christine: 8005

Ouimet, Ethan: 7265, 8000

Oullet, Jennifer: 7030

OUYANG, FUXI: 3215, 8015

Ouyang, Meishuo: 1235, 4010

Ouyang, Rui: 8005

Overbeck, Kevin: 4570, 7340, 7410

Overstreet, Abigail: 8015

Owe, Brenda: 7310

Owen, Donna C: 7125

Owens, Otis L: 7545

Owens, Victoria: 1370

Owings, Michael J: 7425

Owoaje, Eme T: 4565

Owusu Ansah, Osborn: 7415

Oyebode, Jan R: 2335

Oyedeji, Charity I: 7170, 7220

Ozawa, Tabasa: 4435

Ozor, Tochukwu: 7330

P

Pace, Thomas: 4580

Pacheco, Kelly: 1055

Pacheco, Laura: 7065

Pacheco Busquets, Mariana: 7495

Pack, Lindsay: 1300

Packer-Porat, Tammi: 3040

Padeiro, Miguel: 7370

Padilla, Paola: 3030

Padilla-Frausto, D. Imelda: 8000

Pagan, Michelangelo J: 4200

Pagotto, ´Valeria: 8000

Painter, Mairead: 4405

Pajewski, Nicholas M: 1110, 1255

Pakpahan, Martina: 8000

Paladino, Joanna: 7185, 7205

Palakshappa, Deepak: 7390

Palaniappan, Sucheendra K: 8005

Palavicini, Juan P: 8015

Paleski, Sophia L: 7365, 7400

palgi, Yuval: 1125, 2105, 3200, 3360, 4270, 9095

Palladino, Dianne: 4020

Pallas-Brink, Jara: 9145

Palleschi, Mary Kate: 8010

Pallis, Maryssa: 4565

Palm, Matthew: 4245

Palmer, Allyson K: 8005, 9045

Palmer, Jennifer: 4210

Palta, Priya: 1200, 2535, 3185, 4375, 7495, 8005, 8010

Pan, Honghui: 1290

pan, Jinsong: 8010

Pan, Kuan-Yu: 1425

Pan, Min-Hsiung: 8015

Pan, Qianhui: 7015, 7095

Pan, Wei: 3135

Pan, Xi: 7040

Pandare, Siddhi V: 2350

Pandey, Ashwini: 9080

Pang, Marco YC: 9020

Pang, Yijiang: 3090

Pang, Yuk C: 8010

Pankow, James S: 1200, 8015

Pantzer, Christina: 7210

Panzer, Victoria: 9020

Paoletti-Hatcher, Jensine: 8000

Paolino, Valerie: 7385

Papadopoulos, Airia: 3155

Papaefthymiou, Konstantinos: 7465

Pappa, Sara T: 4190

Pappadis, Monique R: 7230, 7405, 9025

Paramesh, Colleen: 8000

Parchment, Amelia: 9085

Paredes, Aasha: 8005

Paredes, Lucero G: 1375

Parent-Lamarche, Annick: 7325

Parihar, Madhur: 1355

Parikh, Romil R: 1010, 1430, 2160, 2260, 3020, 3075, 3190, 7300, 7530

Parisi, Jeanine M: 8005

Park, Chan Mi: 9000, 9080, 9085, 9105, 9135, 9145

Park, Chang Gi: 7325, 7330, 7525

Park, Chan-Young: 8000

Park, Chorong: 7015

Park, Chorong: 8000

Park, Daniel: 7085

Park, Do-Hyung: 4180

Park, Eun Ju: 7145, 7300, 7375

Park, Eunyoung: 7220, 7425, 7430

Park, Geunhye: 7255

Park, Hyejin: 7265

Park, Hyeyoung K: 3040, 7220, 7910

Park, Hyung In: 7380

Park, Isac: 4200

Park, Jae: 3030

Park, Jeongmin: 7365

Park, Jihoon: 1385

Park, Ji-Hyuk: 7335

Park, Jina: 1515, 8005

Park, Jiwon: 3025, 3075, 4420

Park, Joonhyeog: 7295, 8010, 8015, 9030

Park, Joon-Hyuk: 7545

Park, Joon-Hyuk: 8010

Park, Jung In: 7210

Park, Junmin: 4210

Park, Juyoung: 1020, 4045

Park, Juyoung: 4465

Park, Juyoung: 7010

Park, Juyoung: 7210, 7355

Park, Juyoung: 7500

Park, Kyoung Shin: 7435, 8005, 8015, 9025, 9130

Park, Kyoungjae: 3020

Park, Min Kyung: 7225, 7300, 7325, 7375, 7395, 7520

Park, Moonkyoung: 7325, 7430

Park, Nan Sook: 3325, 7210, 7305

Park, Pil: 8005

Park, SaeMi: 4560

Park, Seeun: 7495

Park, Seokyung: 7360

Park, Seyeon: 7430

Park, Shin Young: 7370

Park, Sikyeong: 7520

Park, Sojung: 7275, 7290, 7470, 7495, 7925, 8005, 8010, 8015

Park, Soobin: 7400, 7495

Park, Soobin: 8005, 8010

Park, Soohyun: 8005

Park, Soojin: 3285

Park, Sooyoung: 7400, 8015

Park, Sophie: 8000

Park, Subin: 7375

Park, Sunbok: 8010

Park, Sung S: 7395

Park, Sunyoung: 7540

Park, Susan: 7540

Park, Thomas: 7315

Parker, Andrew M: 1330

Parker, Daniel C: 7010

Parker, Kelly: 1175, 7325

Parker, Kimberly: 8010

Parker, Lauren J: 8015

Parker, Tassy: 2170

Parkman, Suzanne: 4105

Parks, Anna: 7185

Parks, Christine G: 1500

Parmanto, Bambang: 2375

Parmar, Naaz: 7225, 7905

Parnia, Sam: 7330

Paro, Ashley: 8005

Parrish, Elise: 2375

Parry, Jayne: 7310

Parson, Britney: 2110

Parsons, Helen M: 3095

Parsons, Michael: 8015

Parvatini, Supriya: 7410

Pasarariu, Alexandru L: 2045

Pasatiempo, Ana Maria G: 8005

Pascual-Leone, Alvaro: 7025, 7435

Passey, Richard: 7010, 7910

Passmore, Susan: 7270

Pate, Melannie: 7005

Patel, Arianna: 8005

Patel, Asiya: 8010

Patel, Hanna: 8015

Patel, Himalaya: 7415

Patel, Jaahnvi: 7225

Patel, Jahanvi: 7050

Patel, Kamal: 7410

Patel, Kushang V: 4535

Patel, Nidhi: 1505

Patel, Prisha: 4415

Patel, Priti: 2075

Patel, Ratna: 4470

Patel, Reeya: 4250

Patel, Snehal: 7305

Patel, Suniska: 7340

Patel, Tejal: 7230

Paterson, Thomas: 7415

Pathak, Anangsha: 4580

Patil, Amruta: 4210

Patil, Shivrajo: 7010

Patrick, Emily: 7470

Patrick, Megan E: 1105, 8010

Patriquin, Michelle: 8005

Patskanick, Taylor: 1405

Patten, Amy B: 8010

Patterson, Charity: 8000

Patterson, Joanne G: 1000

Patterson, Kelsey: 8015

Patterson, Logan: 3295

Patterson, Mary: 8015

Patterson, Meagan M: 3150

Patterson, Sarah E: 2375, 3050, 4360, 4485

Patyal, Pankaj: 7010, 7190

Pau, Massimiliano: 7265

Paudel, Anju: 3220, 4460

Paul, Constanca: 7135

Paul, Shreya: 8000

Paul, Todd: 1050

Paulsen, Michelle R: 7410

Paulson, Daniel: 7375

Paulson, Sally: 7120

Pauly, Theresa: 1480, 3305, 4515

Paun, Olimpia: 2300, 8000

Pavlik, Jan: 7295

Pavon, Juliessa M: 3065

Payne, Jodi: 7455

Payne, Laura: 8005

Paz, Miguel: 8010, 8015

Peacock, Brandi: 7270

Pearce, Mason: 8005

Pearl Naim, Sigal: 7175

Pearlman, Jon: 4305, 7515

Pearlstein, Jennifer G: 2155

Pearman, Ann: 1120

Pearson, Megan: 1470

Pecic, Stevan: 7415

Peckham, Allie: 1440, 2030

Pedersen, Jacob K: 7345

Pederson, Maja: 9015

Pederson, Tabitha: 3245

Pedro, Sofia: 7120

Peduzzi, Nora: 7250

Peek, Gina: 4210

Peeler, David: 1230

Peeples, Amanda D: 8015

Peeples, Landon B: 7305, 7400, 7415, 7900

Peereboom, Danielle: 2520

Peerman, Carey: 1135

Pei, Heming: 8000, 8010, 9150

Pei, Xiangyu (Annie): 8015

Pei, Yalian: 8005

Pei, Yaolin: 1400, 2475, 3015, 3290, 4065, 4170, 8005

Pei, Zhongfei: 8005

Peiffer, Elizabeth: 7225

Pekaar, Keri A: 1480

Peleg, Shira: 2390

Pellegrini, Christine: 7435

Pelletier, Stephen: 8005

Peltz, Carrie: 4335

Peltzer, Jill: 8010

Pendergast, Jacquelyn: 4090

Pendrey, Anna: 8010, 8015

Peng, Changmin: 3335, 7355

Peng, Huamao: 7340

Peng, Rong: 7540

Peng, Siyun: 2360

Peng, Wenting: 7100, 8015

Peng, Xinyuan: 7375

Penhale, Bridget L: 3350

Peniche, Jorge: 9075

Pennavaria, Adam: 8005, 8015

Pennella, Jenna: 4095

Pepin, Marc J: 3065

Pepin, Renee: 2395, 4020, 7300

Perales, Jenna: 8000

Perales-Puchalt, Jaime: 4230

Peralta, Laura S: 8005

Perepezko, Kate M: 2445, 3135, 4160

Perera, Dinithi: 1290

Perera, Dinithi: 8010

Perera, Subashan: 8005

Perez, Fiorella: 7455

Perez, Richard: 7370

Perez Montes, Viviana: 4008

Perezalonso Espinosa, Jeronimo: 1155

Perissinotto, Carla A: 3050, 4020, 4070

Perkhounkova, Yelena: 1150, 1515, 2225, 3220, 4015, 7250

Perkins, Molly M: 1275, 2160, 4135, 8010

Perkins, Rebekah: 4555, 7405

Perls, Thomas: 1520, 4440, 7310, 8015, 9155

Peron, Emily P: 1260

Perone, Angela K: 1380, 1450, 2070, 4165, 7235, 8005

Perre, Taylor: 7220

Perrin, Nancy: 1345

Perron, Rebecca: 2530

Perry, Andrea: 7305

Perry, Audrey J: 2455

Perry, Brea L: 1095, 2360

Perry, Dafna: 1520

Perry, Morgan: 7485

Perry, Tam E: 2215, 2515, 3305

Perryman, Erica M: 7005

Persad, Alyssa: 7320

Persky, Victoria: 2040

Persson, Annelie M: 7430

Perweiler, Elyse: 7410

Perzynski, Adam: 8005

Pesavento, Matthew: 3285

Pescador Jimenez, Marcia I: 2400, 9040

Peskoe, Sarah: 8000, 8015

Peterfreund, Ilana: 1485

Peterman, Leah M: 8010

Peters, Case: 4205, 7390

Peters-Beumer, Lisa: 4355, 7400, 8005

Peterson, Colleen M: 7155

Peterson, Elizabeth W: 7225

Peterson, Lindsay J: 7175, 7220, 7290, 7340, 7370, 7420, 7935, 8000

Peterson, Rachel: 9015

Peterson, Tina: 3055

Petralia, Kelley: 8005, 8010

Petroni, Alessia G: 7365, 7400

Petrovsky, Darina: 7415

Petrowitz, Bradley: 7310

Pettee Gabriel, Kelley: 1110, 7325

Pettis, Jennifer: 1395, 9010, 9125

Pfeil, Carmen: 8005

Pflieger, Lance: 7090, 7190, 7315

Pfund, Gabrielle N: 1045

Pfund, Gabrielle: 8000

Pham, Julie Q: 8010

Pham, Nghia: 8010

Pham, Tony V: 2310

Phan, Matthew: 8000, 8015

Phaowiriya, Hathaichanok: 7245

Phelan, Elizabeth A: 4015, 8010, 8015

Phelps, Brenna: 7225

Phibbs, Ciaran: 2225, 4550

Philip, Celene: 7290

Philips, Kira C: 2320

Phillips, Carolyn: 8010

Phillips, Christy: 1285

Phillips, Jakob: 7375

Phillips, Joseph R: 7095

Phillips, Meredith: 2200

Phillips, Olive: 7410

Phinney, Alison: 2420

Phipps, Stephen M: 7190

Phongtankuel, Veerawat: 1170, 8000

Phuah, Chia-Ling: 1355

Pi Ching, Weng: 7410, 7935

Pianosi, Birgit: 7075

Piaseu, Noppawan: 7130, 7430

Piazolo, Felix: 3215

Piazza, Jennifer R: 2095, 2280, 4120, 7310, 7455, 8015

Picella, David V: 8005, 8010, 8015

Pickell, Delaney: 8010

Pickering, Carolyn E: 1085, 4085, 7015, 7385, 8005

Pickett, Andrew C: 3375, 7305

Piechota, Amanda: 4475, 8005

Piedad, Mary: 7090

Piedra, Lissette M: 4085, 8010, 9005

Pierce, Audrey: 4227, 7315

Pierce, Matthew J: 1175, 7380

Piippo, Elizabeth: 7400

Pike, James Russell: 1200, 2535, 3185, 4065, 4450

Pike, James: 8005

Pillemer, Karl: 1215, 2325, 2385, 3145, 7350, 7455, 7915

Pimentel, Camilla B: 3115

Pincus, Harold: 7020

Pinheiro-Chagas, Pedro: 8015

Pinto, Jayant: 1500

Pinyan, Elizabeth C: 8005

Piol, Silvia: 1330, 4235

Pirela, Rosa V: 8015

Pisano, Michele: 9055

Pitcher, Katherine: 8000, 9135

Pittard, Colleen: 4020

Pitts, Jessica: 7225

Pituch, Keenan A: 1110, 2030

Pitzer, Kyle: 7260

Pius, Ruth: 8000

Piyasinchai, Bell: 7035

Plank, Emma: 8000

Plankey, Michael: 2140

Plankey, Michael: 2395

Plasencia, Rosa: 3190

Plassman, Brenda L: 1500, 2475

Platts, Loretta G: 3310

Pless Kaiser, Anica: 2085

Pliskin, Neil H: 3355

Plourde, Kendra: 8010

Plow, Matthew: 7430

Plumb, Jennifer: 4525

Plys, Evan: 2310, 4135, 4590, 7500, 9105

Poblete, Ronald: 9035

Pock, Eva Maria Lissa: 1310

Poe, Abigail: 4140

Poganik, Jesse R: 9150

Poghosyan, Lusine: 7125, 7185

Pogorzelska-Maziarz, Monika: 3040

Pohnert, Anne M: 1485, 7410

Polansky, Helena: 7395

Polenick, Courtney A: 8005

Polk, Rebecca: 7460, 8000

Pollack, Craig: 4210, 7420

Pollack, Kathryn I: 3375

Pollak, Chava: 8015

Polsky, Daniel: 1360, 2520

Polychronopoulou, Efstathia: 7535

Pomeroy, Mary Lou: 2480

Pomeroy, Mary Louise: 2190, 2375, 2480

Pomier Maren, Yisel: 7075

Pompeu, Jose Eduardo: 7140

Poncé, Díana: 1420, 8000

Ponce-Ponte, Oscar J.: 8010

Pond, Brittney L: 1420, 7215, 7925

Pontiff, Mattie: 7140

Ponto, Kevin: 2445

Poole, Patricia: 2475

Poole, Tamara S: 7170, 7220

Poon, Leonard W: 1065

Poot, Charlotte C: 1010

Pope, Natalie E: 2170, 3000, 4130

Pope, Natalie S: 7475

Pope, Taylor A: 2295, 8000

Pope, Zachary C: 1510

Popejoy, Lori: 8010, 9140

Porat-Dahlerbruch, Josh: 7125

Porcu, Eleonora: 8010

Portacolone, Elena: 8005, 9000

Porter, Gillian: 1175

Porter, Hunter L: 8005

Porter, Kristen E: 4220

Porter, Laura: 2110

Porter, Laura S: 7205

Portz, Jennifer D: 4090, 9090

Possin, Katherine: 2245, 9000

Pot, Anna: 4040, 8000

Potter, Claire: 4205

Potter, Sarah Nelson: 8010

Poulin, Valérie: 2235, 8005

Pound, Abby: 8010

Powell, Danielle S: 2520, 8005

Powell, Darcey N: 3150

Powell, Lenny: 4570, 7340

Powell, Victoria D: 4450

Powell, Wizdom: 3375, 4485

Power, Shannon R: 9065

Powers, Becky B: 2195

Powers, James: 3140, 4115

Powers, Sara: 2285, 7010, 7185, 7915, 8000, 9010

Powers, Timothy: 4175

Pradeep Kumar, Danya: 7090

Pradhan, Rohit: 1150, 3365

Pramana, Gede: 7495

Prasad, Anyah: 1095, 2390, 2525

Praska, Nicole A: 8005

Prefontaine, Nicholas M: 7400

Press, Valerie G: 7430

Presser, Kelly: 1300

Presti, Peter: 1230

Pretzer-Aboff, Ingrid: 4200

Preuss, Benedikt: 1310

Previtali, Federica: 3145

Prevratil, Michael J: 4420

Price, Ashley: 9150

Price, Elvin: 1260, 7340

Price, Nathan: 7015, 7190

Prieto, Lucas R: 7065, 7900

Prieto, Sarah: 7290, 7305, 8010

Prina, Matthew D: 2335, 3145

Principi, Andrea: 7070

Pritchard, Kevin T: 1340, 4090, 4525, 7495

Pritchard, Kevin T: 9135

Prizment, Anna: 7345, 7425, 8015

Procter-Gray, Elizabeth: 8000

Promislow, Daniel: 3267, 7045, 7315

Prophater, Lorna: 9005

Proulx, Christine: 4485

Province, Michael A: 8005

Provis, Javiera: 9125

Prusin, Todd: 7440

Prussman, Kyla: 7515

Prusynski, Rachel A: 1270, 8000, 8010

Psyllos, Marco: 3125

Puca, Amy: 1160, 1225

Puccio, Ava M.: 7000

Pudder, Anna: 7455

Puga, Frank: 2030, 3370, 4140, 7245, 8000, 8010, 8015

Pugh, Kendra: 2365

Pullen, Carly E: 7005

Pulpitel, Tamara: 4347

Puma, Litzy: 8010

Punnam, Saahithi: 8005

Punsalan, Ali: 8015

Punshon, Sarah: 3005, 4350

Puri, Jhanvi: 8015

Purol, Mariah: 3270

Purvis, Martha: 1260

Puspitasari, Vivien: 8000

Putnam, Michelle: 1410, 2430, 3117, 3320

Puts, Martine: 7090

Puttre, Hannah: 9150

Putzu, Valeria: 7265

Puxty, John: 1280

Pynoos, Jon: 7300

Q

Qan’ir, Yousef: 8000

Qazi, Saadia: 8000

Qi, Xiang: 1400, 2475, 3015, 3110, 4055, 4170, 4505, 7485, 8000, 8005, 8010

Qian, James: 4030

Qian, Jing: 7370

Qian, Yiqing: 1465, 2190, 2375

Qian, Yuting: 3200, 7250, 8015, 9010, 9140

Qiao, Yujia: 8000

Qin, Chengfan: 4055

Qin, Lu: 1375, 1510, 3010

Qin, Weidi: 1415, 3245, 4520

Qin, Ying: 2305

Qin, Yue: 2120

Qiu, Chen: 2545, 4065

Qiu, Peihua: 8010

Qiu, Shirley S: 7535

Qiu, Weiyu: 7410

Qiu, Xiao: 8015

Qiu, Xichenhui: 4505

Qiu, Yiming: 4385

Qu, Shan: 7170, 7210, 7305, 7375

Qu, Zhijing: 2260

Quach, Emma: 1305, 7295

Quach, Ha-Linh: 1205

Quach, Lien: 7245

Quan, Jianchao: 1030

Quan, Stuart F: 7390

Quashie, Nekehia T: 3195, 8015

Query, Sharon: 2290

Quesnel-Vallée, Amélie: 1080

Quezada, Alejandro: 7020

Quiñones, Ana: 4470

Quiñones-Cordero, Maria M: 3375, 7230, 7305

Quiben, Myla: 7140

Quigley, Denise: 3010

Quigley, Molly: 7435

Quiles, Noberto: 8010

Quiles, Rosalphie: 4090

Quillen, Crystal A: 3150

Quinn, Joseph: 2255

Quinn, Joseph F: 3310

Quinn, Martha: 7390

Quinn, Sandra: 8000

Quinto, Marcelo: 3165

Quiroz, Yakeel T: 8000

Qureshi, Nabeel S: 8010, 9020

R

Rabalais, Jennifer: 1335

Rabello, Gilciney A: 7370

Rabey, Robin: 7455

Rabin, Laura A: 4310

Rabinowitz, Jill: 4375

Rabinowitz, Seth: 8015

Racoosin Kornmeier, Jillian: 2495, 4020

Radcliffe, Payton: 7350

Rademacher, Emma: 3305

Radpour, Siavash: 3310, 3385

Radspinner, Rachel: 7415

Raftery, Daniel: 7045

Ragot, Samuel: 7065

Ragsdale, Luna: 1085

Ragsdale, Vickie S: 7125

Rahat, Nazm: 7025

Rahemi, Zahra: 7205, 7220

Rahman, Momotazur: 1270, 2465, 3280, 7540

Raimondi, Alessandra: 3070

Raina, Parminder: 2545

Rainier, Riva: 7050

Rainville, G.: 7025

Raisa, Tasnim: 7015, 7385

Raj, Minakshi: 1165, 4565

Rajagopal, Krishna: 8000

Rajashekara Swamy, Meghana: 7530

Rajendran, Priyadharsini: 7510

Rajesh, Bhavana: 8005

Rajeshkumar, Nethra: 8005

Raji, Mukaila: 1070, 7305, 7535, 8000, 8005, 8015

Rakes, Sarah: 8000

Ram, NIlam: 1350, 4515, 8000

Ramadoss, Kamala: 7340

Ramamurthi, Hema: 7545

Raman, Rema: 3375

Ramaswamy, Gomathi: 7510

Ramirez, Paulino: 8010

Ramirez Gomez, Liliana: 3025, 7185, 8000, 8015

Ramirez-Garcia, Daniel: 1155

Ramirez-Perez, Nadeshka J: 8000

Rammal, Hassan: 1520

Ramos, Katherine: 7205

Ramos Espinoza, Hillary M: 9080

Ramprasad, Nidhi: 4135

Ramsburg, Hanna: 8000

Ramsey, Jennifer: 7230, 7305

Ramsey-Tobienne, Alexis E: 4355

Ramulu, Pradeep: 7530

Rana, Sangeeta: 7410

Rand, Debbie: 7090

Randall, George Kevin: 7400

Randall, Lindsey: 8015

Randolph, Madisin: 7270

Rangan, Apoorva: 8005, 8015

Rani, Neha: 1390

Rants, Jillian: 7350

Rao, Praveen: 9070

Rao, Smitha: 3125, 3305

Rappaport, Noa: 7090, 7190, 7315

Rapport, Lisa J: 7310

Rasmussen, Blake B: 8015

Rataj, Alison C: 2150, 4220, 7275

Ratangee, Brina: 7055

Rathbun, Alan M: 2000, 4330, 7315, 8010

Rathje, Elizabeth: 9095

Ratnayake, Maggie: 8015

Rattner, Edward: 7455

Rauer, Amy: 4490, 7355

Ravel, Jacques: 8010, 9150

Ravindran, Rohan: 8000

Rawal, Kadambari: 7485

Rawool, Mrinal: 8005

Rayan-Gharra, Nosaiba: 8005

Rayani, Asma: 7430, 7495

Rayner, Daniel: 7300

Raza, Sakeena: 7255

Razavian, Narges: 7185

Razjouyan, Javad: 8005

Reale, Kevin: 8015

Reardon, Eilis: 8005

Reaves, Alvin L: 3165

Reavis, Kelly: 8005

Rebadulla, Hannah M: 4350

Reblin, Maija: 2030, 7395

Rebok, George: 4030, 8000, 8005, 9040

Recker, Amy: 4475

Reckrey, Jennifer: 1270, 2480, 3315

Recto, Pamela L: 7255

Reczek, Rin: 1405

Reddy, Ann: 2055, 4475, 8015

Reder, Nora: 8005

Redfern, Mark: 8010

Redman, Leanne: 3267

Redmond, Leslie: 1120

Redondo-Sáenz, Diego: 7020

Reece, Heather: 8015

Reed, Alexandra M: 7185

Reed, Eric: 1520

Reed, Grace: 7220

Reed, Nicholas: 1075, 1200, 2000, 2535, 4065, 7330, 8005

Reeder, Blaine: 7085, 7910

Rees, Catherine: 7410

Reese, Jordan: 7475

Reeve, Charlie: 7230

Reeves, Alexis: 1110, 4460

Regal, Rachel: 1260, 2110, 7095

Regan, Claire: 8005

Regos-Stewart, Dalia: 7225, 7905

Reichman, Rocky: 4535

Reid, M. Carrington: 1055, 4010, 4135, 4525, 4535, 7350, 7500, 7540, 8000

Reider, Lisa: 4450

Reif, John S: 8015

Reiff, Jenni: 4450

Reiffer, Norina: 7250

Reilly, Elizabeth: 8000

Reina, Anita M: 8015

Reinecke, Robert: 4070

Reinke, Lynn: 7405

Reinschmidt, Kerstin M: 7415

Reisberg, Barry: 7010

Rejeski, W. Jack Jack: 2470

Rekant, Julie: 7140, 7905

Remenik-Zarauz, Vania: 8000

Remer, Sarah: 7500

Remillard, Elena T: 1230, 2430, 9110

Ren, Lily Haopu: 7205

Ren, Lily Haopu: 7235

Ren, Lily Haopu: 7915

Ren, Rong: 7470

Renn, Brenna N: 7375, 8005

Renold, Lowell: 8015

Renter, Heather M: 8005

Rentería, Miguel: 3380, 8010, 4540

Renzi-Hammond, Lisa M: 8015

Resendes, Natasha: 3065

Resnick, Barbara: 1260, 1435, 2180, 2350, 3160, 3220, 4460, 7290, 7495, 8005

Resnick, Susan: 4495

Reuben, David B: 2285, 3290

Reuveni, Yehudit: 8000

Revell, Andrew J: 7400

Reyes, Giselle: 2030

Reyes, Jimmy A: 2300

Reyes, Laurent: 1405

Reyes, Maria F: 7285, 7925

Reyes, Mariana: 7225, 7905

Reyes-Vega, Andrea: 8000

Reynolds, Addam S: 4455

Reynolds, Chandra A: 1175, 4030, 4110

Reynolds, Courtney: 1215, 7405, 8015

Reynolds, Lindsay M: 7480

Reynolds, Sarah R: 8005

Reza, Abigail: 2540

Rhee, Greg: 7410

Rhee, Min-Kyoung: 8015

Rhee, Soo: 4110

Rhodes, Alec: 3285

Rhodes, Annie: 1005, 7300, 8015

Rhodes, Ramona: 7220

Rhodes, Shanae: 7255, 9130

Ribeiro, Oscar: 2120, 7370, 7380

Rice, Laura A: 1230, 2430, 7225, 7410

Rich, Adea: 2000, 7435, 8000

Rich, Josiah: 1090

Rich, Scott: 3170

Richards, Emily: 8000

Richards, Kathy C: 4475

Richards, Marcus: 1105

Richards, Nicole E: 1175, 3185

Richards Adams, Ingrid K.: 2135

Richardson, Arlan: 4347

Richardson, James K: 7200

Richman, Katherine M: 4545

Richmond-Rakerd, Leah: 7255

Rickenbach, Elizabeth: 7355, 7515

Ricketts, I’deyah: 4285, 8010

Ridder, Becky: 7500

Riddick, Milan: 7470

Riddle, Nicole: 3397

Riegel, Barbara: 1015

Riffin, Catherine: 4135, 7500

Rijnhart, Judith J M: 7325, 7425

Riklon, Sheldon: 7005

Riley, Jessica: 3115

Riley, Thomas: 7335

Riley, Thomas: 7545

Rilling, James K: 1275

Ring, Lia: 1365, 7410

Ringel, Joanna: 1170

Riordan, Lindsay: 7470

Rios Casas, Francisco: 1105

Riosmena, Fernando: 4470

Rios-Monterrosa, Jose: 8005

Ripperger, Hayley S: 4585

Rippon, Isla: 4360, 8010

Risacher, Shannon L: 3380

Riser, Tiffany J: 1465, 2480

Risher, Jordan: 7455

Ristl, Christina: 1350

Ritchey, Katherine C: 1470

Ritchey, Katherine C: 4445

Ritchey, Katherine C: 7410

Ritchie, Christine S: 1160, 1270, 1400, 2190, 2285, 2310, 2480, 3375, 7185, 7205, 8000, 9120, 9150

Ritter, Ashley Z: 2465, 4010

Ritz, Thomas: 8010

Rivadeneira, Natalie A: 7145

Rivas, Daniel: 1520

Rivera-Hernandez, Maricruz: 3370, 7485

riveros, Angie F: 7285, 7925

Rizer, Sandra: 1100, 7095, 7415

Rizk, Christine: 8005

Rizk, Ryan C: 4560

Rizvi, Anita: 4190

Rizzo, Matthew: 7465

Rizzo, Samantha: 8000

RIzzo, Victoria M: 7485, 9030

Roan, Carol: 4585

Robbins, Rebecca: 7390

Robelly, Blade: 8000

Roberson, Donna: 7010, 8000

Roberson, Lauren: 8010

Robert, Benoît: 7370

Robert, Stephanie: 4520

Roberto, Karen A: 4520, 7230

Roberts, Adam: 7020

Roberts, Amy R: 2325, 7335

Roberts, Emily: 2290

Roberts, Eric T: 1250

Roberts, J. Scott: 4360, 4390, 7250, 9070

Roberts, Jimmie E: 7345, 7510

Roberts, Mitchell: 7320

Roberts, Pamela: 9020

Roberts, Patrick: 4007

Roberts, Shelby: 9030

Roberts, TJ: 1325

Roberts, Tonya: 7370, 8005

Robertson, Mariah: 3240

Robinet, Lauren J: 8015

Robinette, Jennifer W: 7315, 7900

Robins, Diana L: 4525

Robins, Richard: 4110, 8015

Robinson, Anthony: 7310

Robinson, D’Artagnan M: 7225, 7905

Robinson, Kat: 4355

Robinson, Marc: 1090

Robinson, Max: 7090, 7315

Robinson, Millicent: 4250

Robinson, Stephanie A: 7265

Robinson, Tyler D: 8005

Robinson-Lane, Sheria G: 1510, 2375

Robison, Anna: 8000

Robison, Julie: 1315, 1385, 2185, 4135, 4160, 7305, 7360, 9090

Robison, Kristel: 8005

Robson, Mei W: 8000

Roche-Dean, Maria: 1150, 1515, 2225, 2375, 7250

Rockenstyre, Alex: 8015

Rockwood, Kenneth: 7090

Rodakowski, Juleen: 1510, 7490

Rodgers, Mattea: 8000

Rodin, Rebecca: 7010

Rodney, Tamar: 4265

Rodríguez-Díaz, Melina: 7040

Rodrigo, Hansapani: 7190

Rodriguez, Amy: 8005

Rodriguez, Bianca: 8005

Rodriguez, Jamila: 8015

Rodriguez, Miriam Jocelyn: 2555, 3010, 3085, 7010

Rodriguez, Paola: 8010, 8015

Rodriguez, Racheal: 7125

Rodriguez Colmenares, Nury Alejandra: 4140

Rodriguez Cruz, Nachalie: 7300

Rodriguez-Cruz, Nachalie: 7130, 7185, 7225, 7905

Rodriguez-Morales, Grisel: 9140

Roe, Laura: 1135

Roecke, Christina: 4270

Rogers, Anita: 7470

Rogers, Geoff: 1085

Rogers, Wendy A: 1165, 1320, 1505, 2430, 4080, 7380, 7410, 8005, 8015, 9065, 9085, 9110

Rogus-Pulia, Nicole: 7415

Roh, Daniel: 4275

Roh, Soojin: 8015

Roh, Soonhee: 7160, 7375, 7415

Rohlfsen, Leah: 4510

Rohrs, Jennifer: 7415

Rojas, Ashley: 7345

Rojas, Elizabeth: 9040

Rojas, Olivia: 7380

Rojas-Alvarez, Alfonso: 1250, 2060

Rojas-Martinez, Julio: 9000

Rojas-Saunero, L Paloma: 4280

Rollandi, Isabel: 8010, 8015

Roller, Juliana: 8005

Rollings, Kimberly: 1375

Rom, Yifat: 3200

Roma, Corinne: 2055, 4010, 4475

Romañach-Álvarez, Lourdes S: 2345, 7145

Roman, Anna: 3295

Roman, Cristina AF: 8015

Roman Jones, Joanne: 4480

Roman-Woo, Carly: 1240

Romero, Jonathan: 8015

Romero, Sergio: 2445

Romero Aliberti, Marlon Juliano: 4425, 8005

Rominger, Marielle: 8005

Rompilla, David B: 2095, 7265

Rondina, Mathew: 8000

Rong, Jian: 8000

Ronis-Tobin, Victor: 8015

Rook, Erin: 1000

Rooks, Ronica N: 8005

Roque, Nelson A: 2400, 4310, 7380

Rorai, Vanessa O: 2515

Rosano, Caterina: 7425

Rosario, Andre A: 3205

Rose, Dorian: 8010

Rose, Grace: 7265

Rose, Johnie: 7220

Rose, Karen: 4170, 8005

Roseen, Eric: 4535

Rosen, Allyson: 7135

Rosen, Amy: 4090

Rosen, Howie: 7135

Rosen, Jennifer: 4160

Rosen, Tony: 1085, 2325, 3300, 8005, 9140

Rosenberg, Mara: 2190

Rosenberg, Walter: 9145

Rosenbloom, Michael: 8000

Rosenfeld, Anne: 7340, 7920

Rosen-Lang, Yael: 7000

Rosenstein, Benjamin: 3240

Rosenthal, Ronnie: 7500

Rosko, Ashley: 2220

Ross, Clifford: 1140, 7055

Ross, Jaime M: 1520

Ross, Jenifer: 4095

Ross, Lesley A: 1285, 2280, 3060, 4310, 7325

Ross, Sarah: 2065

Rossano, Samantha: 7415

Rosselli, Monica: 2045

Rosser, Mary: 7095

Rossi, Martina: 8000

Rossi, Michelle I: 3115, 7410

Rossi, Raiza: 8000

Rossler, Aleatha: 7330

Rossler, Aleatha M: 8005

Rosso, Andrea L: 2200, 9110

Rostamvand, Maryam: 7245

Rote, Sunshine: 2060, 3265, 7350, 7475, 8000

Roth, Nicole: 8015, 9155

Rothenberg, Craig: 8015, 9140

Rothermund, Klaus: 1210, 2370, 4270, 7390, 8015, 9070

Rothgang, Heinz: 1310

Rothlein, David: 8005

Rottenberg, Yakir: 8015

Rotter, Jerome: 8005, 9155

Roumell, Elizabeth: 8010

Rouse, Lorna: 3080

Routh, Brianna: 7265

Rovianne, Rovianne: 4015

Rovine, Michael J.: 7305

Rowan, Noell L: 8000, 8015

Rowe, Cathy: 3000

Rowe, Jeannine M: 7485

Roy, Arkaprava: 7350

Roy, Brita: 8000

Roy, Deepa: 7190

Roy, Indrakshi: 2260, 8005, 8010, 8015

Roy, Upal: 7190

Royse, Sarah K: 2200

Rozani, Violetta: 8005

Rozenberg, Aliza: 1155

Ruan, Jia Yin: 8005

Ruan, Lei: 7410

Ruan, Yunchen: 2275, 7470

Rubi, Lisbeth: 2220

Rubin, Leah: 1155, 7245

Rubio, Olivia C: 4445

Rubtsova, Anna A: 2395, 9080

Rudolph, James L: 9080, 9090

Rueppel, Elizabeth: 7290

Ruggiano, Nicole: 8010

Ruggles, Sydney C: 7300

Ruiz, Diana: 8010

Ruiz, Eloy F.: 8010

Ruiz, Jessica J: 7005

Rule, Andrew: 7325

Rule, Payton D: 2155

Runkle, Tage: 8005

Runyan, Amanda: 7010

Rupprecht, Fiona S: 1210

Rush, Jonathan: 1175, 2095, 2280, 4120

Rush, Kathy: 7485

Rusmevichientong, Pimbucha: 8015

Russell, Daniel: 4195

Russell, Marcia M: 7500

Russell, Wynfred: 2010, 3155

Rustici, Craig M: 3040

Ruth, Terrance: 7290

Rutt, Joshua L: 4460

Rutter, Lauren A: 1095, 9030

Ryan, Alice: 7130, 7265, 8010

Ryan, Candace EM: 8010

Ryan, Gery W: 3290

Ryan, Kathleen: 9075

Ryan, Lindsay: 1105

Ryan, Susan: 4480, 8010

Ryan Baumann, Mary: 1200, 8015

Rydberg, Michael: 1235

Ryder, Priscilla T: 8010

Ryff, Carol D: 4000, 7055

Ryou, Yeon Ji: 1065, 4195

Ryskina, Kira L: 7270

Ryu, Byeongju: 1195, 7290

Ryu, HyeRim: 7330, 7390

Ryu, Jungsu: 7265

Ryu, Seulgi: 7305, 8005

Ryvicker, Miriam: 7220, 8005

Rzepa, Natalia: 9085

Rzhetsky, Andrey: 4200

S

Sa, Sharon: 8005

Saad, Odelyah: 8005

Saalim, Khalida: 7530

Saar-Ashkenazy, Rotem: 2105

Saavedra, Joey M: 3045

Sabatini, Serena: 1210, 2390

Sabbatini, Amber K: 8000

Sabol, Valerie: 4075

Saccà, Valeria: 7435

Sacco, Lawrence B: 7375

Sacco, Paul: 1145, 7220

Sacks, Frank: 4570

Saczynski, Jane: 2505, 7410, 7485

Sadarangani, Tina: 2440, 4145, 8005

Sadeq, Nasreen: 7340, 7935

Sadhwani, Anjali: 8010

Sadler, Richard C: 2400

Sadow, Sage: 2030

Sadowska, Karolina: 7500

Sadowski, Martin: 7010

Saenz, Joseph L: 1495, 4290, 9075

Safford, Monika M: 1170, 7260

Sagehorn, Mallory A P: 7465

Sager, Jeannette: 8015

Sahai, Arjun/Aarti: 8010

Sahli, MIchelle: 7295

Sahoo, Nirakar: 7190

Sahoo, Sudikshya: 8010

Sailor, David J: 3235

Saini, Geetanjali: 3095

Saint Hamilton, Sarah: 7490

Sainz, Mayra: 1250, 3375, 9025

Saito, Taeko: 8010

Saito, Yasuhiko: 7235, 7390

Sakakibara, Sakura: 4370

Sakata, Nobuo: 8000, 8010

Sakaue, Hiroshi: 8015

Sakharov, Maya: 8015

Saksena, Mallika: 7295

Salaam, Donna: 7185, 8015

Salas, Sarah S: 3265

Salat, David: 7325

Salat, David: 8005

Salazar, Delany T: 4280

Saldana, Santiago: 8000

Salecha, Juhi Digvijay: 1110, 7495

Sales, Wesley: 8005

Saliba, Debra: 1270, 1435

Saligan, Leorey: 8015

Salinas, Joel: 8010

Salinas, Sophia E: 8000

Salisbury, Amy L: 1260

Salisu, Margaret: 8000

Salive, Marcel: 1455

Sallings, Abigail: 8015

Salomon, Amit: 7200

Salter, Allison: 7305

Samaniego, Catherine H: 4225

Samant, Akanksha: 8015

Sambataro, Franco E: 9140

Sames, Carol: 4525

Samper-Ternent, Rafael: 4565, 8000, 8005

Sample, Lola: 3085, 7415

Sampson, Laura: 7450

Samuel, Laura J: 1465, 3345, 4125

Şan, Helin Sera: 8005

San Roman, Irene: 1305

Sanchez, Emilia: 8010

Sanchez, Jessica: 7330

Sanchez, Victoria A: 4065

Sanchez, Victoria: 7335

Sanchez Concepcion, Laura: 7350

Sanchez-Reilly, Sandra: 8000, 8010

Sanchez-Reilly, Sandra: 8010

Sanderlin, Ashley: 8005

Sanders, Alexandra: 7360

Sanders, Alisha: 7360

Sanders, Camilla: 9035

Sanders, Edie C: 3025, 3215, 4420

Sanders, Lisbeth: 8000

Sanders, Margaret: 4600, 7225, 7340, 7920

Sanders, Natalie: 7030

Sanders, Tammy: 8010

Sanders, Wade M: 7345

Sandiford, Dione: 3205

Sandler, Dale: 1500

Sandoval, Moises: 9125

Sandridge, Desirae R: 7200

Sands, Laura P: 7095, 7170, 8000, 8015

Sanford, Jon A: 1230, 1320, 2430

Sang, Dongxia: 2325

Sang, Elaine H: 9135

Sang, Wei: 7430

Sangal, Rohit: 7485

Sangalang, Cindy: 7055, 7900

Sangdahl, Caitlin: 1135

Sanjanwala, Harshin: 7325

Sankar, Sudha: 8010

Sanner, Haley: 1000, 9130

Sano, Emily: 8010

Sano, Mary: 9080

Santamaria-Ulloa, Carolina: 4150, 7090

Santana, Layla Katharine: 7040, 7420

Santana, Paula: 7370

Santiñaque, Florencia: 9005

Santilli, Josie: 7105, 7215

Santomauro, Damian: 7185

Santora, Kenneth: 3033, 4008

Santos, Alexis: 4290, 8000

Santos, Giselle L A B: 7090

Santos, Suzanne T: 7370

Santostefano, Christopher M: 8000

Saper, Robert: 4535

Sapkota, Hardik: 1500

Sapkota, Krishna Prasad: 4465

Sapkota, Sabitri: 8015

Sapoznick, Ariella: 8005

Sappington, Erica: 7320

Sapra, Mamta: 7230

Saptono, Andi: 2375

SAQUING-NWODO, SHALLEE: 1050

Sarabia, Josie: 3305

Saraiya, Manali: 4135, 7500

Sarasty, Oscar: 7130

Saravanan, Anitha: 1505

Sardina, Angie L: 3060

Sargent, Lana: 1260, 7340

Sarig, Jonathan: 3040

Saripalli, Sree N: 8005

Sarkar, Antara: 7340

Sarkar, Nilanjan: 7545

Sarter, Katharina: 1005

Saruhanlioglu, Zeynep N: 1480

Sasscer, Cathy: 9100

Sasser, Janet: 1130

Satake, Anna: 7085, 7910

Sathyan, Sanish: 4495, 8015

Sato, Ashida: 1360

Saunders, Pamela A: 2070

Savage, Cary: 7325

Savard, Grace M: 2330, 7300, 7465

Savela, Roosa-Maria: 3030

Savla, Jyoti: 7230, 8010

Savoye, Louise: 4135

Saxena, Vivek: 8005

Sayer, James: 4435

Scaccabarrozzi, Luis: 2395

Scales, Kezia: 1005, 2225, 7075

Scanteianu, Adriana: 7535

Scerpella, Danny L: 2520

Schütz, Philipp: 8015

Schüz, Benjamin: 3035

Schaefer, Adam M: 8015

Schaefer, Sydney Y: 7185, 7315, 7325

Schafer, Donna E: 7075

Schafer, Markus H: 1105

Scheel, Alpha T: 8015

Scheffer, Julian A: 4085, 7290

Scheffers-Barnhoorn, Maaike N.: 4105

Schein, Richard M: 7495

Scheipers, Merle: 9070

Schell, Brent R: 7265

Schellhorn, Abby: 7220

Schenker, Rony: 1515

Schepens, Stacey: 9090

Scher, Clara J: 2185, 4145, 4530

Scherf, Erick: 3245

Scheunemann, Leslie Page: 2445

Schickedanz, Heather: 4160

Schiller, Gabrielle: 9145

Schilling, Birgit: 4473

Schilling, Oliver K: 8000, 8015

Schiloski, Kylie A: 7115

Schiloski, Kylie: 8010

Schilstra, Christina: 8010

Schiltz, Nicholas: 1485, 7220, 7410

Schindel Martin, Lori: 7075

Schlag, Karen E: 7230, 7405, 9025

Schlenk, Elizabeth A: 8010

Schlomann, Anna: 3215, 7295

Schluter, Emma: 4440

Schmeler, Mark R: 7495

Schmid, Arlene: 9090

Schmidt, Athina L: 7245, 7915

Schmidt, Cindy: 9000

Schmitt, Eva M: 2505

Schneeweiss, Sebastian: 7510

Schneider, AnnMarie: 3215

Schneider, Stefan: 2120

Schneider, Sue: 4075

Schnock, Kumiko: 8015

Schnyer, Rosa N: 7230, 7385, 7910

Schoberer, Daniela: 1310

Schoen, Gena: 0

Schoenberg, Nancy E: 4045

Schoenborn, Nancy: 1360, 2520, 7010, 7910

Schoenfeld, Elinor R: 4200

Schoenfield, Elinor: 3155, 4200

Schoenthaler, Antoinette: 1505, 7355

Schoepke, Abbie: 8005

Scholes, Shaun: 1040, 2250

Scholler, Emily: 9010

Scholz, Markus: 8015

Schork, Nicholas J: 4495

Schrack, Jennifer A: 1075, 2100, 2480, 3105, 3260, 4375, 4450, 7330, 9025

Schreiner, Pamela: 8005

Schrimpf, M. Brooke: 7125

Schroepfer, Tracy A: 2265

Schuler, Emily: 7055

Schulman, Daniel: 8000

Schulman-Green, Dena: 3015, 3290

Schultheis, Maria: 7290

Schultz, Amy: 7260

Schultz, Douglas: 7325

Schultz, Emma: 7470

Schultz, Sydney M: 2100

Schulz, Cynthia L: 8005

Schumacher, John G: 4007

Schurch, Thomas: 1390

Schuster, Amy M: 3340

Schuster, Bernard G: 8010

Schutte, Debra: 8010

Schuttner, Linnaea: 4445

Schutz, Jennifer: 7455

Schwarcz, Lily: 7545

Schwartz, Andrea Wershof: 2195, 8005

Schwartz, Andrea Wershof: 7090

Schwartz, Beth: 8010

Schwartz, Heather M: 7305

Schweikhard, April: 2445

Schwendeman, Hannah: 1190

Schwieters, Katelyn: 8010

Schwoyer-Morgan, Tracie: 7500

Sciamanna, Christopher N: 8005

Scott, Don: 8015

Scott, Kim: 9000

Scott, Matthew: 8010

Scott, Paul W: 7500

Scott, Stacey B: 1175, 4473, 7255

Scott, Stephen: 8005

Scott, Winifred: 9095

Seabury, Joshua: 4555

Seago, Elayna: 7095, 7200, 8005

Seah, Betsy: 4425

Seale Reiff, Jenni: 1360

Seaman, Aaron: 1305, 8000

Sebastiani, Paola: 1520, 4440, 7310, 8005, 8015, 9155

Sedaghat, Sanaz: 8015

Seegmiller, Jesse: 7010

Sefcik, Justine S: 7295, 7360, 7415

Segal, Daniel L: 7505

Segal-Gidan, Freddi: 4160, 7135

Segel-Karpas, Dikla: 1125, 3040, 4185

Segev-Rosenberg, Galia: 1090

Segrè, Daniel: 8015, 9155

Segundo, Joahana: 1440

Sehgal, Raghav: 9060, 9150

Seidel, Elizabeth: 2015

Seidenfeld, Justine: 2475, 4565

Seino, Satoshi: 7265

Sekhon, Vishaldeep: 8000, 8005, 8015

Seldeen, Kenneth L: 8010

Seliger, Stephen L.: 7345

Seligman, Benjamin: 8000

Sellers, Ashley J: 2255

Selovin, Fabio: 8015

Seltzer, Judith A: 3050

Selvin, Elizabeth: 2100

Semba, Richard: 7315

Semedo, Ana: 9100

Semelka, Charles T: 1455

Semple, Benjamin J: 9095

Semple, Paige: 1385

Senderling, Ben: 8005

Senior, Joshua: 8005

Senyk, Oksana: 3360, 4270

Seo, Hyein: 8010

Seo, Jeongone: 7295, 7535

Seo, Jin Young: 7255

Seo, Jisu: 7150, 8005

Seong, Minju: 7355, 7470

Seow, Lek Wei: 7190

Sergile, Jennifer: 7245, 7915

Serier, Kelsey: 2085

Serina, Peter: 2055, 8015

Serpas, Dylan G: 7145

Service, Kathryn: 1395

Seshadri, Gokul: 2345, 7345

Seshadri, Sandhya: 3165

Seshadri, Sudha: 3127, 8015

Sethi, Reena: 9120

Setiawan, I Made Agus: 2375

Setoguchi, Soko: 8010

Severance, Jennifer: 2065, 7540

Severine, Janet: 8010

Sevi, Barış: 3265, 4110

Sevilla, Javier: 8010, 8015

Seward, Morgan: 4590

Sexton, Michelle: 8015

Shaaban, C. Elizabeth: 2200, 3380

Shabbir, Alibhai: 7090

Shachaf, Hadas: 7060

Shade, Marcia: 8005

Shadid, Jamileh: 7185

Shadmi, Efrat: 7000, 7145

Shaffer, Tasha: 7265

Shafiekhani, Parsa: 7295

Shah, Amil: 3185

Shah, Amrik: 8010

Shah, Avani A: 8010

Shah, Raj C: 8005

Shah, Ravi: 2245, 7345

Shah, Sachin: 4280, 7185

Shah, Samantha: 2555

Shah, Yogi: 7185

Shaham, Galia: 7200

SHAHAR, Danit R: 7135

Shahar, Orit: 1515

Shahriar, Kamal Md: 7480

Shaikh, Afroze N: 7375

Shakir, Madiha: 7010

Shakya, Iju: 7510

Shaleha, Rinanda RA: 7380

Shalev, Daniel: 4135, 4225, 7020, 7500

Shalev, Idan: 1375

Shamsi, Masooma: 1505

Shan, Liang: 4140

Shan, Shengshuai: 8015

Shan, Yong: 1070, 7305, 8005

Shanahan, Angela: 7225

Shanbhag, Prajakta: 7290

Shanbhogue, Nikhil M: 2350, 7525

Shane, Jacob: 1175

Shang, Lili: 7515

Shannon, Faith: 1175, 8000

Shao, Gracie Wan Ting: 7535

Shao, Hsuanlei: 7465

Shao, Lu: 1245, 4055

Shao, Sijing: 4120

Shao, Yijun: 8010, 9020

Shao, Yongzhao: 7010

Shapira, Bettina: 7175

Sharafieh, Roshanak: 9090

Sharafkhaneh, Amir: 8005

Sharafkhaneh, Sanam: 8005

Sharbaugh, Michael B: 1385

Shardell, Michelle D: 2000, 3260, 4330, 7315, 7345, 7495, 8010, 9075, 9150

Sharifi, Hasti: 4590

Sharit, Joseph: 4080

Sharma, Anajali: 2395

Sharma, Nicole: 7545

Sharma, Shakshi: 7005

Sharma, Tanya: 7150

Sharma, Uttam: 1500

Sharp, Meaghan: 1015

Sharrett, A. Richey: 8010

Shaughnessy, Marianne: 1470, 2195

Shavit, Yochai Z: 3275, 8005

Shaw, Albert C: 8000

Shaw, Benjamin A: 7375, 7395

Shaw, Clarissa: 1150, 1515, 2225, 3220, 7000, 7250

Shaw, Susan: 7110

Shawley, Audrey: 7320, 7510

Shayya, Ashley: 7220, 7330

Shdo, Suzanne M: 1275

Shea, Kyla: 4295, 4440, 4460

Shealy, Carlisle: 7170

Shean, John: 7010

Sheehan, Cailee: 3150

Sheehan, Orla C: 2480, 7385

Sheets, Nathan: 8000

Shefer-Hilel, Galia: 7135

Sheftel, Mara G: 1405, 4290, 8015

Shehu, Erblin: 7070, 7300, 7930

Sheikh Ibrahim, Hanan: 9015

Sheikhnur, Sahra: 8015

Shelar, Ashish: 8000

Shelawala, Nicole: 4465

Sheldon, Kira G: 7220, 8000

Sheldon, Marisa: 3305, 9110

Shellman, Juliette: 7410, 7525

Shelton, Amy: 7000

Shen, Huei-wern: 7005, 7100, 7375

Shen, Jie: 1245, 7090

Shen, Karen: 3315

Shen, Lingyi: 9055

Shen, Megan: 8000

Shen, Yuqi: 7535

Shenoy, Rhea: 8010

Shepherd, Alexis: 7305

Shepherd-Banigan, Megan: 4565, 7350, 7930

Sheps, Janna: 1485

Sherk, Catherine: 8000

Sherman, Carey Wexler: 2010

Sherman-Wilkins, Kyler J: 2345

Sherry, Laura: 4065

Sherwood, Bryan: 8005

Shetty, Nihar N: 1470

Shewokis, Patricia A: 4525

Shi, Bertram E: 8005

Shi, Cheng: 8015

Shi, Chunhu: 9085

Shi, Hui: 9030

Shi, Huidong: 7245

Shi, Jingwen: 7320

Shi, Keyi: 8005

Shi, Sandra: 9000, 9080, 9135

SHI, Xiaoming: 8005

Shi, Xiaotian: 7315

Shi, Yifan: 8000, 8010

Shiba, Koichiro: 1195

Shields, Kerry: 8000

Shieu, Bianca: 2255, 8000, 8005

Shiferaw, Tsion: 8015

Shih, Ludy C: 1510

Shih, Regina A: 2160, 2375, 4160

Shih, Yao-Chi: 7250

Shillingsburg, Laura: 2340

Shim, Minji: 7330

Shimabukuro, Michio: 8015

Shimamoto, Tomonari: 2230

Shimizu, Natsumi: 8010

Shimokata, Hiroshi: 7330

Shin, Dong-Soo: 8005

Shin, hyeri: 9065

Shin, Hyeyeon: 8015, 9030

Shin, Jiyoung: 2210

Shin, Juh: 7245, 8010, 8015

Shin, Jung-hye: 2445

Shin, Oejin: 8005, 8010

Shin, So Young: 7370

Shin, Soo Gyung: 8010

Shin, Sueun: 8005

Shin, Sunui: 7425

Shin, Yeonsoo: 7090, 7165

Shin, Yoonkyung W: 1415

Shinbeckler, Rachael: 8015

Shinozaki, Gen: 8005

Shiovitz-Ezra, Sharon: 4315

Shippee, Tetyana P: 1010, 1430, 2010, 2160, 2260, 3020, 3075, 3155, 3190, 7270, 7300, 7530

Shiraishi, Natsue: 7380

Shireman, Theresa: 8015

Shirk, Steve: 7230

Shirk, Steven D: 1305, 2365, 7280, 7295, 7310

Shirley, Kate: 8010

Shirouchi, Mana: 7440

Shiu, Cheng-Shi: 1490, 7245

Shivanand, Ishan: 7340

Shivers, Stephani: 1160, 1225

Shmidt, Anastasiia J: 8010

Shmuels, Diego I: 8000, 8015

Shobugawa, Yugo: 7345

Shochat, Tamar: 4335, 7255

Shomee, Homaira Huda: 4590

Shorer, Eran: 1155

Short, Meghan I: 8015, 9155

Shovali, Tamar: 2330, 4355

Shrestha, Aman: 4465, 7345

Shrestha, Jene: 8015

Shrestha, Priyanka: 7060

Shrestha, Sabita: 8005

Shrestha, Srijana: 8015

Shrestha, Srishti: 2535, 7325

Shrikanth, Shalini: 1505

Shrira, Amit: 3360, 4270, 8005, 8015

Shrivastava, Jyotsna: 7190

Shrivastava, Mansi: 7480

Shroff, Jay: 7485, 7930

Shuber, Florene: 7340, 7920

Shu-Fen, Wung: 8005

Shugaev, Inna: 1090, 7450

Shukla, Jayanti: 7495

Shukla, Rohit: 7190

Shulyaev, Ksenya: 7000, 7450

Shuman, Valerie: 8005

Shvedova, Maria: 4275

Si, Wanwan: 1090

Siamdoust, Shahrzad: 2475, 4145

Sibai, Abla Mehio: 1495

Sicbaldi, Marcello: 9020

Sicinski, Kamil: 4585

Siconolfi, Daniel: 4160

Sicotte, Nancy: 9020

Siddiqui, Aziza: 7160

Siddiqui, Rouida: 7440

Sideman, Alissa: 7135

Siebecker, Megan: 3080

Sieber, Frederick: 2510

Siedlecki, Karen: 3200, 7040

Siefkas, Anna: 9155

Siegel, Bryn: 7435

Siegfried, Alexa L: 4435

Siegler, Eugenia L: 1490

Siepker, Kathy L: 7390

Siepker, Kathy: 8015

Sifnugel, Natalia: 7090, 7485, 8015

Silberbogen, Amy: 7265

Silbert, Lisa C: 1185, 2360

Silvani, Alessandro: 9020

Silveira, Erika Aparecida: 8000

Silveira, Maria: 4450

Silver, Michelle: 8015

Silver, Roxane Cohen: 7050

Silverberg, Michael: 7110

Silverblatt, Dana: 2110

Silverman, Sydney: 8015

Silverstein, Merril: 1225, 2405, 4040, 4315, 7085, 7375, 7470

Silverstein, Nina M: 1130, 2145, 3080, 3170, 4555, 7170, 7305

Sim, Jin-Ah: 8005

Sim, Xueling: 8005

Simard, Pascale: 8005

Simbrig, Ines: 3215

Siminerio, Linda M: 8015

Simon, Lena: 3060

Simonds, Wendy: 2225

Simonian, Melissa: 2055, 4475

Simonini, Sean: 8000

Simonsick, Eleanor: 2000, 2100, 2245, 4008, 7345

Simpson, Darcia: 7340, 7920

Simpson, Deborah: 7455

Simpson, Elizabeth: 1150, 3335, 7250, 7255

Simpson, Gaynell M: 3055

Simpson, Kit: 9035

Simpson, Michelle: 4205

Simpson, Stephen: 4347

Sims, Kendra D: 4540, 7260

Sin, Mo-Kyung: 2460, 3065, 9020

Sin, Nancy L: 1480, 8005

Sin, Wilfred Wing Fung: 7085

Sindi, Shireen: 7375

Singer, Daniel E: 7510

Singer, Dianne C: 4390, 7250, 9070

Singer, Jonathan: 4590

Singer, Kate: 2335, 3135, 4160, 7330, 7540

Singer, Neta: 4205

Singh, Ganeev: 7510

Singh, Nandini: 4020

Singh, Navrag: 2435

Singh, Portia: 4305

Singh, Ramkrishna Kumar: 8015, 9015

Singh, Roshni: 8010

Singh, Sangeeta: 8015

Singh, Shifali: 1295

Singh, Swastik: 1520, 7480

Singh, Yashaswini: 3280

Singh Wadhwa, Satpal: 2555

Singleton, Mekiayla (Meki): 2140, 7220

Şinikçi, Ceren: 8005

Sinkewicz, Marilyn: 1370

Sinvani, Liron: 2175

Siren, Anu: 2350

Sirey, Jo Anne: 2220, 8010, 8015

Siriprakorn, J. Peter: 8000

Sirois, Lynette: 8015

Sisco, Shannon M: 4570

Sison, Cristina: 8000

Sista, Anushka: 4210

Siu, Albert: 8000

Sixsmith, Andrew: 1325

Sixsmith, Judith A: 1135, 1325, 8015

Sjöberg, Maja: 4010

Skelton, Dawn: 9085

Skinner, Caleb: 7355

Skinstad, Anne Helene: 4350

Skipper, Antonius D: 2225, 4550

Skoblow, Hanamori F: 4485, 7085, 8000

Skrajner, Michael J: 8005, 9010

Skripka, Lindsey: 8005

Slachevsky, Andrea: 8015

Slade, Eric: 8000

Slade, Martin R: 2370

Slater, Craig: 4345

Slattum, Patricia: 1260, 2110

Slaven, James E: 8010

Slejko, Julia F: 8015

Sliwinski, Martin J: 1175, 1295, 1445, 4050, 4215

Sloan, Caroline E: 2075

Sloan, Richard P: 8005

Sloane, Philip: 7225

Slutsky-Ganesh, Alexis B: 8005, 9025

Small, Brent J: 8000, 8010

Small, Sarah: 2190

Smallwood, Stacy: 4240

Smerker, Nick: 4035

Smichenko, Juliana: 4335, 7000, 7060, 7255

Smies, Chad W: 7190, 8010

Smiley, Adrianne: 7280

Smith, Alex: 7140

Smith, Alexander k: 2190, 7010

Smith, Amanda: 8010

Smith, Anna P: 7405

Smith, Ashleigh: 3235

Smith, Ashley: 4475, 7105

Smith, Benton: 8005

Smith, Bethany: 7475

Smith, Brian N: 2085

Smith, Camille E: 7405

Smith, Eleanor: 7405

Smith, Emily L: 1120

Smith, Isaiah: 8000

Smith, Jacqui: 2050, 3175, 7305

Smith, Jamie M: 1270, 7485, 7930

Smith, Jason: 7330

Smith, Jason: 8010

Smith, Jeffrey S: 7190

Smith, Jennifer L: 1490

Smith, Jennifer L: 7110

Smith, Jennifer L: 7295

Smith, Jennifer: 7315

Smith, Jennifer L: 7380

Smith, Jennifer: 7900

Smith, Jennifer L: 8005

Smith, JoAnn: 2515

Smith, Joyce: 7495

Smith, Kasey N: 9065

Smith, Kimberley J: 4360

Smith, LaTonya: 7255, 7395

Smith, Lenora W: 8000

Smith, Lindsey: 3280, 4400, 8005, 8015

Smith, Matthew Lee: 1185, 2065, 4180, 7085, 7375, 8000, 8005

Smith, Mindy A: 7485

Smith, Rebecca: 7420

Smith, Sharla: 8010

Smith, Sherri L: 8005

Smith, Tamara: 9115

Smith, Terry E: 2170

Smith, Thomas J: 4030

Smith, Valerie: 8000

Smith, Valerie: 8005

Smith, Veronica: 9020

Smith, Wanda: 1015

Smith-Ray, Renae L: 1490

Smyles, Hanna: 8000

Snead, Caleb A: 8015

Sneed, Rodlescia S: 2390, 8000

Snitz, Beth: 1255, 8015

Snow, A. Lynn: 1060, 1300, 4475, 7105, 7310

Snyder, Alyssa: 9080

Snyder, Claire: 7545

Sobhie, Maria: 8010

Soble, Jason R: 3355

Socci, Marco: 7070

Sodders, Stephanie: 7410

Sodhi, Jasleen: 8000

Soh, Yenee: 1225

Sohmer, Dana: 2205

Sohn, Joanna: 8005

Sohn, Sunghwan: 9045

Soiron, Séverine: 7250

Sojib, Noushad: 4005

Sol, Ketlyne: 7465, 8005, 8010

Soliman, Germine: 4135, 8010

Solis, Guillermina R: 8010

Solomon, Adam: 8010

Solomon, Alina: 1290, 3030

Solomon, Philip: 7410, 9055

Solorzano-Salazar, Dustin M.: 8010

Solway, Erica: 4360, 4390, 7250, 9070

Somayaji, Darryl: 2255

Somerville, Ceara: 2185

Sompanich, Nussaba: 7375

Som-Pimpong, Hadassah: 8015

Son, Bo Ran: 3235

Son, Jiwon: 8015

Son, Surim: 7225, 7905

Song, Andrew J: 9070

Song, Eunah: 7525

Song, Jaehyuk: 8000

Song, Jieun: 7055

Song, Jiyoun: 8005, 8010

Song, Jun-Ah: 7525

Song, Jungwoo: 4420

Song, Kyungeun: 7395

Song, Lixin: 8000, 8005

Song, Mi-Kyung: 4135, 7350

Song, Minju: 8015

Song, Qian: 3320, 7170, 7210, 7300, 7305, 7375, 8015

Song, Rhayun: 7150, 8005

Song, Shixing: 7145

Song, Si Young: 1095

Song, Sunmi: 9060

Song, Weijing: 8000

Song, Woojin: 1160, 3355, 7015

Song, Xu: 7225

Song, Yajun: 8000

Song, Yeonsu: 1295

Song, Yuting: 4065

Song, Zachary: 7220

Song, Zeyuan: 8005

Soni, Albin: 7440

Sonnega, Amanda: 9040

Sorapuru, Candice: 8005

Sörensen, Silvia: 1290, 4175, 7145, 7380, 7925

Soriano, Christina: 8000

Sorkin, Clara: 7350

Sorkin, John: 7130

Soske, Jon D: 4010

Sosnoff, Jacob J: 4060, 7225

Sosnowski, David W: 7040

Sotres-Alvarez, Daniela: 7535

Sou, Ka Lon: 3090, 3215

Soulie, Brianna: 4245

Soundararajan, Srinath: 8010

Sousa, Liliana: 7370

Sousa, Susana: 7135

Southerland, Jodi L: 7085

Souza, Anita M: 8010, 8015

Sowan, Azizeh: 1135

Spadola, Christine: 7390

Spangler, Derek P: 8010

Spangler, Hillary B: 1220, 1235

Spartano, Nicole L: 8000

Spaulding, Erin M: 4385, 7545

Speckmann, Elisa-Marie: 7225

Spector-Mersel, Gabriela: 4315, 7450

Spees, Colleen: 2220

Spencer, Chelsea M: 7150

Sperber, Nina: 4430

Spetz, Joanne: 2260, 3315, 4260, 8010

Spira, Adam P: 1510, 2035, 3235, 4375, 7040, 8015

Spiro, Avron: 2085, 3330

Spiteri, Raymond J.: 7205

Splaine, Michael J: 8005, 8010

Split, Molly: 7290

Spooner, Kiara: 3180

Spradley, Laura: 7015, 7920

Sprecher, Anne: 9030

Springer, Shmuel: 1220, 2245

Springstein, Tabea: 1045

Sprong, Matthew: 8005

Spychalla, A.J.: 7025

Squires, PhD, RN, FAAN, Allison P.: 1485

Srivastava, Shobhit: 7040

St. Louis, Renee M: 3170, 4435, 7155

Stadler, Susi: 2515

Staehli, Alexandra: 7250

Staiger, Douglas: 8000

Staker, Erin: 4190

Staley, Mark: 8010

Stam, Elisabeth J: 1355, 7275

Standiford Reyes, Laurel: 7330

Stanfield, Jack E: 8000

Stanford, Ty: 3235

Stangl, Morgan: 7260, 7290, 7925

Stanley, Jennifer T: 7325

Stanley, Padraic: 9145

Stanmore, Emma: 9085

Stansbury, Kim: 7290

Stark, Susan: 1320, 1410, 2430

Starwalt, Susan: 3170

Staton, Ashley: 1260

Staton, Elizabeth W: 7140

Statz, Tamara: 7155

Staudacher, Sandra: 7250

Stavig, Mariana: 2555, 3010, 7010

Stavrinos, Despina L: 4570

Stawnychy, Michael A: 8000

Stawnychy, Michael: 9135

Stawski, Robert S: 1175, 2280, 7115, 7335

Stayer, Sarah: 7305

Steagall, Jill: 7410, 7910

Stearns, Melanie A: 4230

Stechuchak, Karen M: 1470

Steege, Linsey M: 4425, 7495

Steel, Ryan: 1190

Steele, Joel: 7335, 7375, 7415

Steele, Victoria: 7340

Stefano, Madeline: 8005

Stefanucci, Jeanine: 8000

Steffen, Ann M: 2240

Steffens, David C: 1385, 8005

Steinman, Bernard Alex: 4210, 7070, 7300

Steinman, Kenneth J: 3300

Steinman, Michael: 7255, 8010

Steinmann, Gijs: 3035, 7370

Steinmetz, Jaimie: 7185

Stelmach, Rachel D: 7530

Stenlund, Säde: 8000

Stensland, Meredith L: 7500

Stephan, Abigail: 1285, 1390, 3060, 7325, 8015

Stephan, Blossom: 1210

Stephan, Yannick: 1120

Stephen, Kritchevsky: 4525

Stephens, Andrew: 7415

Stephens, Caroline E: 1025

Stephens, Jacquelyn: 7265, 7380

Stephenson, Jane M: 8000

Steptoe, Andrew: 4095, 4120

Sterling, Madeline: 1170, 7440

Sternberg, Kady F: 9045

Sternberg, Shelley A.: 8015

Sterner, Elizabeth A: 1505

Sterns, Anthony: 8015

Sterns, Harvey: 7215

Stessman, Jochanan: 1155

Stessman-Lande, Irit: 1155

Stetten, Nichole A: 1465, 2055

Stevens, Alan B: 1035, 1225, 7475

Stevens-Lapsley, Jennifer E: 1435, 7140

Stevenson, David: 9090

Steward, Andrew: 8000

Stewart, Anne: 8010

Stewart, Brittany: 8010

Stewart, Wynnett C: 7245

Stickel, Ariana: 7535, 8005, 8010, 8015

Stigar, Lorraine: 4240

Still, Carolyn Harmon: 8010

Still, Michael: 4090

Stockdill, Macy: 8015

Stockton, Melissa A: 7530

Stoddard, Gregory J: 2190

Stodola, Allyson R: 4400

Stoeckle, Rebecca J: 2285

Stokes, Andrew: 4280

Stokes, Jeffrey: 1355, 1415, 2345, 3335, 4360, 7050, 7200, 7275, 7335

Stokes, Sandy Chen: 8005

Stokey, Max: 3305

Stolle-Wahl, Claudia: 1310

Stolterman Bergqvist, Erik: 4150, 8015

Stolzmann, Kelly: 4090

Stone, Elise: 1325, 8005

Stone, John: 2470

Stone, Katie L: 4385, 8000, 8005

Stone, Patricia: 7305

Stone, Robyn I: 4405, 4545, 7075, 7280, 7300, 7360, 8005, 9000

Stone, Samantha: 0

Stone, Sasheen T.: 7375, 7415

Stone-Bury, Lisa E: 7505

Stoop, Annerieke: 1010

Storms, Aaron D: 7220

Storrs, Cal: 8015

Stoumbos, Julia A: 3000

Stout, Michael: 4347

Stovrag, Selma: 1300

Stowe, Cynthia L: 2470

Strand, Bjorn Heine: 7415

Strand, Jennifer: 4010, 7255

Strangmann, Iris: 3380

Stratton, Lauren: 2030, 4160

Strauss, John: 2120

Street, W. Nick: 8015

Strickland, Larry C: 9025

Stringfellow, Mary: 8010

Stripling, Ashley: 7005, 7110

Strominger, Julie: 8000

Strommen, Jane: 2290

Strotmeyer, Elsa: 2245

Strough, JoNell: 1330, 3275, 7035

Strouse, John J: 7170, 7220

Strouse, Madison: 2085

Strunk, Sydney N: 4360, 4390, 7250, 9070

Strupeit, Steve: 7125

Stryczek, Krysttel: 8015

Stuart, Nicole S: 8005

Stuckenschneider, Tim: 4450, 7200, 7225

Stuifbergen, Alexa: 7420

Stull, Jennifer Denk: 7490

Stump, Timothy: 1145

Sturdevant, Diana L: 7005, 7370

Stusser, Aaron: 2085

Stutts, Whitney: 1220

Su, Chen-Chang: 7070

Su, Chin-Yi: 2050, 2530

Su, Jasmine IS: 7090, 8015, 9140

Su, Jin: 8005

Su, Linzhi: 7300

Su, Shaoyong: 2475

Su, Yan: 7185

Su, Yan-Jhu: 1075, 7080, 7200, 7210, 7275, 7305, 7375, 7435, 7530

Su, Yixin: 8015

Suamchaiyaphum, Krisada: 4225

Suandi, Matthew: 4245

Suanet, Bianca A: 8000

Suarez, Jethro Raphael M: 3030, 7225, 7545, 8005, 8010

Suarez Vargas, Kattia L: 2515, 7215, 7925, 8000

Suazo, Omar: 8010, 8015

Suberry, Assaf: 2370, 4395

Subramanyan, Sanjana: 1510

Sudore, Rebecca L.: 7220

Suero Samboy, Yudi Pamela: 7240

Sugar, Judith A: 7075

Sugawara, Ikuko: 7260

Sugiyama, Kei: 7330

Sugiyama, Mika: 7440

Suh, JungYeon: 3230, 7295

Suh, Michelle: 1090

Sui, Junyu: 4170, 8000

Suitor, J. Jill: 2405, 4040, 4185, 4315, 7085, 7470

Sukenova, Dinara: 7445

Sulick, Brenda A: 4400

Sullivan, Brooke: 7390

Sullivan, Campbell: 8015

Sullivan, Kevin: 1200, 2535, 7025, 7325, 7495

Sullivan, Shannon M: 7010

Sultana, Shabiha: 7245

Sumikawa, Yuka: 2210, 7440, 8000

Sun, Bangyao: 8000

Sun, Changqing: 2305

Sun, Dongmei: 4525

Sun, Fei: 1145, 2150, 2305, 3215, 7035, 7405, 7445, 8005, 8010

Sun, Haoqi: 7015

Sun, Haosen: 1490, 7355

Sun, Jany: 7485

Sun, Kaili: 9150

Sun, Kang: 7210

sun, lingxue: 7020

Sun, Nina: 7190

Sun, Peter C: 7100

Sun, Qin: 9115

Sun, Ruike: 8015

Sun, Tongxin: 8005

Sun, Tyler: 7300

Sun, Winnie: 2465

Sun, Xiaoxiao: 7500

Sun, Xin: 1205, 4055, 7405

Sun, Ying: 7090, 7315, 7320

Sun, Yu: 8000, 8010

Sun, Yue: 7465

Sun, Yue Yang: 3275

Sun, Yuzhou: 8000

Sundby, Jonathan M: 7020, 7195, 7900

Sunderaraman, Preeti: 1100

Sung, Pildoo: 3325

Sunil, Surya: 7025, 7195

Suo, Shujing: 7125

Supiano, Katherine P: 4240

Surachman, Agus: 9155

Surapaneni, Aditya: 1155, 3105

Suresh, Sharmila: 9145

Suriaga, Armiel A: 2275, 7020

Surood, Shireen: 1225

Susanto, Herry: 7475

Sutin, Angelina R.: 1120, 1265, 1445, 1480, 4110, 4360, 8015

Sutphin, George L: 4275

Sutton, Brad: 1255

Suwanno, Jom: 2435

Suzuki, Haruno: 9060, 9090

Suzuki, Rie: 7145, 7160

Svec, Joseph: 1120, 2305, 4315, 8015, 9140

Swaim, Kathryn E: 3095

Swain, Joseph: 8010

Swanger, Nancy: 4565

Swanson, Elizabeth A: 8010, 8015

Swanson, Helena L: 3150

Swartwood, Belise: 8010

Sweeney, Nicholas: 4240

Sweet, Jerry J.: 7330, 7390

Swenson, Rod: 4570

Swift, Samuel: 8000, 9120

Swint, Shelton G: 8010, 8015

Syed, Alisha: 7025

Sylvester, Hunter C: 2535, 7325

Syzf, Moshe: 8005

Szabo-Reed, Amanda: 7265

Szanton, Sarah: 1320, 2550, 3345, 4125, 4445, 7500, 8015

Szymkowski, Amanda: 7455

T

T, Muhammad: 2105

Tabaczynski, Allyson: 8000

Tabatabaei, Abbas: 7225

Tabb, Loni P: 7260

Tabloski, Patricia A: 7500

Taei, Afsaneh: 4215

Taglialatela, Giulio: 8010

Taguchi, Reina: 7250, 7465

Tahashilder, Md Ibrahim: 8010

Tahmaseb McCon, Jasmin B: 9100

Tahulending, Peggy: 8000

Tai, Albert: 1520

Tai, Joe: 8015

Tak, Sunghee H: 8015, 9125

Takahashi, Hideto: 8010

Takako, Unno: 7440

Takaoka, Manami: 2210

Takase, Mai: 7375

Takayama, Midori: 7260

Takeya, Yasushi: 7195

Taks Calle, Muriel: 2005, 7470, 7535, 7900, 8015

Talarczyk, Jarett: 4335

Talbot, Elizabeth Peffer: 7375

Talegawkar, Sameera: 4295

Talkar, Tanya: 8000

Talley, Kristine: 2010

Talwar, Sonia: 8010

TAM, Wai San Wilson X: 7040

Tamiya, Nanako: 8000, 8010, 8015

Tan, Alai: 8010

TAN, Davynn G. H.: 4455

Tan, Jing Xuan: 4370

Tan, Kok Yang: 2435

Tan, Linda Wei Lin: 8005

Tan, Micah: 8000

Tan, Minghui: 8015

Tan, Min-Han: 8005

Tan, Nasya S: 1075

Tan, Poh Choo: 7040

Tan, Wen-Hann: 8010

Tan, Zaldy S: 8010, 9020

Tanabe, Hayato: 8015

Tanaka, Keiko: 3010

Tanaka, Toshiko: 4295, 4495, 9155

Tang, Charlotte: 7160

Tang, Fengyan: 4070, 4505, 7090, 7260, 7435

Tang, Mandi: 7295

Tang, Siyuan: 8015

Tang, Tiffany: 7375

Tang, Vera Mun Yu: 1030

Tang, Vivien: 7395

Tang, Wen: 7090, 7315, 7320

Tang, Yongfang: 4105

Tang, Yunping: 7435

Tange, Chikako: 7330

Tanglai, Wirampa: 4225

Tangonan, Andrea A: 1230

Tangpricha, Vin: 7110

Tangredi, Kaitlin: 8000, 9055

Tanguay, Adam: 8000

Tanide, Atsuko: 7265

Tanner, Gwen: 7075

Tanner, Jeremy A: 8015

Tappen, Ruth M: 2045

Tarabochia, Dawn: 7265

Tarbox, Nathan: 8005

Tarraf, Wassim: 1370, 2040, 4570, 4580, 8000, 8005

Tasnova, Sabrina: 7125

Tatar, Raquel G.: 4590

Tate, Channing E: 8015

Tate, Judith A: 7545, 8000

Tate, Rio S: 8010

Tateishi, Mana: 7330

Tateyama, Yukiko: 2230

Tauseef, Mahrukh: 7545

Tavares, Karina: 8000

Tay, Djin: 1025

Taylor, Catherine: 7170

Taylor, Harry O: 2360

Taylor, Janelle S: 2420

Taylor, Janiece L: 2030, 3205, 7420, 7500

Taylor, Melissa L: 8010, 9140

Taylor, Morgan K: 7200

Taylor, Nathan: 7285

Taylor, Olivia: 2055

Taylor, Philip: 1005, 3335, 4550

Taylor, William: 2435

Taylor-McPhail, Tobie E: 4090

Tchatlian, Lilianna: 7415

Teasdale, Thomas: 8000

Teaster, Pamela: 3350, 4565, 7170

Teerawichitchainan, Bussarawan Puk: 2275, 7150

Teixeira, Laetitia: 2120, 7135, 7380

Teixeira, Samantha: 3305, 4530

Teixeira-Santos, Ana C: 8000

Teles, Sheila: 8000

Tell, Eileen J: 4545, 7085

Telonidis, Jacqueline S: 4240, 8010

Teng, Chiao-Hsin: 2255

Teno, Joan: 2160, 7220

Tepper, Angeles: 1255, 7265

Terashima, Rika: 7195

Terhorst, Lauren: 7490

Terhune, Elizabeth: 4190

Terracciano, Antonio: 1120, 1265, 1445, 1480

Terray, Jennifer: 7440, 7915

Terry-McElrath, Yvonne: 8010

Terzoni, Stefano: 8015

Tesar, Sarah: 2330

Testa, Alexander: 2540, 3235

Testoni, Ines: 1330

Teves, Christoph: 3035

Tewary, Sweta: 8000, 8015

Thacker, Ayush: 7185

Thackeray, Anne: 4580

Thalil, Muhammad: 4215, 7040

Than, Myint Myint: 4425

Thangwaritorn, Pilar: 4230

Thapa, Sushila: 8000

Thatcher, Christine: 8005

Thayer, Erin: 1215

Thayer, Rachel E: 7200

Theeke, Laurie: 7255, 7395

Thelin, Jacquelyn: 8005

Theorell, Töres: 8000, 8015

Thiamwong, Ladda: 1055, 2435, 3030, 7090, 7160, 7225, 7380, 7495, 7545, 8000, 8005, 8010, 8015, 9100

Thiamwong, Nicha: 8000

Thielking, Em: 7075

Thierry, Amy D: 2345

Thimmesch, Amanda: 8010

Thoene, Isabella M: 8015

Thoma, Mary: 4070, 4280

Thomas, Amy: 4445

Thomas, Cassandra: 2170

Thomas, Jonathan: 4065, 7530

Thomas, Josha: 7265

Thomas, Julia: 7330, 7390

Thomas, Kali S: 1465, 2055, 2160, 3280, 4095, 7185, 7220, 7420, 7485, 7930, 8005

Thomas, Karuna S: 8015

Thomas, Kelsey R: 4570, 8005

Thomas, Lauren: 4330

Thomas, Nelson: 8010

Thomas, Patricia A: 2295

Thomas, Robert J: 1510, 7015

Thomas Hebdon, Megan: 8010

Thomas Tobin, Courtney S: 3265, 4110, 4125, 4250

Thomeer, Mieke B: 1405

Thompson, Alexandria: 8010

Thompson, Atalie C: 1075, 4065

Thompson, Edward H: 7365, 7400

Thompson, Emily: 2445

Thompson, Hannah: 8010

Thompson, Hilaire J: 7415

Thompson, Katherine: 2205

Thompson, Ladora: 1450, 8015

Thompson, Lena: 2135, 3165, 4350, 8005

Thompson, Louisa I: 7290, 8015

Thomson, Travis: 8005

Thorn, Joanna: 9100

Thorngthip, Sutthinee: 8010

Thornton, Howard: 2515

Thorpe, Joshua: 4430

Thorpe, Lorna: 2380

Thorpe, Roland J: 1505, 3180, 3205, 3235, 3345, 3395, 4095, 4125, 4250, 9040, 3060, 3265

Thorud, Jennifer: 7475

Thuemling, Teresa: 1035, 7415

Thumuluri, Deepthi: 8000

Thyagarajan, Bharat: 1200, 2040, 2345, 7010, 7345, 7425, 8005

Tian, Qu (Teresa): 4495

Tian, Zibo: 8010

Tiburcio, Alan r: 8010, 9005

Tice, Abigail: 3030, 4450, 7090, 7160, 7380

Tice, Maria C: 7005

Tiede, Ben: 9010

Tiemeier, Henning: 1225

Tietjen, Amy: 8010

Tietz, Sarah: 1085, 7290

Tietz, MD, Sarah: 3300

Tighe, Caitlan: 7390

Tijerina, Brenda: 3305

Tilley, J. Lucas: 1095

Tilley, Sarah: 7120

Tillinghast, Anna C: 8010

Timmins, Alexandra M: 4350

Timmons-Crear, ZaKiyah L: 7015

Timonen, Virpi: 3145

Tinetti, Mary: 4445, 7030

Tinker, Samantha: 8015

Tinney, Emma: 7265

Tipaldo, Jenna F: 3385

Tipiani, Dante: 1225

Tischler, Victoria: 1210

Tisminetzky, Mayra: 9095

Titelman, Elena: 1090, 7450

Tjønneland, Anne: 7345

Tjaden, Ashley H: 1455

To, Louis: 7405

Tobar-Mosqueira, Ruy: 7440

Tobias-Wallingford, Hannah: 1520

Tobin, Jonathan N: 1170

Tobin, Karin E: 2030

Tobyne, Sean: 8000

Todd, Chris: 9085

Toh, Wei Xing: 3275

Tolea, magdalena I: 2170

Toles, Mark: 7105, 7385

Toliver, Domenic: 8015

Tollock, Maria: 7330

Tolson, Michele: 3310

Tom, Sarah: 7095

Tomar, Tanvi: 8015

Tomasic, Chloe: 8005

Tomasino, Debra: 7090, 8015

Tommet, Douglas: 2505, 4540

Tompkins, Cathy: 4600, 7005

Tong, Sebastian: 4535

Tong, Wing H.: 4105

Tong, Yuying: 4315

Tongsiri, Sirinart: 2435

Tonkikh, Orly: 2450, 4145

Tooze, Janet A: 7480

Topaz, Maxim: 7545

Topping, Michael: 7040

Torres, Camilla: 7545

Torres, Daniella: 8010

Torres, Jacqueline M: 3050, 4070, 4280, 8000, 9090

Torres, Sandra: 3335

Torres Ruiz, Ilse: 7300

Tortora, Carla: 7135

Tosato, Matteo: 8010

Tosato, Matteo: 8015

Toseef, Mohammad Usama: 1370, 2260, 8000

Tosi, Dominique M: 3065

Tosi, Hannah: 8000

Toto, Pamela E: 2445, 4305, 7515

Touhiduzzaman, MD: 4200

Towe, Maria: 8010

Towns, Juell M: 1280

Townsend, Aloen L: 8015

Townsend, Nick P: 2120

Towsley, Gail L: 1300, 1435, 4555

Toyama, Masahiro: 7200, 7255

Tracy, Eunjin L: 4335, 7390, 8000

Trainum, Katie: 4065, 7230, 7385, 7420, 7910

Tramujas Vasconcellos Neumann, Lycia: 4380

Tran, Calvin K: 7130, 7200, 7225, 7255, 7300, 7905

Tran, Diep: 1355

Tran, Duyen: 7470

Tran, Dylan: 4280

Tran, Karenna: 2500

Tran, Melissa K: 1435

Tran, Rose: 1275

Tran, Thi: 8005, 9000

Tranah, Gregory J: 4275

Trane, Ralph: 4585

Traver, Anthony C: 3305

Traver, Anthony C: 9110

Travis, Levi M: 4560

Travison, Thomas G: 1255, 2505, 4460, 7385, 7465, 8010, 9095

Traywick, LaVona S: 1450, 4115, 7265

Treat, Anny: 8005, 8010

Tremblay, Roma: 7320

Trinh, Hanh: 8010

Trinitas, Joseph: 4430, 7225, 7905

Trinkoff, Alison: 8005

Trinquart, Ludovic: 4535

TRIPATHI, ALOK: 7245

Trittschuh, Emily H: 3005

Trivedi, Amal N: 8000

Trivedi, Ranak: 8010

Troeung, Lakkhina: 2435

Troost, Jonathan: 4015

Tros, Willemijn: 7010

Trotter, Stephanie: 7015, 7935

Troutman Adams, Elizabeth: 7530

Troutman-Jordan, Meredith S: 4007

Troxel, Wendy M: 2200, 9110

Truebig, Janet: 7000

Truett, Beth J: 7400

Trujillo, Janet: 7145

Truong, Brandon: 8000

Truskinovsky, Yulya: 8005

Tsai, Chu-Lin: 7090

Tsai, Jack: 3235

Tsai, Jaw-Shiun: 8015

Tsai, Li-Tang: 8010

Tsai, Pao-Feng: 8000

Tsakiridi, Anastasia: 1095

Tse, Dwight: 1350, 3360, 4300, 7375

Tse, Hin Wing: 8000

Tseng, Chi-Hong: 8010

Tseng, George: 7345

Tseng, Han-yun: 8005, 8015

Tseng, Tung Sung: 2300

Tsimpida, Dialechti: 1095

Tso, Emily: 8015

Tsoi, Tom Chun Wai: 1030

Tsotsoros, Cindy: 8000

Tsuchiya-Ito, Rumiko: 7250, 7465

Tsuda, Shuji: 7300

Tsukada, Noriko: 7160, 7370, 7405

Tsuker, Sophia: 4015, 8005

Tsutsui, Junya: 3010

Tsutsumi, Rie: 8015

Tsuzuki, Takamasa: 7480

Tu, Wanzhu: 1145

Tu, Yufang: 7220, 7330

Tucker, David W: 4425, 7495

Tucker, Gretchen G: 8015, 9005

Tucker, Katherine L: 4295

Tuepker, Anaïs: 7135

Tuft, Samantha E: 1215, 7405, 8015

Tuicomepee, Arunya: 7375

Tuladhar, Lirisha: 1395, 2325, 4365

Tullett, Alexa: 7310

Tumurbaatar, Batbayar: 8010

Tung, Chih Chun: 9005

Tung, June H C: 9085

Tung, Yen-Chen: 8015

Tungaturthy, Neha: 4155

Tuominen, Katariina: 1080

Tupler, Larry: 1235, 4010

Turan, Janet: 2140

Turcotte, Samuel: 8005

Turiano, Nicholas A: 7180, 7355, 7505, 8005

Turjeman, Sondra: 2105

Turk, Katherine: 8000

Turkelson, Angela: 4195, 4490, 7355

Turley, Niels: 7015

Turnage, Sharon: 9100

Turner, Christopher O: 7365, 7400

Turner, Natalie: 4200, 7355, 7370, 7485, 7930

Turner, Shelbie: 1210, 1240, 1505, 2280, 7105, 7350

Turner, Sidney: 4440

Tursi, Mark: 7215, 7410

Tur-Sinai, Aviad: 7090

Turyk, Mary E: 2040

Tussey, Zachary: 7340

Twamley, Elizabeth W: 8010

Twum, Emmanuella Asabea: 2540

Tyler, Denise A: 8000

Tyrell, Tahira: 8005

Tyshkovskiy, Alexander: 8005

Tzadikario, Ayala: 8015

Tzemah-Shahar, Roy: 2105, 2415, 7000, 7210

Tzeng, Huey-Ming: 7305, 8005

Tziarli, Dimitra: 2110, 2335

U

Ucar, Duygu: 2277

Uchida, Junya: 8000

Uddin, Jalal: 1000

Udeh, kingsley C: 1365, 2260

Udo, Charles: 3180

Udoh, Idorenyin I: 7145, 7375, 8000

Ueda, Takuya: 7265

Ughra, Ravjyot: 7300

Ugoh, Munachimso: 4330

Ugwu, Benita A: 7300

Ugwu, Emmanuel O: 1365

Ugwu, Lawrence E: 7405

Ukenenye, Roseline: 8010

Ukonu, Nene: 1070

Ukraintseva, Svetlana: 7185, 7415

Ulbricht, Christie: 8000

Ulloa, Gabriella: 7265

Ulmer, Cameron: 7105

Ulrich, Connie: 7205, 8000

Umans, Jason G: 7190

Umble, Karl: 8005

Umezinwa, Ndidi M: 7345

Umoh, Mfon E: 8005

Umucu, Emre: 8010

Underwood, Elisha: 4140

Underwood, Elisha: 8015

Unroe, Kathleen: 1145, 1300, 1435, 3085, 7220, 7500, 8010, 8015, 9005

Unruh, Mark A: 4135, 7250, 7500, 9090

Unsworth, Julia R: 4400, 8015

Unsworth, Mackenzie: 7150

Urbanski, Dana: 1010, 1430, 2260, 3075, 3375, 7530

Urbina, Ray G: 7120

Urlings, Judith: 1310

Utz, Rebecca L: 1025, 1030, 8015

V

Vader, Daniel: 4210

Vain, Megan: 4200

Vale, Michael T: 7110

Valenti, Korijna: 4240

Valentin, Yves: 4590

Valenzuela, Antonio: 7455

Valeriote, Ralph: 8010

Välimäki, Tarja: 3030

Vallabha, Shree: 3270

Van Bogart, Karina: 1265, 1445, 2360, 4120

van Bruggen, Sytske: 4105

Van den block, Lieve: 7220

van den Heuvel, Edwin: 2545

Van Denend, Toni: 7225

van der Steen, Jenny T: 4105, 7010, 7220

van Ede, Anna FTM: 4105

Van Etten, Emily: 8005

Van Haitsma, Kimberly L: 1300, 4480

van Hoof, Joost: 3080

Van Hook, Jennifer: 4290

Van Houtven, Courtney: 4430, 4565, 7205

Van Orden, Kimberly A: 1185, 3100, 8000

van Rossum, Erik: 7250

van Schooten, Kimberley S: 4060

van Veluw, Susanne: 9040

Van Vleet, Bryce A: 2290, 4165, 8000, 8015

Van Vleet, Samuel: 1190, 3125, 4440, 4580

Van Vleet, Samuel C: 8015, 9105

Van Zutphen, Addison: 4405

van Zyl, Carin: 7220

Vance, David E: 2395, 4570, 7245, 7280, 9125

Vance, Jarod C: 8005, 8015, 9025

VanDenberghe, Aimee: 8015

Vander Meulen, Maria: 1285

Vanderheide, Clara: 2310

VanderWerf, Jessica A: 1315, 7175

VandeWeerd, Carla: 7320

VanDusen, Amy: 7145

VanHaitsma, Kimberly S: 1005, 7305, 8015

VanHeiden, Sarah: 3085

Vanhoutte, Bram: 4325, 4580

VanSwearingen, Jessie: 8005

Varadhan, Ravi: 2510

Varma, Malvika: 8005

Varma, Sashank: 2350

Varma, Vijay R: 2400

Varzino, Michael: 1505

Vashishtha, Rakhi: 1205

Vasireddy, Sindhu: 4440

Vasquez, Alexa: 7305

Vasquez, Anjuli: 8010

Vasquez, Daniel: 8000

Vásquez, Elizabeth: 1190, 2060, 3265, 3390, 7475, 8015

Vasquez, Felix A: 8000

Vasquez, Zander: 8005

Vasquez-O’Brien, T. Caitlin: 8010

Vasunilashorn, Sarinnapha M: 2505, 8000

Vatsyayan, Aarushi: 7395, 9045

Vaughan, Camille P: 9080

Vazquez, Christian E: 2060

Vazquez-Nuñez, Karla Patricia: 7040

Vazquez-Trejo, Valeria: 7485

Veer, Rick: 8015

Vega, Nicole M: 4227

Vega, Pamela Tella: 4150

Veilleux, Megan: 8005

Velasquez, Roberto: 7230, 7305

Velazquez-Diaz, Keisha: 7410

Velez Ortiz, Daniel: 8010

Veliz, Philip: 7295

Vemuri, Prashanthi: 7025

Vemuri, Ramesh: 2555

Vences, Kelly: 4415, 7155, 7255, 7465

Venditelli, Nijah: 7410

Venditti, Elizabeth M: 1455

Venegas, Maria: 1305, 2365, 7280

Venkatram, Chinmayi: 8010

Vera Murillo, Lizbeth C: 1285

Verbanac, Tamrah L: 7470

Verbanac, Viridian: 7085

Verbeek, Hilde: 1310, 3035, 7370

Verdery, Ashton M: 1405, 4290

Verdi, Daniel: 8010

Verdi, Laura: 8015

Verdile, Erin: 4075

Vergez, Sasha M: 1170, 7440, 7545

Verghese, Joe: 4200, 4495, 7025, 8015

Verma, Ambika: 7010, 7190

Vernon, Andrew A: 8010

Verrall, Aimee: 8010, 8015

Versey, H. Shellae: 2420, 4530

Verstaen, Alice: 4515

Vetter, Michael: 7070

Vetter, Valentin M: 4490

Viale, Daniela: 7265

Vickers, Kayci L: 8005

Victor, Christina R: 1185, 4360, 8010

Vidaurri, Glycel: 8000

Vidoni, Eric: 1020

Vidot, Denise: 8010

VIEIRA, EDGAR RAMOS: 8005

Viera, Anthony: 9150

Vila Manes, Claudia: 9115

Villar, Michelle J: 2045

Villa-Sánchez, Bernardo: 7185

Vincenzo, Jennifer L: 1280

Vinge, Jill: 3065

Virata, Michael: 7245

Visaria, Abhijit: 1205, 2230, 2435

Visingardi, Joseph: 8005

Viskochil, Richard: 7395

Visovatti, Moira: 7325

Viswanathan, Anand: 9040

Vitolins, Mara Z: 1255, 7480

Vivarakanon, Phatchanun: 7035

Vivek, Sithara: 7010, 7345

Vivek-Ananth, R.P.: 7190

Vlaeyen, Ellen: 8010

Vlock, Elizabeth: 7465

Vluggen, Stan: 7250

Vo, Jess: 8005

Vo, Quynh: 2450, 4145

Vodantis, Eleni: 7005, 7935

Vogel, Barbara: 9055

Vogel, Selina: 4470

Vögele, Claus: 1210

Vogelsang, Eric M: 7180, 7355, 7900

Vogelsmeier, Amy: 8010, 9140

Volker, Michael: 1325

Von Kay, Karin: 2170

Von Visger, Tania T: 7370

Vonk, Jet MJ: 3380

Voss, Stephanie: 1255, 7265, 8000

Vovnoboy, Irina: 7245, 7915

Vozza, Katelyn: 7350

Vozzolo, Tristan R: 4200

Vrahas, Mark S: 8000

Vranceanu, Ana-Maria: 1160, 2310, 3375, 4590, 7205

Vu, Cong Nguyen: 7390

Vu, Long: 7220

Vu, Milan: 7045

Vu, Thi: 2135, 8005

Vuong, Lauren: 8000

Vupputuri, Suma: 7110

Vyavahare, Sagar: 8010

Vytla, Kavya: 9045

W

Wachana, Anne: 1280

Wachholz, Patrick: 7370

Waddington, Emily: 9005

Wade, Benjamin: 8010

Wade, Gregory: 1155

Wadsworth, Sally J: 1175, 4110

Waern, Margda: 4010, 7255

Waetjen, L. Elaine: 9150

Wagenknecht, Lynne E: 1455, 3105

Wagner, Alison: 8010, 9145

Wagner, Brittin: 8010

Wagner, Cheryl M: 8010, 8015

Wagner, Fernando A: 4070, 4150

Wagner, Jennifer: 7370

Wagner, Lisa: 7340

Wagner, Maude: 8005, 9040

Wahl, Hans-Werner: 3215, 7295, 8015

Wahlheim, Christopher: 7435, 8015

Wahrendorf, Morten: 3195

Wahring, Iris: 1350

Waights, Verina: 3080

Wain, Kris: 7085, 7910

Waite, Linda J: 2295

Wakui, Tomoko: 4590, 8010, 9065

Walaszek, Art: 1200, 8015

Waldinger, Robert J: 3330

Waldron, Danielle: 1355

Walker, Keenan A: 1155, 1200, 4495, 7040, 8015, 9045

Walker, Nalani: 7000

Walker, Rae K: 8000

Walker, Thomas: 7340

Walkner, Tammy J: 1305, 2085

Walkner, Tammy: 8005

Walkosz, Barbara J: 2485

Walkup, Michael P: 2470

Wallace, Cara L: 4430, 7220, 8000

Wallace, Gail: 9040

Wallace, Louise Wallace: 3080

Wallace, Meredith: 2230

Waller, Dylan E: 7295

Waller, Lianna: 1085

Wallhagen, Margaret I: 7530, 9060, 9090

Wallsworth, Christine: 7205, 7915

Walsh, Patrick: 1345

Walston, Jeremy D: 2510, 9045

Walters, Monica E: 2005, 7350, 7390, 7435, 8010

Walton, Quenette L: 3305

Wan, Meijun: 3320

Wan, Qiaoqin: 2125, 7265

Wan, Shaowei: 2500

Wang, Ange: 1220

Wang, Anne: 1470, 7030

Wang, Arthur: 3380

Wang, Bei-Yun: 7525

Wang, Bingwen: 7015

Wang, Boxiang: 7265

Wang, Cecilia Xi: 8005

Wang, Chenchen: 4535

Wang, Chen-pin: 4090

Wang, Chih Yu: 9030

Wang, Claire L: 1465, 2480, 4070, 4385, 7390, 9075

Wang, Cuiling: 4310, 8015

Wang, Dahua: 7185

Wang, Di: 2405, 4185, 7085

Wang, Dingyue: 3355

Wang, Dongmin: 4255

Wang, Emily A: 8000

Wang, Ethan B: 7295

Wang, Fei: 3090

Wang, Gavin: 8010

Wang, Ge: 8015

Wang, Gemma: 8005

Wang, Guanzhou: 7240, 8000

Wang, Hairong: 8015

Wang, Haowei: 2345, 3020, 8005

Wang, Hongmei: 3320

Wang, Hongwu: 2290

Wang, Jen-Yu: 8010, 8015

Wang, Jiankun: 3090

Wang, Jiaqing: 7015

Wang, Jing: 1395, 1400, 2150, 2255, 2475, 3015, 4005, 4170, 4330, 7030, 8000, 8005, 8010

Wang, Jingxuan: 4280

Wang, Julian: 3035, 7465

Wang, Juwen: 1125

Wang, Kaipeng: 1145, 2150, 4040, 8005

Wang, Kairong: 8005

Wang, Karen: 9125

Wang, Kuan-Yuan: 9070

Wang, Kun: 1105, 7085, 7295, 7485, 9030

Wang, Leping: 3195

Wang, Lihua: 8005

wang, linying: 3075

Wang, Maggie: 2395

Wang, Mengru: 8000, 8005, 8015

Wang, Min: 8000

Wang, Nia Yuqing: 9070

Wang, Nili: 7220

Wang, Olivia (YuHsuan): 8010

Wang, Panpan: 2305

Wang, Peng-Li: 7370

Wang, Peter: 8000

Wang, Qianyun: 7055, 7900, 8000, 8010

Wang, Qingwei: 7010, 7015, 7255

Wang, Rui: 8010

Wang, Senhu: 7050

Wang, Shan: 7315

Wang, Shangqiguo: 9085

Wang, Shu: 8010

Wang, Shuai: 7015

Wang, Shuaijie: 7225

Wang, ShuangShuang: 1335, 8010

Wang, Shuo: 7345, 7425, 8015

Wang, Sijiu: 1245, 2185, 4055, 7470

Wang, Sophia: 3085

Wang, Suyi: 8005

Wang, Sylvia: 8005

Wang, Tingmei: 7015

Wang, Weidong: 7370

Wang, Weijun: 7380

Wang, Wei-Lin: 2005, 2400

Wang, Weiming: 7255

Wang, Wendy: 4375

Wang, Wenjin: 1135, 3125, 7360

Wang, Wuqi: 8005

Wang, Xi: 7330, 7535

Wang, Xiafei: 7470

Wang, Xiao (Joyce): 1270, 2160

Wang, Xiaochuan: 8000

Wang, Xiaofeng: 9150

Wang, Xiaoling: 2475

Wang, Xingzhi: 1180

Wang, Xinyi: 1235

Wang, Xuechun: 7325

Wang, Xuzhi: 8000

Wang, Yahui: 7295, 7915, 9060

Wang, Yanfeng: 7410

Wang, Yang: 1160, 2105

Wang, Yang: 7340

Wang, Yanhong: 7125

Wang, Yanxin: 2510

Wang, Yanyan: 8005, 8010, 8015

Wang, Yi: 1180

Wang, Yi: 1250

Wang, Yi: 1375, 3200

Wang, Yi: 7005, 7100, 7260, 7290, 7375, 7925

Wang, Yihan: 4425

Wang, Yijun: 8000

Wang, Yilin: 4530

Wang, Yiwen: 1125

Wang, You: 8010

Wang, Yu: 8010

Wang, Yueh-Tzu: 7165

Wang, Zengyan: 8005, 8015

Wang, Zhangyang: 8000

Wang, Zhendong: 8015

Wang, Zhiwen: 9065

Wang, Zih-Ling: 3205, 7185, 7415

Wangi, Karolus Yosef Woitilia: 4480

Wangqin, Runqi: 9035

Wanigatunga, Amal A: 2100, 3105, 4375

Ward, Caitlin: 3220, 7000

Ward, Michelle K: 7185, 7255, 7260

Ward, Nicola Ann: 4205

Ward, Rachel: 8010

Wardlow, Liane: 7020

Ware, Deanna: 2140

Warne, Donald K.: 7415

Warner, David F: 2295, 7220

Warner, Lisa M: 4300, 7395

Warner, Mildred E: 4130

Warnock, Jenna: 8010

Warren, Aaron: 7265

Warren, John Robert: 2120, 7010, 7040

Warren, Kathryn: 4410

Warry, Wayne: 4045

Wartko, Paige: 8005

Washington, Ashley: 7280

Washington, Karla T: 2320, 7220, 7260

Wasserman, Bruce A: 4375

Watanabe, Kengo: 7090, 7190, 7315

Watanabe, Kentaro: 4590

Watanabe, Kiriko: 8015

Waterhouse, Jodi L: 1130, 2070, 4355, 7290

Waters, Catherine: 9000

Waters, Leland: 1005, 1260, 2110, 2205, 7095, 8015

Watkins, Glenda: 1260, 7340

Waton, Aileen: 8015

Watson, Brittany: 7415

Watson, Gwendolyn Paige: 3340

Watson, Jacob C: 4545

Watts, Amber: 4230, 4585, 7265

Wawrzynski, Sarah E: 2030

Wayne, Peter: 4255

Wayt, Cordelia: 1390

Weaver, Ashley: 7165

Weaver, Destiny J: 7310

Weaver, Matthew D: 7390

Weaver, Raven: 3295, 4565, 7175, 7920

Webber, Madeline: 3125

Webber, Sophia: 1130, 3155

Webster, Amy: 1470

Webster, Micah: 8010

Webster, Noah: 2325, 4285

Webster, Sam: 7455, 7935

Webster-Dekker, Katelyn E: 7265

Wec, Aleksandra: 1360, 2520

Weech-Maldonado, Robert: 1150, 3365

Weems, Jacy: 1270, 7220

Wehe, Hillary: 7380

Wehry, Susan: 8010

Wehry, susan: 8015

Wei, Andrea: 9095

Wei, Ashley Z: 8005

Wei, Aybey Amy: 9035

Wei, Cindy: 3305

Wei, Duo Helen: 7015, 7385

Wei, Feng: 7015

Wei, Huichuan: 7340

Wei, Jeanne: 7005, 7010, 7190, 8015

Wei, Linjiang: 1235

Wei, Melissa Y: 8010

Wei, Shuaifang: 8005

Wei, Sijia: 7185, 8000

Wei, Wanrui: 8000, 8005, 8015

Wei, Wenxing: 1105, 3245, 3300, 8015

Weibel, Maria: 8000

Weibel, Robert: 4270

Weidmann, Rebekka: 3270

Weigand, Alexandra: 4570, 8005

Weigler, Elizabeth: 1160

Weil, Joyce: 2515, 7445

Weimar, Lisa: 3220, 7000

Weinberg, Janice: 4535

Weiner, Deborah: 7205

Weinhardt, Michael: 3080

Weinkove, David: 7190

Weir, David R: 4205

Weir, Rodney: 8005

Weisenfeld, Dana: 4460

Weisenfeld, Jonathon: 8005

Weiskittel, Skylar E: 8010

Weiskittle, Rachel: 4575, 8005

Weiss, Charlotte R: 8000

Weiss, David: 1210

Weiss, Erica: 8015

Weiss, Jordan: 3105

Weissberger, Gali: 1100, 2390

Weisse, Carol S: 4510, 8015

Weisskohl, Jack: 1260

Weldrick, Rachel: 2515

Wellenstein, Kerry: 4227, 7315

Weller, Christian E: 3310

Wells, Ashley: 1075

Wells, Jenna L: 1275, 2095, 4085, 7290, 8010, 9000

Wen, Chiung-Jung: 8015

Wen, Frances: 7005

Wen, Jiebing: 9090

Wen, Ming: 4315

Wen, Tong: 9075

Wen, Tzai-Hung: 7465

Wen, Wan: 7295

Wen, Xinyue: 9075

Wendte, Jered: 1260

Weng, Hui-Ching: 7470

Wennerdahl, Carrie L: 7410

Werfelli, Howaida: 1135

Werner, Nicole E: 1035, 2270, 2520, 7305, 7415

Werner, Shoshi: 8005

Wershof Schwartz, Andrea: 8000

Wessinger, Chadsley M: 7435, 8005, 9025

West, Brady: 7205

West, Emily: 8000

West, Jessica S: 7530, 8000, 8005

West, Tim: 7010

Westanmo, Anders D: 3065

Westcott, Jordan B: 7255, 7375, 8005

Westenskow, Rebecca: 8010

Westergard, Angela: 4475

Western, Elizabeth C: 4240

Western, Max J: 2120, 8010

Westlund, Erik: 3345

Westover, M. Brandon: 7015

Wethington, Elaine: 1055, 4285, 7540

Wettstein, Markus: 1210

Wettstein, Markus: 8010

Weuve, Jennifer: 2000

Whaikid, Phatcharaphon: 7130, 7430

Whang, Priscilla H: 4110

Wharton, Whitney L: 1420

Wheeler, Anne C: 8010

Wherry, Sarah J: 7165, 8005

Whesu, John: 8005

Whetung, Cliff: 2530, 3045, 8000

Whibley, Daniel: 7390

Whitaker, Kara M: 7265

Whitbourne, Stacey B: 8010

Whitbourne, Susan K: 1130, 2145

White, Carole: 3355

White, Christian: 7340

White, Corinne: 2310

White, Elizabeth: 7125, 7540

White, Julie: 8010

White, Kina: 1335

White, Mathew: 3145

White, Mel: 8015

White, Rebecca E: 1325, 8005

White, Suzanne M: 7015, 7920, 7935

White Makinde, Keisha: 7260

White Whilby, Kellee: 4125

Whitehead, Ishbel Orla: 7400

Whitehouse, Christina: 8000

Whitfield, Keith E: 3175

Whitlatch, Carol J: 3375

Whitley, Deborah: 2340

Whitman, Ethan: 7025

Whitman, Kathrine: 8000

Whitmer, Rachel: 8010

Whitmore, Celina: 3005, 4350

Whitmore, Corrie: 3295

Whitsel, Eric A: 7480

Whitson, Heather: 1020, 2510, 7205, 9150

Whittier, Jordan M: 4510

Whyman, Jeremy D: 7310

Wickersham, Claire E: 3000

Wickliffe, Jeffrey: 2275

Wickwire, Emerson M.: 2035, 8015

Wideman, Laurie: 8005

Widiyaningsih, Widiyaningsih: 7475

Wiederholt, Senta: 7135

Wiemers, Emily E: 3050, 8005

Wiendrick, Jack: 1460

Wiese, Lisa A: 1020, 2170, 4045, 4450

Wiggins, Alexander: 8005

Wihry, David: 7455, 7935

Wiktorsson, Stefan: 4010, 7255

Wilber, Kathleen H: 2385, 2390, 3300, 7085, 7230

Wilcock, Donna M: 2460

Wilcox, Elsa: 8010

Wild, Tess: 3275

Wiles, Lynn: 8015

Wiley, Christopher D: 4347, 8005

Wiley, Elise: 7225, 7905

Wilhelm, Myia: 3035

Wilhelmi, Morgan: 2255

Wilhelmi, Olga: 3235

Wilhite, Myrita: 7455

Wilkins, James M: 1340, 4090

Wilkins, Jessica E: 4240

Wilkinson, Katherine: 7150

Wilkinson, Lindsay R: 7130, 7475

Wilks, Scott: 7350

Willard, Meredith A: 7180, 7355, 7505, 8005

Willcox, Bradley J: 1065, 7240

Williams, Alisha: 7300

Williams, April R: 8010

Williams, Betsy: 8015, 9050

Williams, Brie: 8000

Williams, Christine L: 1020, 4045

Williams, Christopher: 7330, 7390

Williams, David M: 9130

Williams, Don’aa: 8010

Williams, Ginger: 4075

Williams, Ishan C: 4045

Williams, Johnathan: 1260, 7095

Williams, Kenneth: 8005

Williams, Kristine N: 1150, 1515, 2225, 7250, 8000

Williams, Michael: 8015, 9050

Williams, Michael P.: 4590

Williams, Rhiannon: 3255

Williams, Rua: 7015

Williams, Ryan Blake: 8000

Williams, Victoria J: 4585

Williams Sites, Kathryn: 7350

Williamson, Jeff: 2410

Williams-Sites, Kathryn: 8000

Williger, Bettina: 8010

Williman, Marty: 2485

Willis, Allison: 8005

Willis, Alyssa: 8000

Willroth, Emily C: 1045, 2155, 4110

Wilmoth, Janet M: 2085

Wilson, Annalee: 4075

Wilson, Charlton: 2170

Wilson, DaiLene: 7280

Wilson, Linnea M: 1485

Wilson, Nikki-Anne: 1280, 4470

Wilson, Skylar: 1095

Wilson, Stephanie J: 8010

Wilson, Tara: 9125

Wilson, Tracey: 1155

Wilson, Traci L: 9075

Winberry, Joseph: 7110

Winberry, Joseph: 8005

Windham, B. Gwen: 2535, 3105, 7025, 7325, 7495, 7545, 8010

Windsor, Winrose: 7225

Wingood, Gina: 2395

Wingood, Mariana: 1110, 4525, 8010

Wininger, Claire: 8005, 8010

Winogora, Victoria M: 7305

Winstead, Vicki: 4085

Winters, Kathryn: 8015

Wirba, Solee: 9130

Wirth, Maria: 4270

Wisdom, Kimberlydawn: 7390

Wisniewski, Thomas: 7010

Wister, Andrew: 7470, 7900, 8010

Witkowski, Jacek: 7315

Wittenberg, Grace: 2055, 8015

Witt-Swanson, Lindsey: 4435

Witzel, Dakota D: 1175, 2280, 7115, 7335, 8015

Wladkowski, Stephanie P.: 2265, 4430, 7220

Woestman, Jenna: 3005, 4350

Wohns, Nicolai: 8010

Wojczynski, Mary K: 7345, 8005

Wolf, Bethany: 9035

Wolf, Jack M: 1430, 2260, 3020, 3075, 3190, 7300, 7530

Wolf, Michael S: 4525

Wolfe, Alex: 7130

Wolfe, Hannah: 1045

Wolfe, Joseph D: 1405

Wolfe, Megan: 3000

Wolff, Allycia: 3375

Wolff, Jennifer: 1360, 2375, 2520, 3315, 4450, 8005

Wolff, Julia: 8010

Wolf-Ostermann, Karin: 1310, 3035

Wolfson, Emily A: 8000

Wollney, Easton: 7350

Womack, Julie A: 7245

Won, Chang Won: 7325

Won, Chang Won: 8010

Wondimu, Betselot: 7535, 7900

Wong, Alex: 1300

Wong, Alice C.: 2315

Wong, Bonnie: 7435

Wong, Carin: 8000

Wong, Carol Ka-Po: 7395, 7440

Wong, Cindy: 7205

Wong, David W: 4030

Wong, Edwin Lok Yan: 7255

Wong, Eliza Lai-Yi: 7145, 7395, 7440

Wong, Gloria Hoi Yan: 1235, 4455, 7255, 8015

Wong, Jennifer A: 2380

Wong, Jennifer: 7525

Wong, Joey Oi Yee: 2090, 7235, 8010

Wong, Karen Lok Yi: 7235

Wong, Karen Lok Yi: 8010

Wong, Lena L N: 9085

Wong, Lily: 7205, 7915

Wong, Megan: 4227, 7315

Wong, Rebeca: 4470, 7445

Wong, Rebeca: 8010, 9075

Wong, Roger: 2140, 7185

Wong, Stella Lai-Kuen: 1135

Wong, Stephanie Ming Yin: 1235, 4455, 7255

Woo, Jean: 2455, 8005

Woo, Joyce: 8005

Woo, Seoyoon: 7005, 7400

Wood, Kristie: 2095

Wood, Leila: 7405, 9025

Wood, Madeline: 8015

Wood, Michele: 8015

Woodhouse, Mark: 1455, 2160

Woodrell, Christopher: 9145

Woods, Carly: 8005

Woods, Diana L: 8010

Woodward, Amanda T: 8000, 8010

Woodward, Aubrie: 1400, 8010

Woolley, John: 7435

Woolrych, Ryan: 1325

Woolson, Sandra: 4430

Woolverton, Cindy B: 8000, 8005

Wormwood, Jolie: 4090

Worthington, Amber: 1120

Wren, Jonathan D: 3127, 8005

Wright, Bernadette: 1370, 1465

Wright, Eric R: 4550

Wright, Julia: 8000

Wright, Kathy D: 2135, 8010, 9030

Wright, Lauri: 4095

Wright, Molly A: 8005

Wright, Morgan M: 2010

Wright, Rebecca: 7430

Wright, Regina S: 7465

Wright, Shawna: 8000

Wroolie, Tonita Emily: 2240

Wrosch, Carsten: 7390

Wrublowsky, Robert: 2080

Wu, Barry J: 8005

Wu, Bei: 1400, 1450, 2475, 3015, 3110, 3290, 4055, 4170, 4330, 4505, 7030, 7415, 7485, 8000, 8005, 8010, 8015, 9035

Wu, Chao-Yi: 2360, 3090, 7415

Wu, Chenkai: 2510, 7365, 8005

Wu, Deqing: 7415

Wu, Fei: 7010

Wu, Heng: 7090

Wu, Hongchen: 2350, 7525

Wu, Huijun: 1245

Wu, Jialin: 8015

Wu, Jing: 7225

Wu, Jinhui: 8000

Wu, Jinyan: 9020

Wu, Katherine: 2140

Wu, Kuan-Ching: 7205, 7395

Wu, Lei: 8000

Wu, Li: 1220

Wu, Lihua: 3075

Wu, Mingche MJ: 2520

Wu, Niels: 3250, 4410, 7005

Wu, Qinyang: 7410

Wu, Rui: 7470

Wu, Samantha: 2300, 7100

Wu, Shih-Cyuan: 7370

Wu, Shu-Ying: 1365, 2135, 8005

Wu, Shwu-Chong: 7370

WU, Vivien Xi: 7040

Wu, Wen-Chih: 7000

Wu, Wen-Chih: 8015

Wu, Xiaochu: 8010

Wu, Xucheng: 9150

Wu, Yanjun: 7325

Wu, Yifei: 7225

Wu, Yingyan: 4540

Wu, Yiwen: 3360, 7005, 9070

Wu, Yiyi: 2395

Wu, Yiyuan: 3300

Wu, You: 1245, 9000

Wu, Yuewen: 7255

Wu, Yuhan: 7010

Wu, Zhen: 2305, 7145

Wurm, Susanne: 8010

Wurtz, Heather: 9080

Wyatt, Tami H: 8000

Wylie, Glenn R: 8015

Wylie, Molly J: 7280, 7360

Wyman, Mary F: 3005, 4350

Wyman, Michelle: 7305

Wyrostek, Lyndi: 2485

X

Xavier, Rose Mary: 8010

Xi, Wanyu: 2370

XIA, Keting: 7045

Xia, Maoyao: 9150

Xia, Weiyi: 9045

Xiang, Jaiwei F: 7460

Xiang, Xiaoling: 3175, 8015

Xiao, Chunhong: 8000

Xiao, Jinyi: 7275

Xiao, Junhua: 7340

Xiao, Lulu: 7145

Xiao, Qian: 3185

Xiao, Wendong: 2125

Xiao, Xaviera: 4225

Xiao, Xiaohua: 2275

Xiao, Yexuan: 7015, 7095

Xie, Alice: 9105

Xie, Bo: 3340, 4065, 7015, 7230, 7385, 7910

Xie, Fei: 7125

Xie, Hui “Jimmy”: 4470, 7210

Xie, J. Kathy: 7115

Xie, Kathy: 8000

Xie, Kristal: 8015

Xie, Liujing: 8015

Xie, Rui: 2435, 3030, 4450, 7090, 7160, 7380, 7495, 8005, 8015, 9100

Xie, Serena Jinchen: 4180

Xie, Yuning: 7145, 8005

Xing, Weijie: 7015

XING, YANQIU: 7190

Xing, Yiwen: 8005

Xing, Yunli: 7090, 7320

Xiong, Jiaming: 8005

Xiong, Xuechen: 1030

Xu, Beibei: 2545, 7320, 7520

Xu, Cai: 1235

Xu, Chuanzheng: 1430

Xu, Chun: 7190

Xu, Dongjuan: 7370

Xu, Furong: 8010

Xu, Hanzhang: 2150, 3135, 4095, 4170, 4505, 7520, 8000, 8005, 8015, 9035

Xu, Huiwen: 4365

Xu, Jiahui: 4330

Xu, Jiajia: 7240

Xu, Jianhong: 2510

Xu, Jiayun: 2255

Xu, Kailin: 9070, 9080

Xu, Kelin: 7320

Xu, Ling: 8010

Xu, Mengya: 2005, 2400

Xu, Ming: 8005

Xu, Qin: 2545, 4505, 7090

Xu, Shicheng: 8000, 8005, 8015

Xu, Shu: 1075, 4065, 7275

Xu, Shuo: 3320, 7220

Xu, Sophie Zhijing: 2475, 4505, 8010

Xu, Ting: 4055, 4505, 7090

Xu, Wei: 2350

Xu, Wei: 7260

Xu, Weili: 4370

Xu, Wenqian: 3020

Xu, Winnie: 8000

Xu, Xinming: 7320

Xu, Xinyi: 2545, 7090

Xu, Yanxun: 7245

Xu, Ying: 1225, 7085, 7375, 7470

Xu, Yingzhi: 8010

Xu, Yuelong: 8000, 9050

Xu, Zhijing: 3110, 8010

XUE, Dandan: 7305

Xue, Lingshu: 1385

Xue, Qian-Li: 1220, 2100, 2400, 2510, 8000, 8005

Xue, Tingzhong (Michelle): 2465, 3135, 9035

Xue, Wei-Lin: 8010

Y

Yablon, Yaacov: 8015

Yabuki, Tomoyuki: 8010

Yadav, Hariom: 7190

Yadollahikhales, Golnaz: 8010

Yafeh Mfuh, Anita: 8005

Yaffe, Kristine: 4335, 7000, 8005

Yahaya, Ahmed-Rufai: 8015

Yaisikana, Mo’e: 4325

Yajima, Masanao: 1100

Yalcin, Damla: 7315

Yamada, Ryousuke: 8000

Yamada, Yuji: 7195

Yamakawa, Miyae: 7195

Yamamoto, Yasuhiro: 7380

Yamamoto-Mitani, Noriko: 2210, 7440, 8000, 9020

Yamashita, Takashi: 7035, 7115, 7145, 7900, 8000, 8005

Yan, Alice: 7485

Yan, Cheng: 8010

Yan, Elsie: 1215, 2325, 7405, 8005

Yan, Haijuan: 2085

Yan, Jinghua: 7320

Yan, Kailei: 7425

Yan, Mengzhao: 1320, 7300, 8005, 8015

Yan, Shibin: 1205, 7300, 7535

Yan, Xumeng: 4125, 4250

YAN, Zi: 1205, 4055, 7300, 7405

Yanez, Romel: 1135

Yang, Betsy: 3065

Yang, Biying: 7150

Yang, Chih-Hsiang: 7435

Yang, Chun-Ting: 1340, 4090

Yang, Eunhwa: 7160

Yang, Frances M: 1150, 1515, 2225, 3255, 7250, 8000, 8010

Yang, Hayejin: 7300, 7375

Yang, Hee Won: 3235

Yang, Hongyan: 7080

Yang, Huayu: 7315

Yang, Hyewon: 3270

Yang, Janet: 2240

Yang, Jie: 1340

Yang, Jie: 8005

Yang, KwangSoo: 2045

Yang, Kyeongra: 8000, 9050

Yang, Mingzhu: 8010

Yang, Minhee: 7185, 7295, 7330, 7525, 8010

Yang, Qian: 2275

Yang, Qiaohong: 7410

Yang, Qifan: 7010

Yang, Qing: 3355, 4095

Yang, Ruimin: 7535

Yang, Ruochen: 9105

Yang, Seungbum: 7225, 7300, 7375

Yang, Shuqi: 7015

Yang, Siying: 2185

Yang, Wei: 3225, 7240

Yang, Xinyue: 7185

Yang, Yang (Claire): 1495, 3195

Yang, Yijian: 4060, 7090, 7225, 8000

Yang, Yiqing: 8005

Yang, Yong: 1355

Yang, Yulin: 1370, 4070

Yang, Yunjia: 7265

Yang, Yuting: 1285

Yang, Zhou: 2545

Yang, Ziyi: 7010, 7910

Yankeelov, Pamela: 1060, 2465

Yankson, Spendilove: 7240

Yao, Jie: 2475

Yao, Jie: 9155

Yao, Jingxin: 4565

Yao, Li: 7215, 8010

Yao, Shanshan: 2000, 2245, 7345, 8010

Yao, Yao: 7225

Yap, Xin Yi X: 7040

Yarbrough, Courtney: 1080

Yarnall, Jessica: 4560

Yarrow, Linda: 7130

Yashin, Anatoliy I: 7185, 7415

Yashkin, Arseniy: 1235, 4010, 4460, 7185

Yasumoto, Saori: 3010

Yasunaga, Hideo: 9020

Yau, Jessie Ho-Yin: 1135

Yauk, Jessica: 7220

Yazdani, Neshat: 7040

Ye, Fan: 4200

Ye, Jiangfeng: 8005

Ye, Jing: 1430, 7080

Ye, Lichuan: 4265

Yeargers, Jon: 7545

Yee, Claire I: 1275, 4085

Yee, Nicholas J: 7370

Yeh, An-Yun: 7255

Yeh, Jarmin C: 2515, 4160, 7215, 7925, 8000, 9000

Yeh, Shu-Chuan Jennifer: 7515

Yeh, Yen-Po: 7140

Yellon, Tamar: 8005

Yeo, Hyesu: 7325

Yeo, Shumian: 8005

Yeoh, Eng-Kiong: 7395, 7440

Yeom, Hyun-E: 7220, 7425, 7430

Yeong, Joe: 8005

Yero, Ivana: 8005

Yeung, Dannii: 2350, 4300, 7295, 7395, 7915, 8005, 8015, 9060

Yeung, Wei-jun Jean: 1240

Yew, Pui Ying: 7305

Ygrubay, Pamela K: 2245

Yi, Dahyun: 3380

Yi, Grace: 3325, 7340, 9100

Yi, Mo: 4335, 9065

Yi, Stella S: 2380, 3155

Yi, Xingnan: 1030

Yi, Xinyu: 8010

Yi, Yang: 2125

Yi, Zhiqi: 2525, 3320, 7110, 7220, 7405

Yildiz, Ferhat: 8000

YILDIZ, MUSTAFA: 7385

Yilmaz, Yasemin: 8000

Yin, Cheng: 7250, 7380, 7430

Yin, Diana: 3215

Yin, Jiyuan: 8015

Yin, Lijuan: 1160, 3355, 7015

Yin, Na: 3385

Yin, Qingqing: 7090, 7260

YIN, SHI: 8005

Yin, Ying: 8010

Ying, Dire: 2125

Ying, Gelan: 3380, 4540

Yip, Samantha: 7525

Ylitalo, Kelly: 4460

Yockey, R. Andrew: 7540

Yoder, Grant: 2320

Yokoyama, Yuri: 7265

Yolken, Robert H: 7040

Yonashiro-Cho, Jeanine: 3300

Yoneda, Tomiko: 1265, 4120, 4515, 8015

Yoo, Eun-Young: 4525

Yoo, Haeun: 7115, 7230

Yoo, Jaeon: 7080

Yoo, Ji: 8005

Yoo, Leeho: 8010

Yoo, Yaeeun: 7065, 7365

Yoo, Youngjun: 8015

Yoo-Jeong, Moka: 1155, 9110

Yoon, Caroline: 7075

Yoon, Dokyung: 7395

Yoon, Hee Jeong: 9060

Yoon, Seolah: 7520

Yoon, Sukyung: 7375

Yoon, Sumin: 1090

Yoon, Young Ji: 7295

York, Amy M: 2355

Yoshie, Satoru: 8000, 8010

Yoshikawa, Aya: 8010

Yoshimitsu, Takahashi: 2230

Yoshioka-Maeda, Kyoko: 7440

Yost, Govinda: 1455

You, Sang Bin: 8005, 9135

Young, Bailey A: 8000

Young, Heather M: 2450, 4145, 7085, 7915

Young, Kate: 7090

Young, Meghan: 4345

Young, Sarah R: 4525

Young, Veronica: 8015

Young, Yuchi: 7220, 7330

Yow, W. Quin: 3090, 3215, 4560, 8015

Yu, Chang: 4185, 4590, 7040

Yu, Chen-Wei: 2095

Yu, Danxia: 9030

Yu, Dayoung: 7545

Yu, Doris S. F.: 1225, 2545, 4065, 9085

Yu, Gongda: 4590

Yu, Heshuo: 7055

Yu, Hongyu: 7410

Yu, Hsiao-Wei: 7140, 7370

Yu, Jiani: 3300

Yu, Jiao: 1180, 1505, 4580

Yu, Jie: 7305

Yu, Jiening: 7240

Yu, Jihnhee: 7370, 7380

Yu, Junwen: 1245, 4055

Yu, Junwen: 8005

Yu, Kexin: 1185, 2360

Yu, Mingzhi: 7225

Yu, Pei-Ying: 7080

Yu, Tiffany: 7370

Yu, Wanting: 7025, 7295, 7465, 8010

Yu, Wenhua: 9085

Yu, Wenshan: 8000

Yu, Xuexin: 4455, 9040, 9120

Yu, Zhenhan: 7225

Yu, Zilong: 4225

Yuan, changzheng: 7090

Yuan, Jiameng: 2150, 3135, 4505

Yuan, Yan: 8015

Yuan, Yaqi: 1370

Yuan, Yaqun: 1500, 7345

Yuan, Yarou: 9020

Yuan, Yiyang: 7255, 7415

Yuan, Zhuochen: 7530

Yue, Yiwei: 7040

Yuh, Esther L.: 7000

Yun, Hyunkyung: 1385, 7250, 7540

Yun, Ji-Young: 1345

Yun, Jung eun: 8005

Yung-Choy, Rebecca: 7205

Yunru, Chen: 4370

Yurkiv, Halyna: 1430

Yusuf, Khalid: 8015

Yuwen, Weichao: 4180

Z

Zadeh, Rana: 7455, 7915

Zafar, Anum: 3205, 8005, 9045

Zafar, Sahar: 7015

Zagorski, William: 4145, 4160

Zaguri-Greener, Dalit: 8005, 9000

Zahm, Kathryn: 9120

Zahodne, Laura: 3380, 7435, 8010

Zahradka, Nicole: 7300

Zajacova, Anna: 1070

Zakrajsek, Jennifer S: 4435

Zaman, Kimia Tuz: 3340, 7015, 8000

Zaman, Shahid: 8000

Zambrano Garza, Elizabeth: 7390

Zamrini, Edward: 2460, 8010

Zanasi, Francesca: 9020

Zanchelli, Michael C: 8010, 8015

Zang, Emma: 4185, 7325

Zang, Shenghao: 4080

Zanier, Nicole: 4435

Zaninotto, Paola: 1040, 2250, 3145, 7295

Zanjani, Faika: 1260

Zank, Susanne: 4470

Zannas, Anthony: 3185

Zanotelli, Zackary: 7180, 7900

Zanotto, Tobia: 4060, 7090, 7225

Zaragoza, Gabriela: 8010

Zarazua-Osorio, Brenda: 9015

Zarit, Steven H: 2385, 3185

Zaslavsky, Oleg: 7185, 7205, 7415

Zauszniewski, Jaclene A: 3300

Zavala, Daisy V: 1265

Zavez, Katherine: 7345

Žefran, Miloš: 7015

Zegarra Valdivia, Jonathan Adrian: 7010

Zeglin, Lydia: 7130

Zeid, Janna: 8005

Zeisel, John: 8005

Zeki Al Hazzouri, Adina: 8000, 8005, 9120

Zelaya, Mariel: 8010, 8015

Zelinski, Elizabeth: 7395

Zell, Angela: 4075, 7540

Zellous, Erreannau L: 3125, 8000

Zeng, Junyan: 7145

Zeng, Qing: 8010

zeng, Qingyi Jasmine: 7475, 9010

Zeng, Shengyin: 7410, 8015

Zeng, Weicheng: 7015

Zeng, Xiaoyi: 3015, 4015, 4530, 7260

Zeng, Xueling: 7245

Zeng, Ziwei: 4060, 7090, 8000

Zeng-Trietler, Qing: 9020

Zertuche, Juan-Pablo: 1070

Zervakis, Jennifer: 7350, 7930

Zettel-Watson, Laura: 7455

Zhai, Shumenghui: 4180

Zhai, Wanting: 1475

zhai, yanping: 7125

Zhan, Rongfang: 8015

Zhang, Boya: 1040

Zhang, Caifang: 7300

Zhang, Chenxin: 4310

Zhang, Cuntai: 3160, 7190, 7320, 7410

Zhang, Deqiang: 7315

Zhang, Guolei: 7300

Zhang, Hongwei: 9140

Zhang, Huan: 8005

Zhang, Jiaan: 7435

Zhang, Jianguo: 8015

Zhang, Jingyun: 7240

Zhang, Jun-e: 1245

Zhang, Kai: 2475, 8010

Zhang, Kaylee: 8015

Zhang, Kejia: 8000

Zhang, Kelvin Pengyuan: 2000, 4060, 4540

Zhang, kemeng: 4370, 7310

Zhang, Kevin: 7305

Zhang, Le: 7190

Zhang, Liangwen: 1235, 7010

Zhang, Liming: 7240, 9150

Zhang, Liwei: 8000

Zhang, Meina: 1475, 4230, 7265

Zhang, Meng: 8015

Zhang, Minqing: 7240

Zhang, Ning: 4265

Zhang, Peiyuan: 1145, 1365, 3015, 7220, 7500

Zhang, Peiyuan: 8005, 9005

Zhang, Qian: 3075

Zhang, Qiao: 7010

Zhang, Qin: 1220, 1245, 4055, 7095, 7130, 7915

Zhang, Rendong: 7545

Zhang, Rong: 4060, 7125

Zhang, Rui: 7305

Zhang, Ruoying: 3025

Zhang, Shenghao R: 1285

Zhang, Shiyang: 2025, 7390

Zhang, Shu: 7330

Zhang, Sijian: 9020

Zhang, Stephanie: 7355

Zhang, Suyanpeng: 7185

Zhang, Wei: 3320

Zhang, Weiguo: 2315

Zhang, Wen: 1235

Zhang, Wencheng: 1125, 2405

Zhang, Wenhan: 4565

Zhang, Wenhui: 8015

Zhang, Wen-Ru: 8010

Zhang, Wenting: 8010

Zhang, Wenxin: 9040

Zhang, Wuyang: 2000, 4450

Zhang, Xiangyu: 8010

Zhang, Xiao: 4470, 7210

Zhang, Xiaodan: 3090

Zhang, Xiaomin: 7010, 7190

Zhang, Xin: 3275

Zhang, Xiuhong: 7300

Zhang, Xiyuan: 4295

Zhang, Xue: 4130

Zhang, Xuehan: 8000, 8010

Zhang, Yalu: 3320

Zhang, Yan: 1160, 2105

Zhang, Yan: 4255

Zhang, Yan: 7220

Zhang, Yi: 7310

Zhang, Yichen: 7470

Zhang, Yichi: 1340

Zhang, Yiwei: 8005

Zhang, Yongkang: 3300, 7220, 7250

Zhang, Youjuan: 1515, 2315, 9050

Zhang, Yu: 1125

Zhang, Yuan S: 1495, 9040

Zhang, Yuankai: 8000

Zhang, Yuchen: 4485

Zhang, Yuewen: 8015

Zhang, Yuhe: 7015

Zhang, Zhang: 1360, 2520, 3110, 8015

Zhang, Zhe: 1140

Zhang, Zilong: 8005

Zhang, Ziqi: 7300

Zhang, Ziying: 3035, 3125

Zhao, Bochao: 2125

Zhao, Erfei: 4440

Zhao, Ershuo: 7145

Zhao, Fang: 3020

Zhao, Haijun: 7145

Zhao, Heng: 7340

Zhao, Henry: 4385, 7545

Zhao, Hui: 8010

Zhao, Jing: 3320

Zhao, Lihui: 7185

Zhao, Rui: 7430

Zhao, Shangang: 8015

Zhao, Shangshu: 4495

Zhao, Shangshu: 8010

Zhao, Xiaoyan: 7265

Zhao, Xintong: 3320

Zhao, Yang: 7310

Zhao, Yong: 7235, 7295

Zhao, Yuanchang: 8005

Zhao, Yuxiang (Chris): 2370

Zheng, Anqing: 1175

Zheng, Chunlei: 8000

Zheng, Jiashun: 1460

Zheng, Jiayu: 7015

Zheng, Judy: 7025

Zheng, Mingyang: 8000

Zheng, Ruoqi: 7380

Zheng, Shimin: 7085

Zheng, Wenqing: 8000

Zheng, Xiaoping: 4060

Zheng, Yaguang: 4170, 4505, 8005, 8010, 8015

Zheng, Yexin: 2275

Zheng, Yueyuan: 8005

Zhong, Angela: 7265

Zhong, Chuwen: 1185, 4455

Zhong, Kelin: 8000

Zhong, Roger: 8010

Zhong, Selena: 4085, 8010

Zhong, Tangsheng: 4335

Zhou, Huayu: 8015

Zhou, Jiajia: 2260

Zhou, Jiayu: 3090

Zhou, Jiayu: 8000

Zhou, Junhong: 7025

Zhou, Kaixin: 9020

Zhou, Leyi: 2540, 8015

Zhou, Lily: 7525

Zhou, Maolin: 8010

Zhou, Nan: 2020

Zhou, Peter: 2090

Zhou, Sasha: 4565

Zhou, Shuai: 1515, 2315

Zhou, Tianqi: 1225, 2405, 7085, 7375

Zhou, Timothy: 1035

Zhou, Wenjian: 7225

Zhou, Xinkai: 4375

Zhou, Yanhua: 9095

Zhou, Yanjiao: 4227

Zhou, Yatian: 3275

Zhou, Yixuan: 4280

Zhou, Yuanjin: 4015, 4530

Zhou, Yue: 7255

Zhou, Zexi: 1105, 3015, 7295, 7390

Zhou, Zhiyong: 4270

Zhu, Carolyn: 9080

Zhu, Dantong: 7090

Zhu, Haojing: 3145

Zhu, Jiakang: 7010

Zhu, Leticia: 3305

Zhu, Mengya: 7095

Zhu, Peijia: 7010

Zhu, Shida: 7410

Zhu, Shijia: 7345

Zhu, Shijun: 2350

Zhu, Wenjuan: 3075

Zhu, Xi: 3290

Zhu, Xi: 8000

Zhu, Xiaoqian: 2535, 7325

Zhu, Xuemei: 7360

Zhu, Yiqi: 8015, 9015

Zhu, Yiwen: 8005, 9155

Zhu, Yujun: 2390, 7085

Zhu, Zheng: 4055, 7015

Zhu, Ziwei: 8015

Ziemba, Yonah C: 8000

Zies, Susan: 8010

Zieschang, Tania: 4450, 7200, 7225

Zimbroff, Robert M: 2480

Zimmer, Vicki: 1160, 1225

Zimmer, Zachary: 2275

Zimmerman, Kristin M: 7340

Zimmerman, Sheryl: 1435, 3260, 3280, 7105, 7225, 7440, 7915

Zing, Xing: 4520

Zionts, Nancy: 8005, 8010

Zipunnikov, Vadim: 4375

Zisberg, Anna: 1130, 1485, 1515, 4335, 4355, 7000, 7060, 7145, 7175, 7255, 7410, 8005, 9000

Zisk Rony, Rachel Yaffa: 8005

Zissimopoulos, Julie: 2005, 2400

Zivin, Kara: 3100

Zivotofsky, Ari: 8005

Zlyesov, Eduard: 1090, 7450

Zmuda, Joseph M: 8005

Zocchi, Mark: 2085

Zoh, Roger: 3010, 7010

Zolnoori, Maryam: 7545

Zonderman, Alan: 7130

Zonker, Christina R: 1165

Zou, Baiming: 4095

Zou, Jian: 8005

Zubatsky, Max: 2110, 2335, 8000

Zuberi, Daniyal: 9135

Zuelsdorff, Megan: 1160, 8005

Zullo, Andrew R: 1435, 2465, 4010, 7345, 7385, 7510, 8005, 9020

Zuniga, Franziska: 7250

Zuo, Lihan: 3090

Zuo, You: 8000

Zuraw, Matthew: 7305, 7415

Zurlo, Karen: 4210

Zwakhalen, Sandra: 7250

Zweig, Richard: 7005

Zweig, Susan: 4170, 8015

Zwerling, Jessica: 8015

